# Early innate immunity determines outcome of *Mycobacterium tuberculosis* pulmonary infection in rabbits

**DOI:** 10.1186/1478-811X-11-60

**Published:** 2013-08-19

**Authors:** Selvakumar Subbian, Nirmalya Bandyopadhyay, Liana Tsenova, Paul O’Brien, Viraj Khetani, Nicole L Kushner, Blas Peixoto, Patricia Soteropoulos, Joel S Bader, Petros C Karakousis, Dorothy Fallows, Gilla Kaplan

**Affiliations:** 1 Laboratory of Mycobacterial Immunity and Pathogenesis, The Public Health Research Institute (PHRI), New Jersey Medical School, Rutgers Biomedical and Health Sciences, Rutgers The State University of New Jersey, 225 Warren Street, Room W250.W, Newark, NJ, USA; 2 Department of Biomedical Engineering, High-Throughput Biology Center and Institute of Computational Medicine, Johns Hopkins University, Baltimore, Maryland, USA; 3 Biological Sciences Department, NYC College of Technology, Brooklyn, NY, USA; 4 Center for Applied Genomics, PHRI, New Jersey Medical School, Rutgers Biomedical and Health Sciences, Rutgers The State University of New Jersey, Newark, NJ, USA; 5 Center for Tuberculosis Research, Department of Medicine, Johns Hopkins University School of Medicine, Baltimore, MD, USA; 6 Department of International Health, Johns Hopkins Bloomberg School of Public Health, Baltimore, MD, USA

**Keywords:** Pulmonary tuberculosis, Latent *M. tuberculosis* infection, Early innate immunity, PMN leukocyte recruitment, Macrophage activation, STAT1 network, Inflammatory response, Rabbit lung transcriptome

## Abstract

**Background:**

Pulmonary infection of humans by *Mycobacterium tuberculosis* (Mtb), the causative agent of tuberculosis (TB), results in active disease in 5-10% of individuals, while asymptomatic latent Mtb infection (LTBI) is established in the remainder. The host immune responses that determine this differential outcome following Mtb infection are not fully understood. Using a rabbit model of pulmonary TB, we have shown that infection with the Mtb clinical isolate HN878 (a hyper-virulent W-Beijing lineage strain) leads to progressive cavitary disease similar to what is seen in humans with active TB. In contrast, infection with Mtb CDC1551 (a hyper-immunogenic clinical isolate) is efficiently controlled in rabbit lungs, with establishment of LTBI, which can be reactivated upon treatment with immune-suppressive drugs. We hypothesize that the initial interaction of Mtb with the cells of the host response in the lungs determine later outcome of infection.

**Results:**

To test this hypothesis, we used our rabbit model of pulmonary TB and infected the animals with Mtb HN878 or CDC1551. At 3 hours, with similar lung bacillary loads, HN878 infection caused greater accumulation of mononuclear and polymorphonuclear leukocytes (PMN) in the lungs, compared to animals infected with CDC1551. Using whole-genome microarray gene expression analysis, we delineated the early transcriptional changes in the lungs of HN878- or CDC1551-infected rabbits at this time and compared them to the differential response at 4 weeks of Mtb-infection. Our gene network and pathway analysis showed that the most significantly differentially expressed genes involved in the host response to HN878, compared to CDC1551, at 3 hours of infection, were components of the inflammatory response and STAT1 activation, recruitment and activation of macrophages, PMN, and fMLP (N-formyl-Methionyl-Leucyl-Phenylalanine)-stimulation. At 4 weeks, the CDC1551 bacillary load was significantly lower and the granulomatous response reduced compared to HN878 infection. Moreover, although inflammation was dampened in both Mtb infections at 4 weeks, the majority of the differentially expressed gene networks were similar to those seen at 3 hours.

**Conclusions:**

We propose that differential regulation of the inflammation-associated innate immune response and related gene expression changes seen at 3 hours determine the long term outcome of Mtb infection in rabbit lungs.

## Lay abstract

Inhalation of infectious aerosols containing viable *Mycobacterium tuberculosis* (Mtb), results in symptomatic tuberculosis (TB) in about 5-10% of people, while the majority of exposed individuals develop asymptomatic, latent TB infection (LTBI). These diverse clinical outcomes following Mtb infection are determined by intricate host-pathogen interactions that are not fully understood. We have established a rabbit model of pulmonary TB that closely mimics the pathological features of human disease and LTBI. In our model, pulmonary infection of rabbits with Mtb HN878, a hyper-virulent W-Beijing strain, results in progressive cavitary disease; infection with CDC1551 is effectively cleared over time, establishing LTBI that can be reactivated upon immune suppression. In the present study, we used our rabbit model to test the hypothesis that the initial host response in the lungs within hours of infection determines later outcome. At similar infection doses, we found increased accumulation of macrophages and PMN in the lungs of HN878-, compared to CDC1551-infected rabbits, at 3 hours. Consistently, we observed activation of cellular networks involved in the inflammatory response, STAT1 activation, recruitment and activation of macrophages and PMN, and fMLP-stimulation in the lungs of HN878-infected rabbits. Similar differential expression patterns in all the tested network genes were seen at 4 weeks, with infection and pathology reduced in CDC1551-infected animals compared to HN878 infection. This suggested that the overall outcome following Mtb infection of rabbit lungs is significantly influenced by the differential regulation of inflammation-associated innate immune cells and associated gene expression changes observed already at 3 hours.

## Background

In humans, inhalation of aerosol droplets containing Mtb results in a spectrum of clinical outcomes, ranging from progressive granulomatous disease (seen in 5-10% of immune competent individuals), with continued bacillary growth and exacerbated lung pathology, to containment of infection and establishment of asymptomatic latent infection (LTBI; seen in about 90%) [1]. The determinants of outcome following Mtb infection have been shown to be dependent on the host innate immune response [2,3]. Polymorphisms in genes encoding the toll-like receptors (TLR), vitamin D receptors (VDR), and other innate immune recognition molecules have been associated with increased susceptibility of individuals to TB disease [4,5]. In addition, recent studies have suggested that the nature of the infecting bacilli also contributes to the outcome of infection [6,7]. Epidemiological studies have shown differential infectivity among various Mtb strains in the population. Genotypic analysis of 516 clinical isolates from patients showed that Mtb strains of the W-Beijing lineage caused the highest number of TB cases in Taiwan [8]. Similarly, a strong association between W-Beijing and HIV infection was reported among South African patients [9]. Furthermore, a sublineage of the W-Beijing strain has been associated with increased disease transmission [10]. However, the exact mechanism underlying this Mtb strain dependant differential response is not fully understood. To better understand the interaction between specific infecting Mtb strains and host protective immunity, we established a rabbit model of Mtb infection that mimics the full range of disease manifestations seen in humans [11-13]. In rabbits, the nature of the infecting Mtb strain significantly influences the host-pathogen interactions and determines the outcome of infection. We have used the clinical Mtb strain CDC1551, which is highly immunogenic in animals [14], to infect rabbits by aerosol exposure. Infection with CDC1551 results in early transient limited bacillary growth, followed by spontaneous clearance of organisms, as manifested by an absence of detectable colony forming units (CFU) in the lungs, liver and spleen by 12 to 16 weeks post-infection, depending on the initial inoculum [13]. This phenomenon represents true LTBI rather than tissue sterilization, since reactivation of the infection is achieved with immune suppression of rabbits with triamcinolone, a synthetic corticosteroid. In CDC1551-infected rabbits, control of infection is associated with small, well-differentiated lung granulomas and robust activation of the host antimicrobial response, characterized by peak activation of monocytes and CD4^+^ T cells by 4 weeks, that gradually declines over the next 4 to 8 weeks in parallel with declining CFU numbers. Concurrent with bacillary clearance, the granulomatous lesions resorb with time, and the lungs regain a normal appearance [11]. In contrast, infection of rabbits with the less immunogenic, but more virulent, clinical Mtb strain HN878 leads to progressive granulomatous TB. In the lungs of these animals, diverse lesions are observed, including small, cellular granulomas and larger ones with necrotic centers, as well as liquefied lesions that eventually cavitate with extensive bacillary growth at the luminal surface, similar to those seen in human pulmonary disease [11,12]. HN878 infection is associated with lung inflammation, followed by a slow and sub-optimal activation of the host innate and adaptive immune responses and the sustained presence of activated CD4^+^ and CD8^+^ T cells throughout the course of infection, which seems to be driven by the bacillary load in the lungs [12].

To gain insight into the host response that culminates in the progression of infection to active TB disease versus establishment of LTBI, we investigated the early (3 hours) and 4 week response to HN878 and CDC1551 following equivalent implantation of each Mtb strain into the lungs of rabbits. Leukocyte recruitment and granuloma development in response to Mtb infection were determined by histological analysis of lung tissue. Using rabbit whole-genome microarray gene expression analysis, we determined the differential gene expression induced in the rabbit lungs in response to infection with each of these two clinical isolates. We evaluated the ability of early (3 hour) differential changes in the host immune response in the rabbit lungs to predict later outcome following Mtb infection, by interrogating the gene networks at 4 weeks. Our results suggest that in rabbit lungs, the outcome following Mtb infection is significantly influenced by the differential regulation of inflammation-associated innate immune cells and related network gene expression changes occurring already at 3 hours.

## Results

### Early recruitment of mononuclear and activated polymorphonuclear (PMN) cells into the Mtb-infected rabbit lungs

**Figure 1:**
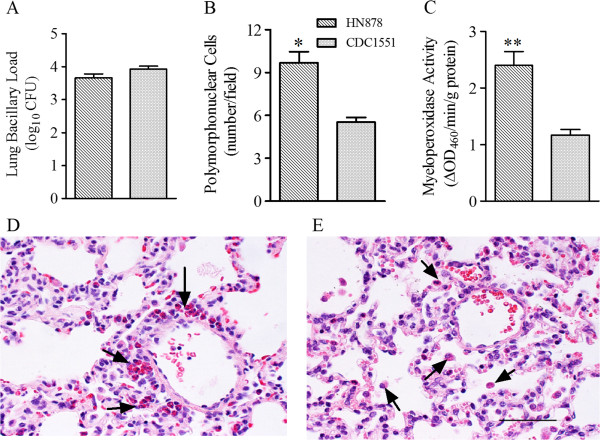
**Bacillary load, accumulation of activated PMN in Mtb-infected rabbit lungs. (A)** Total lung bacillary load in Mtb HN878- or CDC1551-infected rabbits at 3 hours post-infection. The values plotted are mean ± standard deviation from four animals per group **(B)** Numbers of polymorphonuclear (PMN) cells in the lungs of Mtb HN878- or CDC1551-infected rabbits at 3 hours post-infection. The values plotted are mean ± standard deviation **(C)**. Levels of myeloperoxidase (MPO) activity used to determine the activation status of PMNs. MPO activity was measured calorimetrically in the lung homogenates of Mtb HN878- or CDC1551-infected rabbits at 3 hours post-infection and reported as change in OD_460_ / min / g protein. The values plotted are mean ± standard deviation from triplicate assays from 3 animals per group. **(D** and **E)** Representative lung section histology of Mtb HN878- **(D)** or CDC1551- **(E)** infected rabbits at 3 hour post-infection stained with H&E and photographed at 400x magnification. Arrows point to PMNs. These cells in the rabbit contain red granules when stained with H&E and are known as heterophils. The scale bar (50 μM) is same for **(D)** and **(E)**.

To define the early response following pulmonary infection of rabbits with Mtb HN878 or CDC1551, we evaluated the bacillary load, by the CFU assay, and the immune cell accumulation, by histology of lung sections, at 3 hours post-infection (Figure 1). The bacillary load in the lungs of Mtb HN878- and CDC1551-infected rabbits was similar at this time point (Figure 1A). However, the H&E stained lung sections revealed an increased accumulation of leukocytes in the airspaces of lungs infected with HN878, relative to those infected with CDC1551, with significantly elevated numbers of PMN in the former group (Figure 1B, D and E). To confirm the morphological data, we measured the enzymatic activity of myeloperoxidase (MPO) in lung homogenates of rabbits infected with HN878 or CDC1551 as a surrogate for PMN activation [15]. Consistent with the histological findings, significantly higher MPO activity per gram of total protein was seen in the lungs of HN878- compared to CDC1551-infected rabbits (Figure 1C).

### Genome-wide transcriptional responses of Mtb-infected rabbit lungs at 3 hours

**Figure 2:**
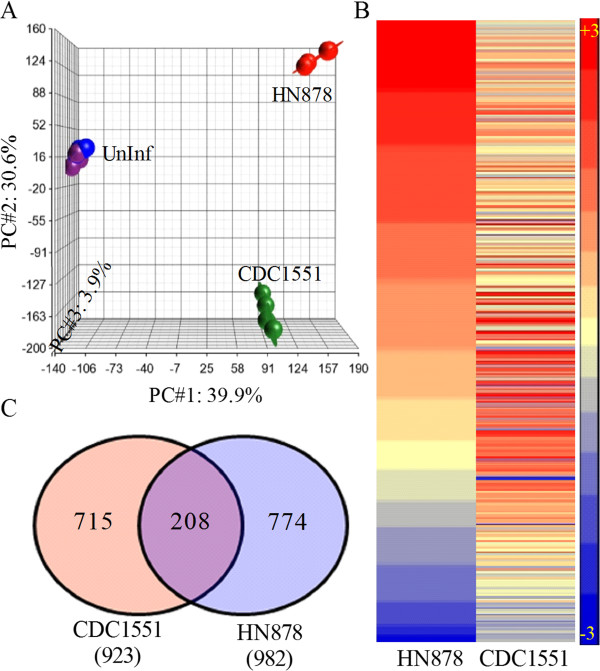
**Genome-wide rabbit lung transcriptome and profile of significantly differentially expressed genes (SDEG) at 3 hours post-Mtb-infection. (A)**. Principal component analysis (PCA) of microarray gene expression datasets from uninfected, Mtb HN878- or CDC1551-infected rabbit lungs at 3 hours. The elliptical line around each data point represents the standard deviation from the median. **(B)**. Intensity plot of SDEG in rabbit lungs following HN878 or CDC1551 infection at 3 hours. Gene expression values were sorted in a descending fashion for the HN878 dataset. The color scale ranges from +3 (red; upregulation) to −3 (blue; downregulation) **(C)**. Venn diagram showing the number of genes obtained from pair wise comparison between uninfected and Mtb HN878- or CDC1551-infected animals.

To evaluate the immune activation of lung cells in response to Mtb infection, we performed a genome-wide transcriptional analysis using total RNA isolated from HN878- or CDC1551-infected rabbit lungs at 3 hours (Figure 2). The quality of microarray data from the uninfected, HN878- or CDC1551-infected rabbit lungs was assessed using Principal Component Analysis (PCA) (Figure 2A). The three dimensional PCA plot shows 39.9% (x-axis; PC#1), 30.6% (y-axis; PC#2) and 3.9% (z-axis; PC#3) variation among biological replicates within each group and between different groups over time (infected versus uninfected). The PCA analysis also indicated that the individual datasets in each group cluster together and each cluster segregates from the other groups, indicating a reproducibility of variance (74.4%) among the components captured in the x-, y- and z-axis. To identify the significantly differentially expressed genes (SDEG), we used a cut-off family-wise error rate of 0.05 (0.05 FWER). A total of 490 SDEG were identified in the lungs of Mtb-infected, relative to uninfected, rabbits (Figure 2B). Infection with both HN878 and CDC1551 was associated with relatively high numbers of upregulated SDEG (342 versus 318 genes) and lower numbers of downregulated SDEG (172 versus 148 genes) (Figure 2B). The pair-wise analysis (i.e. expression ratio of Mtb-infected to uninfected rabbit lungs) revealed a moderately higher number of SDEG in the lungs of rabbits infected with HN878 (982), than in those infected with CDC1551 (923), with 208 genes shared between both groups (Figure 2C and Additional file 1: Table S2).

### Validation of microarray gene expression by real-time quantitative PCR (qRT-PCR)

A subset of the total SDEG was randomly selected for qRT-PCR to confirm and validate the microarray gene expression levels (Additional file 2: Table S3). The 14 selected rabbit genes included *TNF*, *IL4R*, *CD36*, *CXCL10*, *IL1A*, *CAV1*, *TGFB2*, *SPP1*, *CCL4*, *IL18*, *CCL2*, *IRF5*, *CD38* and *STAT1*. The qRT-PCR results for all the selected genes were qualitatively congruent with the data from the microarray analysis.

### Gene ontology and pathway analysis

The 13 top canonical pathways were identified from the SDEG with a 0.05FWER cut-off (p value 2x10^–8^), as described in the Methods (Table 1). The percentage of upregulated genes in each of the 13 pathways exceeded the downregulated genes (range: 61% to 100%) in the HN878-infected rabbit lungs. In contrast, only 10 of the pathways had more upregulated (range: 45% to 78%) than downregulated genes in the CDC1551-infected rabbit lungs. The remaining 3 pathways (chemokine receptors and chemokine, graft versus host disease and leishmania infection) had a higher number of downregulated genes (range: 51-55%) in the CDC1551-infected samples. In general, the total number of upregulated or downregulated SDEG differed between the two infection groups (Table 1).

**Table 1:** List of top canonical pathways affected by SDEG

**No.**	**Pathway**														***HN878***	***CDC1551***
		**1**	**2**	**3**	**4**	**5**	**6**	**7**	**8**	**9**	**10**	**11**	**12**	**13**	**Up / Down**	**Up / Down**
**1**	REACTOME_CHEMOKINE_RECEPTORS_BIND_CHEMOKINES **(41)**	***23***	22	0	0	6	0	0	1	22	0	23	0	0	17 / 6	11/12
**2**	KEGG_CHEMOKINE_SIGNALING_PATHWAY **(144)**	1	***54***	3	0	10	6	0	5	26	0	22	0	0	37 / 17	34 / 20
**3**	REACTOME_INTERFERON_GAMMA_SIGNALING **(40)**	0	0.1	***26***	5	3	5	4	0	0	0	0	5	1	21 / 5	15 / 11
**4**	KEGG_GRAFT_VERSUS_HOST_DISEASE **(21)**	0	0	0.3	***15***	2	8	3	0	3	0	0	14	3	15 / 0	7/8
**5**	KEGG_TOLL_LIKE_RECEPTOR_SIGNALING_PATHWAY **(80)**	0.3	0.3	0.1	0.1	***29***	7	0	0	8	2	6	2	2	27 / 2	17 / 12
**6**	KEGG_LEISHMANIA_INFECTION **(55)**	0	0.2	0.2	0.5	0.2	***29***	4	2	3	1	1	7	4	25 / 4	13 / 16
**7**	REACTOME_IMMUNOREGULATORY_INTERACTIONS_	0	0	0.2	0.2	0	0.2	***23***	6	0	2	1	3	3	21 / 2	17 / 6
	BETWEEN_A_LYMPHOID_AND_A_NON_LYMPHOID_CELL **(36)**															
**8**	REACTOME_CELL_SURFACE_INTERACTIONS_AT_THE_	0	0.1	0	0	0	0.1	0.3	***37***	2	6	2	0	3	23 / 14	27 / 10
	VASCULAR_WALL **(65)**															
**9**	KEGG_CYTOKINE_CYTOKINE_RECEPTOR_INTERACTION **(202)**	1	0.5	0	0.2	0.3	0.1	0	0.1	***66***	0	22	3	13	48 / 18	43 / 23
**10**	PID_INTEGRIN1_PATHWAY **(60)**	0	0	0	0	0.1	0	0.1	0.2	0	***36***	0	0	3	22 / 14	28 / 8
**11**	REACTOME_PEPTIDE_LIGAND_BINDING_RECEPTORS **(121)**	1	0.5	0	0	0.2	0	0	0.1	0.5	0	***44***	0	0	28 / 16	22 / 22
**12**	KEGG_ALLOGRAFT_REJECTION **(25)**	0	0	0.3	0.9	0.1	0.5	0.2	0	0.2	0	0	***15***	3	14 / 1	8/7
**13**	KEGG_HEMATOPOIETIC_CELL_LINEAGE **(68)**	0	0	0	0.2	0.1	0.1	0.1	0.1	0.4	0.1	0	0.2	***34***	24 / 10	25 / 9

The pathways are ranked 1 to 13 according to their p value and shown in **column 1** and **row 1**. The numbers in parenthesis after the pathway (column 2) refers to the total number of SDEG involved. This includes unique genes and those that are shared among different pathways. The numbers along the diagonal axis (underlined) are the number of SDEG involved in a specific pathway. Numbers shown above the diagonal axis are the number of SDEG shared between the pathways. The fractional numbers shown below the diagonal axis are the portion of SDEG shared by other pathways with reference to the numbers in the diagonal axis. The last two columns at the right show the number of up and down regulated genes in each pathway.

### Early induction of inflammatory response network in Mtb HN878 infected-rabbit lungs

**Figure 3:**
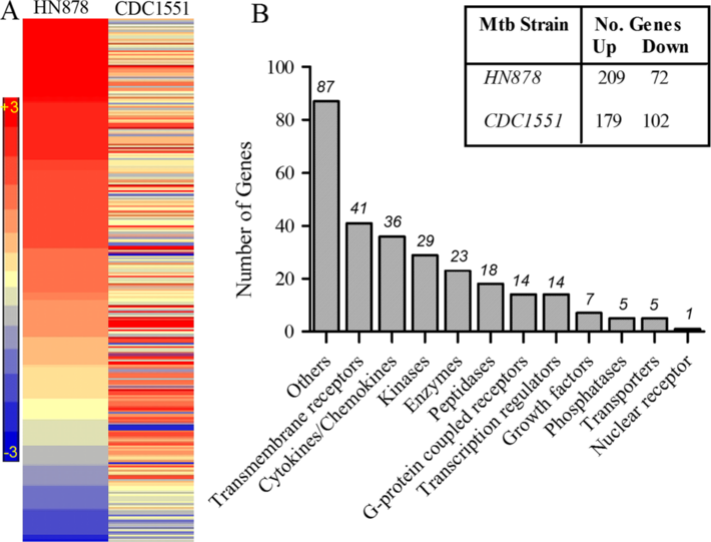
**Expression of inflammatory response network genes in Mtb-infected rabbit lungs. (A)**. Intensity plot of 281 SDEG involved in inflammatory response network in the HN878- or CDC1551-infected rabbit lungs at 3 hours. The color scale ranges from +3 (red; upregulation) to −3 (blue; downregulation) **(B)**. Functional classification of inflammatory response network genes. Numbers on top of each bar refer to subsets of genes in each functional category. The top right table shows the number of up and down regulated SDEG by Mtb HN878 or CDC1551 infection of rabbit lungs at 3 hours.

We interrogated the SDEG to identify the most significantly affected biological functions induced in response to Mtb infection compared to uninfected animals. As shown in Table 2, Ingenuity Pathway Analysis (IPA) of SDEG revealed inflammation and related pathological conditions as the most significantly affected biological functions. Of the 281 SDEG comprising the inflammatory response network, 209 were upregulated in response to HN878 infection, compared to 179 in CDC1551-infected rabbit lungs (Figure 3A and Additional file 3: Table S4). Gene ontology analysis revealed that the SDEG involved in the inflammatory response encode a variety of molecules including, cytokines, chemokines, surface receptors, enzymes, growth factors, transporters and transcriptional regulators that control the inflammatory response network (Figure 3B).

**Table 2:** Top biological functions affected by SDEG

**No.**	**Biological functions**	**p-Value**	**Molecules**
1	Inflammatory response	3.04E-72 - 1.69E-17	281
2	Inflammatory disease	9.02E-76 - 1.99E-17	228
3	Immunological disease	2.81E-61 - 2.22E-17	219
4	Skeletal and muscular disorders	9.02E-76 - 2.23E-17	195
5	Connective tissue disorders	9.02E-76 - 1.99E-17	180

The ranking for biological functions was based on the number of SDEG involved.

### Early activation of inflammatory response network by HN878 infection is localized to the lungs

To determine whether the early inflammatory response elicited by HN878 infection at 3 hours is localized to the lungs or whether it is systemic, we analyzed the expression of 12 selected SDEG, including cytokines and chemokines (*TNF*, *IL18*, *IL1A*, *SPP1*, *CCL2*, *CCL4*, *CXCL10*, *TGFB2*, *IL4R*, *CAV1*, *CD36* and *IRF5*), by qRT-PCR using total RNA from the blood leukocytes of HN878-infected rabbits at 3 hours, compared to uninfected animals (Additional file 4: Table S5). Interestingly, there was no statistically significant induction observed for any of the tested genes between uninfected and HN878-infected blood samples. This observation clearly suggests that the inflammatory response at 3 hours post-HN878 infection was localized to the lungs.

### Early regulation of STAT1 activation network in Mtb-infected rabbit lungs

**Figure 4:**
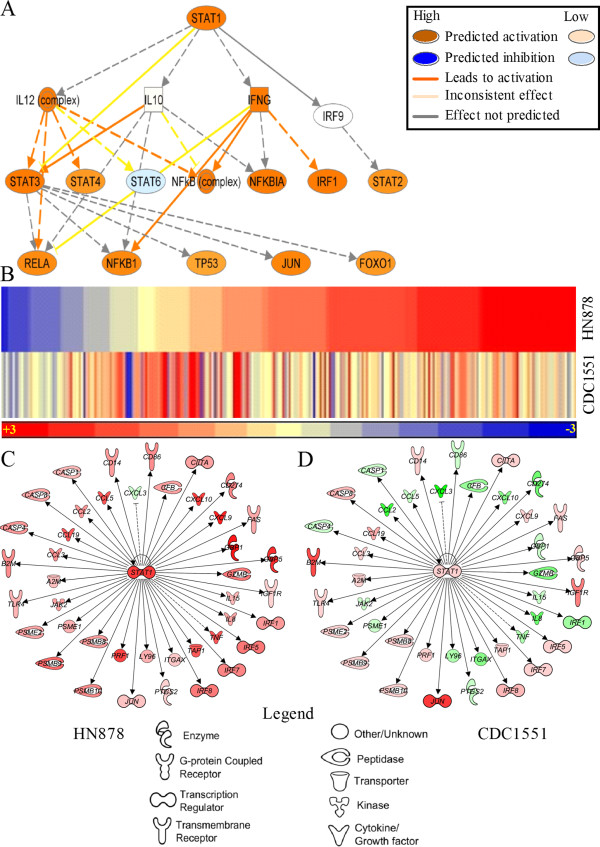
**Interaction and expression pattern of canonical STAT1 mechanistic pathway genes and STAT1 interaction network genes in Mtb-infected rabbit lungs. (A)** STAT1 regulation of downstream transcriptional regulators. Solid arrows indicate direct and broken arrow indicates indirect interactions. Predictions on the direction and intensity of activation and inhibition by STAT1 were made by IPA knowledgebase based on published literature. **(B)** Intensity plot of 260 SDEG involved in canonical STAT1 mechanistic pathway in HN878- or CDC1551-infected rabbit lungs at 3 hours. The color scale ranges from +3 (red; upregulation) to −3 (blue; downregulation). **(C** and **D)** Interaction of STAT1 network genes in HN878- **(C)** or CDC1551- **(D)** infected rabbit lungs at 3 hours. Solid lines indicate direct interactions and broken lines denote indirect interactions of genes. For **(C)** and **(D)**, gene symbols in red are up and green are down regulated. The color gradient of the gene symbols is proportional to their relative expression levels.

To understand how the early inflammation is regulated during Mtb infection of rabbit lungs, we analyzed the SDEG that encode transcription factors and studied their downstream networks. Of the 14 transcription regulators involved in the inflammatory response, nine (*STAT1*, *IRF5*, *IRF8*, *IRF7*, *IRF1*, *CIITA*, *JUN*, *NFKB1A*, *HMGB1*) had a significant z-score (≥ +2 indicates activation and ≤ −2 denotes inhibition of the downstream network) in the HN878-infected samples (Additional file 5: Table S6). Among these transcription factors, *STAT1* was the most highly upregulated (more than 7-fold) in rabbit lungs infected with HN878, compared to those infected with CDC1551. The canonical mechanistic pathway from IPA was used to identify plausible regulatory factors that are co-regulated by STAT1 to elicit the observed changes in the level of expression of target genes. As shown in Figure 4A, STAT1 interacts with 17 regulators, of which only 3 (*NFKB1A*, *IRF1* and *JUN*) were differentially expressed in both HN878- and CDC1551-infected rabbit lungs. *STAT1*, *IRF1* and *NFKB1A* were expressed at 14.2-, 5.4-, and 1.9-fold higher levels in HN878-infected lungs, relative to uninfected rabbit lungs. In contrast, expression of *JUN* was upregulated 3.6-fold in the CDC1551-infected lungs, compared to uninfected rabbit lungs. Next, we interrogated the SDEG to identify the target genes of the *STAT1* mechanistic network. Among the 261 SDEG involved in the STAT1 mechanistic network, the expression of 194 (74.3%) and 157 (60.1%) genes were upregulated in HN878- and CDC1551-infected rabbit lungs, respectively (Figure 4B and Additional file 6: Table S7). To decipher the activation status of the STAT1 network, we analyzed the direction of expression of *STAT1* interaction network genes. These genes are a subset of the 261 SDEG present in the mechanistic network. Interestingly, 41 out of 42 genes in this network were upregulated during HN878 infection, compared to only 22 in CDC1551-infected lungs (Figure 4C and D). Importantly, the direction of expression of these genes showed an early and robust activation of the STAT1 network in only the HN878- and not in the CDC1551-infected rabbit lungs at 3 hours. The expression pattern of all target genes (41 genes) of the STAT1 interaction network in the HN878-infected rabbit lungs is consistent with the IPA predicted activation of the STAT1 network, based on experimentally observed causal effect between the regulators and target genes (http://ingenuity.force.com/ipa/IPATutorials?id=kA250000000TNF7CAO).

### Gene expression in selected networks affected by Mtb infection of rabbit lungs

**Figure 5:**
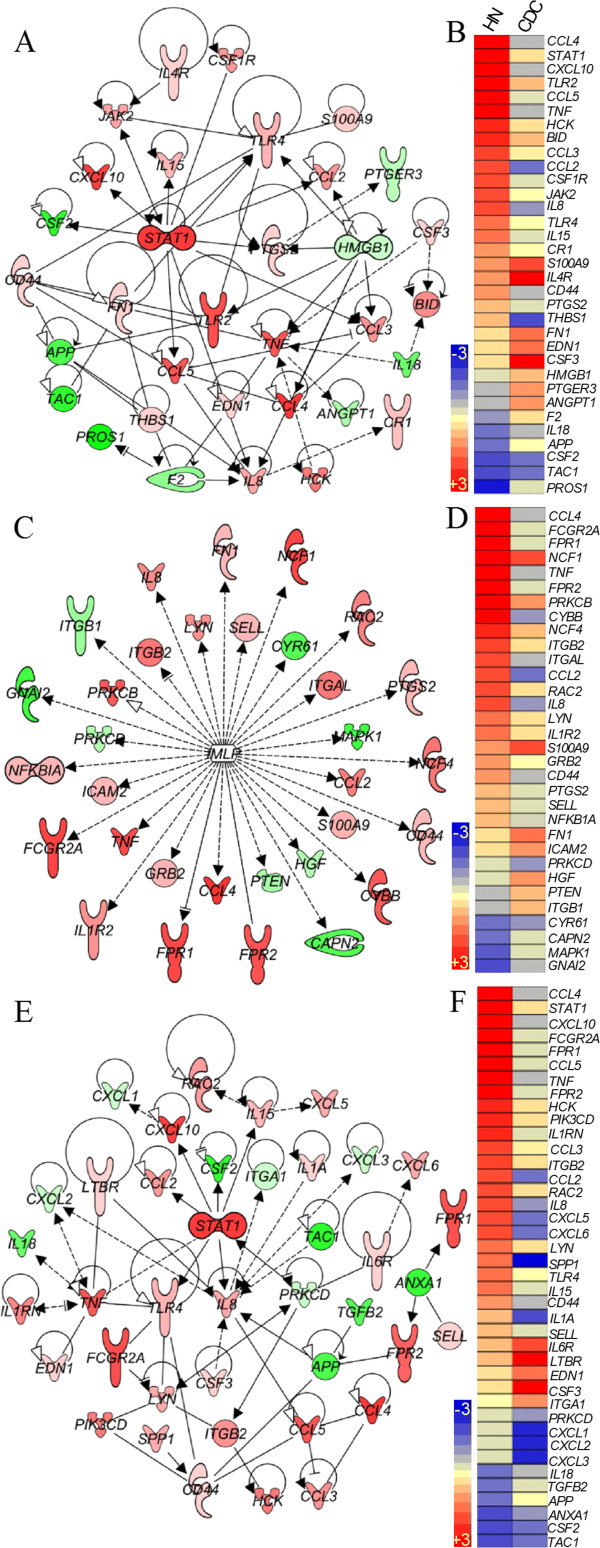
**Gene expression pattern and interaction in networks involved in macrophage activation (A and B), fMLP-stimulation (C and D) or PMN recruitment and activation (E and F) in the lungs of Mtb-infected rabbits at 3 hours. (A)** Interaction of macrophage activation network genes in the HN878-infected rabbit lungs. **(B)** Intensity plot of macrophage activation network genes in the Mb-infected rabbit lungs. **(C)** Interaction among genes involved in fMLP-stimulated network in the HN878-infected rabbit lungs. **(D)** Intensity plot of genes involved in fMLP-stimulated network in Mtb-infected rabbit lungs. **(E)** Interaction of PMN recruitment and activation network genes in the HN878-infected rabbit lungs. **(F)** Intensity plot of PMN recruitment and activation network genes in the Mb-infected rabbit lungs. The legend for the gene symbols in **(A)**, **(C)** and **(E)** is the same as in Figure 4. Gene symbols in red are up and green are down regulated. The color gradient of the gene symbols is proportional to their relative expression levels. Solid lines indicate direct and broken lines denote indirect interactions. For **(B)**, **(D)** and **(F)**, the gene expression values were sorted in a descending fashion for the HN878 dataset. The color scale in **(B)**, **(D)** and **(F)** ranges from +3 (red) to −3 (blue).

To better understand the causal link underlying the differential induction of the inflammatory response and STAT1 regulon networks, we studied gene networks involved in macrophage activation, fMLP-stimulation and recruitment and activation of PMN in infected rabbit lungs. The macrophage activation network contains a subset of 33 SDEG that encode cytokines and chemokines (*CCL4*, *CXCL10*, *CCL5*, *TNF*, *CCL3*, *CCL2*, *IL8*, *IL15*, *EDN1*, *CSF3*, *IL18* and *CSF2*), cell surface receptors (*TLR2*, *BID*, *CSF1R*, *TLR4*, *CR1*, *S100A9*, *IL4R*, *CD44*, *PTGER3* and *F2*), enzymes (*HCK*, *JAK2*, *PTGS2* and *FN1*) and transcriptional regulators (*STAT1* and *HMGB1*) (Figure 5A and B). At 3 hours post-infection, most of the macrophage activation network genes were upregulated in HN878-infected lungs, relative to those infected with CDC1551 (22 versus 14 genes). In contrast, a higher number of SDEG were downregulated in rabbit lungs following CDC1551 infection (15 versus 9 genes). Among the 14 upregulated genes in the CDC1551-infected animals relative to uninfected lungs, 9 genes (*ANGPT1*, *F2*, *PTGER3*, *HMGB1*, *EDN1*, *CSF3*, *FN1, S100A9* and *IL4R*) were expressed at much higher levels than those observed in the lungs of HN878-infected rabbits (Figure 5B).

fMLP is a chemoattractant peptide, produced by activated cells of the immune system, which stimulates recruited immune cells to produce proinflammatory molecules [16,17]. Similar to the macrophage activation network, genes induced by fMLP were differentially expressed in the rabbit lungs in response to HN878 versus CDC1551 infection (Figure 5C and D). Of the 32 genes in the fMLP-stimulated network, 24 and 13 genes were upregulated at 3 hours in the HN878- and CDC1551-infected animals, respectively. A higher number of genes were significantly downregulated in CDC1551-, than in HN878-infected lungs (18 versus 8). In this network, most of the genes that encode cytokines (*TNF* and *IL8*), chemokines (*CCL4* and *CCL2*), enzymes (*CYBB*, *CD44*, *NCF4*, *PTGS2*, *PRKCB* and *RAC2*) and receptors (*FCGR2A*, *FPR1*, *FPR2* and *IL1R2*) were upregulated only in the HN878-infected animals (Figure 5C). Six genes, *ITGB1*, *PTEN*, *HGF*, *ICAM2*, *FN1* and *S100A9,* were more upregulated in the CDC1551-infected rabbit lungs (Figure 5D).

Among the SDEG observed at 3 hours following Mtb infection, a subset of 40 genes are involved in the recruitment and activation of PMN. Of these, 29 were upregulated and 10 were downregulated in the HN878-infected rabbit lungs (Figure 5E and F). The majority of upregulated genes encode cytokines and chemokines, including *CCL4*, *CXCL10*, *CCL5*, *TNF* and *IL15*, as well as cell surface receptors, such as *FCGR2A*, *FPR1*, *FPR2* and *TLR4*, enzymes (*HCK*, *PIK3CD*, *SPP1*, *PRKCD* and others) and the transcriptional regulator, *STAT1* (Figure 5E). In contrast, CDC1551 infection was associated with upregulation of only 12 genes and downregulation of 26 genes in this pathway (Figure 5F).

Taken together, both the number of upregulated genes and the magnitude of their expression in the selected networks were generally higher in the lungs of HN878-infected animals. However, in the CDC1551-infected rabbits, upregulation of a subset of the genes belonging to these networks was noted.

### Interaction between inflammatory response, STAT1, macrophage and PMN activation networks

To identify key genes involved in the cellular processes driving the course of infection after implantation of HN878 or CDC1551 in rabbit lungs, we examined the number of genes shared among the host inflammatory response, STAT1 activation, PMN recruitment and activation, and macrophage activation networks (Additional file 7: Figure S1). Of the 281 SDEG involved in the host inflammatory response, 150 were also shared by the STAT1 regulation network. Moreover, all the genes involved in the PMN recruitment and activation and macrophage activation networks were part of the host inflammatory response network and many were also part of the STAT1 network (13 out of 40 genes in the PMN recruitment and activation and 20 out of 33 genes in the macrophage activation network). In addition, there were 17 genes commonly regulated by all four biological processes.

### The 4 week host response to infection with Mtb HN878 and CDC1551

**Figure 6:**
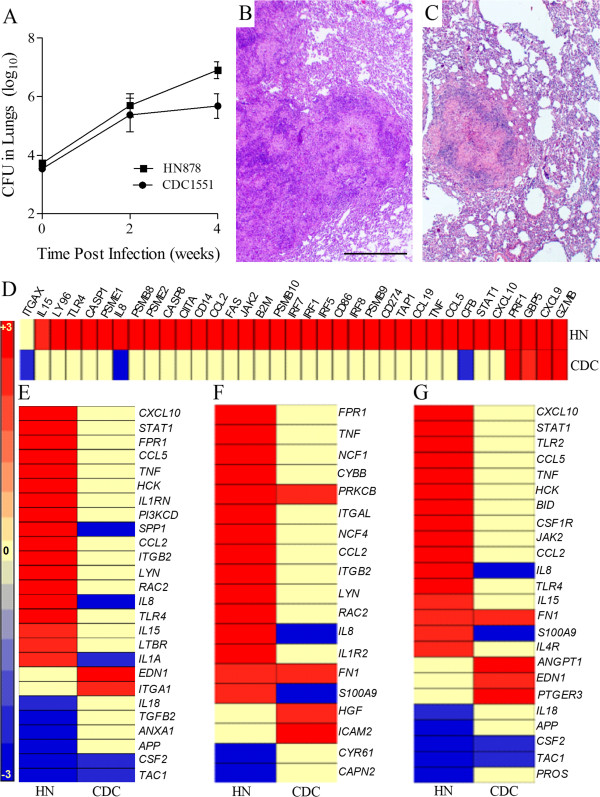
**Bacillary load, histology and expression pattern of selected network genes in Mtb-infected rabbit lungs at 4 weeks. (A)** Total lung bacillary load in Mtb HN878- or CDC1551-infected rabbits at 4 weeks post-infection. The values plotted are mean ± standard deviation for 3–5 animals per time point. **(B)** Hematoxylin and Eosin (H&E) stained lung sections of Mtb HN878-infected rabbits at 4 weeks post-infection. **(C)** H&E stained lung sections of Mtb CDC1551-infected rabbits at 4 weeks post-infection. The scale bar (1 mm) is same for **(B)** and **(C)**. **(D**-**G)** Intensity plots of STAT1 activation **(D)**, macrophage activation **(E)**, fMLP-stimulation **(F)** and PMN recruitment and activation network genes in the HN878- (HN) or CDC1551- (CDC) infected rabbit lungs at 4 weeks. For **(D**-**G)**, the gene expression values were sorted in a descending fashion for the HN878 dataset. The color scale ranges from +3 (red) to −3 (blue).

To validate our hypothesis that the outcome following Mtb infection is determined by the very early changes (3 hours) in the host immune response, we analyzed the bacillary load, histology and the previously selected network gene expression profiles in the lungs of HN878- or CDC1551-infected rabbits at 4 weeks (Figure 6). As shown in Figure 6A, rabbit lungs infected with similar numbers of HN878 and CDC1551 at 3 hours, multiplied similarly during the first 2 weeks, reaching 5.7 ± 0.7 and 5.4 ± 0.8 log_10_ CFU, respectively. Thereafter, the number of CFU in the lungs of CDC1551-infected rabbits stabilized, while HN878 continued to grow exponentially, reaching significantly higher numbers by 4 weeks. Histological examination of the lungs at 4 weeks showed striking differences in pathology between HN878- and CDC1551-infected rabbits (Figure 6B and C). Higher numbers of larger cellular granulomas were observed in the HN878-infected rabbit lungs compared to the CDC1551-infected animals. In addition, the cellular composition and distribution in the granulomas was different: in the HN878-infected rabbits, macrophages and lymphocytes were intermixed while in CDC1551-infected rabbits the granulomas were much more differentiated with a central area of macrophages and well demarcated lymphocytic cuffs.

We next analyzed the expression of the same network genes examined at 3 hours post-infection using a genome-wide transcriptome of rabbit lungs infected for 4 weeks. The selected networks included the host inflammatory response, STAT1 regulation, PMN activation, fMLP stimulation and macrophage activation (Figure 6D-G and Additional file 8: Figure S2). Of the 284 SDEG in the inflammatory network at 3 hours, a subset of 164 (134 up; 30 down) and 67 (53 up; 14 down) genes were also differentially expressed at 4 weeks in the lungs of HN878- or CDC1551-infected rabbits, respectively (Additional file 8: Figure S2). Moreover, 120 and 217 genes previously differentially expressed, were not significantly expressed at this time in HN878- or CDC1551-infected rabbit lungs, respectively. Thus, although the inflammatory response was generally dampened in both groups by 4 weeks of infection, similar to the observations at 3 hours, a much higher number of SDEG were upregulated in the HN878-infected animals, while the majority of these SDEG were not significantly expressed in the CDC1551-infected rabbits.

At 3 hours, we observed an upregulation of 42 of the 43 SDEG involved in the STAT1 interaction network in the lungs of HN878-, compared to only 23 in CDC1551-infected rabbit lungs (Figure 4C and D). Consistent with our findings at 3 hours, 32 of the 43 SDEG involved in the STAT1 interaction network were upregulated in the HN878-, compared to only 4 SDEG in the CDC1551-infected rabbit lungs (Figure 6D). Thus, similar to the inflammatory response network, the STAT1 interaction network shows a conservation of the gene expression pattern between 3 hours and 4 weeks. Relative to 3 hours, over 50% of the SDEG in each of the networks involved in the activation of macrophage, PMN and fMLP stimulation were differentially expressed at 4 weeks, with the majority upregulated only in the lungs of HN878-infected rabbits (Figure 6E-G). In contrast, only about 20% of SDEG in each of these networks were expressed in the CDC1551-infected rabbit lungs with the majority down regulated. Taken together, compared to 3 hours, the gene expression pattern shows a general dampening in the activation of PMN, macrophage and, fMLP stimulation networks at 4 weeks in both HN878- and CDC1551-infected rabbit lungs. However, while most of the genes in these networks remain upregulated in the HN878-infected rabbits, significant reductions in the number of genes and expression levels were noted in the CDC1551-infected animals.

## Discussion

**Figure 7:**
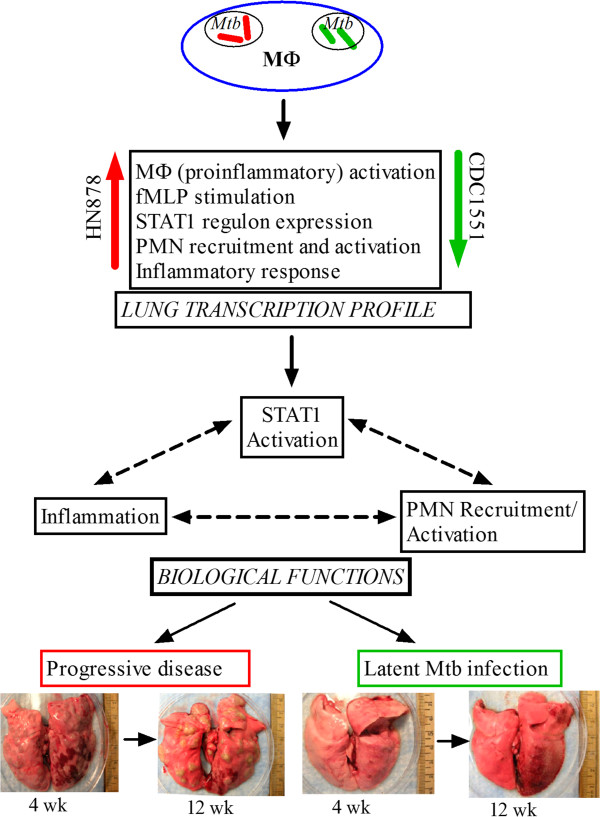
**Schematic illustration showing the interaction(s) among components of the early innate immune response at the transcriptional, cellular and organismal level during Mtb infection of rabbit lungs.** Transcription profile refers to the microarray gene expression data from HN878- or CDC1551-infected rabbit lungs. The arrows in red (HN878) denote unregulation and those in green (CDC1551) indicate downregulation of pathway genes. Pathogenesis refers to the progression of infection into active disease (HN878) or containment of infection and establishment of latency (CDC1551) in the rabbit lungs at 4 to 12 weeks post-infection.

Using two different Mtb clinical isolates, which give rise to progressive cavitary disease (HN878) versus spontaneous clearance of bacilli and establishment of LTBI (CDC1551) in rabbit lungs, we show that at similar lung bacillary burdens, a clear early (3 hours) difference in leukocyte recruitment and activation was noted. The differential leukocyte infiltration, including a significant difference in the accumulation of activated PMN, was associated with striking differences in the activation of gene networks involved in the host inflammatory response, STAT1 regulation and PMN recruitment, as well as in PMN and macrophage activation. Moreover, we confirmed our hypothesis that the early host immune response determines outcome following Mtb infection, by comparing the differential early response in the lungs to what is seen at 4 weeks of infection. Similar to 3 hours, we observed significantly increased induction of inflammatory responses, activation of STAT1, PMN and macrophages, and fMLP stimulation network gene expression profiles at 4 weeks in the lungs of HN878-infected animals, compared to CDC1551-infected rabbit lungs. Based on these findings, we suggest a model for the host response during early Mtb infection in the rabbit lungs that links specific patterns of macrophage activation in response to phagocytosis of the two Mtb strains, with differential activation of the STAT1-regulated inflammatory response (Figure 7). Accordingly, phagocytosis of HN878 by alveolar macrophages resulted in an early and robust expression of genes coding for pro-inflammatory molecules, including TNF-α, IL-8, IL-15, MCP-1 and CXCL10, that are associated with increased extravasation and activation of PMN in the lungs [18-20]. In contrast, CDC1551 infection, which failed to induce the expression of these genes, resulted in less recruitment and reduced activation of PMN.

The differential gene expression profile in response to infection with the two clinical Mtb isolates was noted as early as 3 hours. Clearly, the factors that initiate and regulate this differential response must have been activated even earlier. Some of the earliest mediators of inflammation induced in response to engaging macrophage receptors are the arachidonic acid (AA) metabolites (20:4), induced within minutes and shown to peak at 3 hours post LPS-stimulation of macrophages [21,22]. Aderem et al. showed that LPS-primed macrophages demonstrate enhanced production of 20:4 upon phagocytosis of zymosan, releasing AA into the extracellular milieu at one hour post-exposure [23]. Similarly, treatment of J774A.1 cells with AA or infection with mycobacteria induces NFkB activation and surface expression of CD69 within one hour; p38 MAP kinase activation in these cells is noted by 3 hours. Activation of NFkB and p38 MAP kinase is associated with increased actin polymerization, phagosome maturation and a TNF-α-mediated proinflammatory response [24,25]. Indeed, elevated levels of leukotrienes, a group of proinflammatory molecules derived from AA, have been found in the bronchoalveolar lavage (BAL) fluids from patients with active TB, and pleural macrophages isolated from the lung exudates of active TB patients show a significant increase in the LTB4 levels 4 hours after LPS stimulation [26]. Moreover, LTB4 contributes to the elevated chemotaxis of PMN from the circulation to the infected lungs [27,28]. Consistent with the results from these studies, we noted in the HN878-infected rabbit lungs a significant upregulation of *PTGS2* (also known as *COX2*), which encodes the prostaglandin synthase/cyclooxygenase enzyme involved in AA metabolism and acute inflammation [29]. Moreover, expression of *PTGER3*, an enzyme involved in prostaglandin metabolism, which mediates an anti-inflammatory response [30], was upregulated in CDC1551-infected lungs.

Increased recruitment of PMN to the site of infection is expected to exacerbate the local inflammatory response. For example, stimulation of human PMNs with LTB4 or fMLP, a proinflammatory chemoattractant produced by activated macrophages in response to Mtb and other agonists, leads to neutrophil activation, increased cell adhesion and improved phagocytic activity in these cells [31,32]. In the present study we found upregulation of genes that encode the fMLP receptors (*FPR1* and *FPR2*) in rabbit lungs as early as 3 hours after infection with HN878. This observation is consistent with the profound upregulation of macrophage and PMN activation network genes. Such activation of mature human blood neutrophils has been shown to be associated with an elevated transcription of *STAT1,* as well as increased phosphorylation of STAT1 protein [33]. Taken together, these observations support our interpretation of the gene expression patterns observed in the rabbit model of pulmonary TB. That is, phagocytosis of selected Mtb strains can be associated with early and robust macrophage activation, leading to a PMN-associated inflammatory response that will differentiate between phenotypically diverse Mtb strains. The differential macrophage response in the rabbit lungs is similar to the results from our *in vitro* infection studies using mouse bone marrow-derived macrophages infected with HN878 or CDC1551, where expression of inflammatory genes was significantly upregulated at 6 hours in response to HN878 infection while early immune activation network (EIAN) genes were upregulated in response to CDC1551 infection [34].

The role of PMN in the control of Mtb infection and the pathogenesis of TB is not clearly understood. This is in part due to the short life span of PMN (less than 24 hours) and to the dogma that macrophages, and not PMNs, are the primary habitat of infecting bacilli during chronic, pulmonary TB. More recently, studies using PMN-depleted mice highlighted the importance of these cells in the host response to Mtb infection [35-37]. In mice, antibody-mediated neutralization of PMN exacerbated bacillary growth in the lung, spleen and liver [35]. In contrast, Mtb infection of genetically susceptible mice has been shown to be associated with an increased expression of genes involved in inflammation and neutrophil recruitment in the lungs. In these animals, increased PMN recruitment to the peritoneal cavity was noted as early as 60 minutes post-infection, resulting in neutrophilia associated with compromise of the protective Th1 type immune response [38,39]. In our study, infection with an Mtb strain that is not controlled was associated with early accumulation (3 hours) of higher numbers of PMN in the lungs of infected rabbits. Thus, although our results implicate PMN in the progression to active disease, it is not clear whether they drive the differential progression of infection or whether they are merely associated with differential macrophage responses. Recently, Berry et al. reported increased levels of interferon-inducible gene transcripts, originated from myeloid cells, including PMN, in the blood of active TB patients, relative to those in latently infected individuals [40]. Taken together, these observations support our conclusion that during Mtb infection, increased inflammation with recruitment and activation of PMN is associated with progression to active disease rather than control of infection.

Clearly, very early events induced by the interactions between the phagocyte and the pathogen can result in radically different outcomes, suggesting that the initial profile of macrophage differentiation will determine the nature of both innate and acquired immune responses [34,41,42]. The range of phagocyte differentiation induced by various Mtb strains is a manifestation of the plasticity of the cells and their ability to sense and respond to different microbial agonists and mediators of host immunity [43,44]. However, exactly how the early inflammatory response subverts the development of a protective immune response is not fully understood. It has been shown that TNF-α is important for the organization and maintenance of granulomas and the associated host response in animal models of Mtb infection [45-47]. In this study, we observed increased *TNFA* and *CCL2* levels in the lungs of rabbits infected with HN878, relative to CDC1551, at 3 hours. However, previous studies in human and mouse monocytes/macrophages, as well as in mice, have shown that, compared to HN878, infection with CDC1551 induces higher production of inflammatory molecules, including TNF-α and CCL-2 [14,48,49]. This discordance is most likely due to the differential kinetics of macrophage activation in vivo and invitro as well as inherent differences between the rabbit and mouse models. In the present study, transcript levels in rabbit lungs were measured at 3 hours post-infection, whereas protein and transcript levels of TNF-α and CCL-2 were determined at 7, 14, 21, 28 and 60 days in infected mice or 24, 48, 72 and 96 hours in Mtb-infected human PBMC, in other published reports [14,48,49]. Importantly, increased levels of TNF-α have been documented in the blood and pleural fluids of active TB patients, compared to healthy contacts (latent TB) [50,51]. Moreover, a positive correlation has been observed between increased TNF-α levels and the severity of clinical disease in active TB patients [51].

In our study, we noted a general dampening of differentially regulated host immune/inflammatory response network genes in the lungs of HN878- and CDC1551-infected rabbits at 4 weeks, compared to 3 hours. However, the direction and pattern of expression of most of the genes in the inflammation related innate immune response networks were conserved between 3 hours and 4 weeks. In contrast to HN878 infection, the majority of these network genes was not expressed or was down regulated in the CDC1551-infected rabbit lungs at both time points. This suggests that the early onset of inflammation associated innate immune activation in the HN878-infected rabbit lungs leads to exacerbated lung pathology and bacterial growth. In contrast, dampened inflammatory networks as early as 3 hours alleviates disease progression and facilitates control of infection in the CDC1551-infected rabbit lungs. These early changes in the regulation of host immune response, including recruitment of neutrophils, drive subsequent cellular events that culminate in the differential outcome of infection between HN878 and CDC1551 in rabbits. Consistently, compared to CDC1551, HN878-infected rabbit lungs show progressively increasing inflammation, suboptimal activation of macrophages and compromised protective Th1 responses from 4 to 12 weeks post-infection, at which time the animals have established chronic cavitary disease [12,13]. Our results are supported by recent studies that showed a prominent inflammation-associated neutrophil transcript profile specifically in the peripheral blood of active TB patients, compared to individuals with LTBI. Expression of these biomarkers of active TB was abrogated after successful antibiotic treatment and alleviation of clinical disease [40].

In the present study, the increased recruitment of immune cells, including PMNs, likely contributed to the elevated transcript levels of SDEG that we observed at 3 hours in the HN878-infected rabbit lungs. To fully understand how early regulation of inflammation is associated with the outcome of Mtb infection a detailed kinetic analysis of host immunity is required. By directly comparing the evolution of the immune response in the lungs of rabbits infected with HN878 versus CDC1551, we can identify the immunological determinants of protection over the course of infection. Such comparative studies will enable us to identify biomarkers that most efficiently discriminate between establishment of active disease and LTBI for use in predicting the outcome of infection. Since biomarkers of response to infection and treatment in humans need to be detectable in peripheral blood, future studies in our rabbit model will require identification of appropriate biomarkers in the circulation that can discriminate different stages of lung infection and/or disease.

## Conclusions

In this study, we describe the early (3 hours post-infection) and more chronic (4 weeks) rabbit lung immune response to infection with two clinical isolates of Mtb that yield differential outcome over time. Based on our observations, we propose a model where immune activation as demonstrated by gene expression changes in the lungs, as early as 3 hours post-infection, and associated differential recruitment and activation of inflammation-associated innate immune cells, such as PMN and macrophages, significantly influences the overall outcome of Mtb infection in rabbits at later time points.

## Methods

### Ethics statement

All rabbit procedures were performed in accordance with Animal Welfare Act guidelines and approved by the Institutional Animal Care and Use and Institutional Biosafety Committees of UMDNJ.

### Mycobacteria for infection

*Mycobacterium tuberculosis* (Mtb) HN878 and CDC1551 were grown in Middlebrook 7H9 (BD, Sparks, MD); inoculum for rabbit infections were prepared, as described [52].

### Aerosol infection of rabbits

Female New Zealand White rabbits (~2.5 kg; Millbrook Farms, MA, USA) were exposed to HN878 or CDC1551 aerosols, as described [12]. Uninfected rabbits served as controls. At 3 hrs and 4 weeks post-infection, rabbits were sedated with intramuscular administration of Ketamine plus Xylazine and euthanized by intravenous injection of Euthasol. Lung and blood samples were collected for gene expression analysis.

### Enumeration of lung bacillary load

Portions of lung lobes (about 30% of the entire lung) were homogenized in saline, serially diluted and plated on 7H11-agar (BD, Sparks, MD), as described [52]. Plates were incubated at 37°C for 4 to 5 weeks; bacterial CFU were counted and calculated for the entire lung. Detection limit of this assay was < 25 CFU.

### Rabbit lung histology

Five-micron sections of formalin-fixed, paraffin-embedded lung tissues from Mtb-infected rabbits were stained with hematoxylin and eosin (H&E). Leukocytes were enumerated microscopically at 40x magnification. Four independent counts per animal, each of 4 random fields, (four CDC1551- and seven HN878-infected rabbits) were used for calculations.

### Measurement of myeloperoxidase (MPO) activity

MPO activity was determined calorimetrically in the lung homogenates, as described [15]. Color development was read at 460 nm (OD_460_) at one-minute intervals for 10 minutes and MPO activity was expressed as total MPO activity/minute/gram of protein. Total protein was estimated using BCA Kit (Thermo Fisher Scientific, Rockford, IL).

### Isolation of total RNA from rabbit lungs

Portions of tissue were homogenized in TRIzol (Invitrogen, CA, USA), extracted with bromo-chloropropane, and supernatants were processed using NucleoSpin kit as per instructions (Macherey-Nagel, GmbH) to prepare total RNA, as described [52]. RNA quantity/quality was estimated by NanoDrop (NanoDrop Products, DE).

### Microarray analysis of rabbit gene expression

Total lung RNA from each uninfected or Mtb-infected rabbit was used for cDNA synthesis, as described [52]. For each infected class, cDNA from 4 infected animals was hybridized separately with a single pool of cDNA from 4 uninfected animals using a two-color rabbit microarray (Agilent Technologies, Santa Clara, CA) following the manufacturer’s procedures. The expression data sets for the 43,803 probes were collected from two sets of experiments, HN878 versus uninfected and CDC1551 versus uninfected. The data was background-corrected and normalized using Bioconductor software [53]. The microarray data is submitted to Gene Expression Omnibus (accession number: GSE49947).

### Statistical analysis of microarray data

Microarray data were split into 3 classes: HN878-infected, CDC551-infected, uninfected and used in a one-way ANOVA test of the null hypothesis of equal mean of log-transformed intensities among the 3 classes. ANOVA was performed with variance stabilization to yield F-statistics/p-values overall and for the 3 pair-wise comparisons (lmFit, contrasts.fit, eBayes from Bioconductor limma package). Permutation tests established with a 0.05 family-wise error rate (0.05 FWER) was used to identify transcriptome-wide significantly differentially expressed genes (SDEG). See Additional file 9 section for more details on methods.

### Pathway enrichment analysis

Canonical pathways were obtained from Molecular Signatures database (MolSigDB) and restricted to genes present on the microarray; 961 pathways containing from 15 to 500 genes were retained [54]. Primary sources of pathways were Reactome (437 pathways), Pathway Interaction Database (PID) (176 pathways), Kyoto Encyclopedia of Genes and Genomes (KEGG) (167 pathways), and Biocarta (114 pathways) [55-57]. For each pathway, the p-value for a null hypothesis of equal differential expression weight was calculated using a one-sided, equal-variance t-test, comparing weights for genes in the pathway to weights for the remaining genes. Pathways biased towards small p-values were removed from analysis; the 0.05 FWER thresholds for pathway enrichment was 2x10^–8^.

### Gene interaction network analysis

The SDEG were loaded to Ingenuity Pathway Analysis (IPA) software (Ingenuity Systems, Redwood City, CA) for functional characterization, as described [52]. We used the IPA knowledgebase to interrogate top biological functions, gene interaction networks, and upstream regulatory factors affected by SDEG. IPA uses a regulation z-score algorithm to predict the activation/inhibition state of transcriptional regulators and associated networks, where a z-score of ≥ +2 or ≥ −2 predicts activation or inhibition, respectively.

### Quantitative real time pcr analysis (qRT-PCR)

qRT-PCR was performed using total RNA, as described [52]. Rabbit gene primers are listed in Additional file 10: Table S1. The threshold cycle (Ct) for each amplified target was calculated using MxPro software. The house-keeping gene *GAPDH* was used for normalization. Fold-change in gene expression was calculated by 2^-ΔΔCt^ (where ^ΔCt^ is the difference in Ct between target gene and *GAPDH*). Experiments were repeated at least 3 times with RNA from 2–4 animals per group.

## Competing interests

The authors declare that they have no competing interests.

## Authors’ contributions

SS and GK conceived the idea and designed the experiments. SS, LT, POB, VK, NLK and BP performed the experiments. SS, NB, PS, JSB and PCK contributed to the microarray data analysis and to improve the rabbit gene annotation. SS, DF and GK drafted the manuscript. All authors read and approved the final manuscript.

## Supplementary Material

Additional file 1: Table S2 Level of expression and p-value significance of SDEG in the lungs of Mtb-infected rabbits at 3 hours. Click here for file

Additional file 2: Table S3 Validation of microarray gene expression in the lungs of Mtb-infected rabbits at 3 hours by qRT-PCR. Click here for file

Additional file 3: Table S4 List of SDEG involved in inflammatory response in Mtb-infected rabbit lungs at 3 hours. Click here for file

Additional file 4: Table S5 qRT-PCR analysis of gene expression in the blood of uninfected and HN878-infected rabbits at 3 hours. Click here for file

Additional file 5: Table S6 List of top transcription regulator genes differentially expressed in the lungs of Mtb-infected rabbits at 3 hours. Click here for file

Additional file 6: Table S7 List of SDEG involved in the canonical STAT1 mechanistic network in the lungs of Mtb-infected rabbits at 3 hours. Click here for file

Additional file 7: Figure S1 Venn diagram showing distribution of the SDEG among the selected networks in the lungs of Mtb-infected rabbits at 3 hours. Click here for file

Additional file 8: Figure S2 Expression of inflammatory response and STAT1 activation network genes in Mtb-HN878- or CDC1551 infected-rabbit lungs at 3 hours and 4 weeks. Click here for file

Additional file 9 **Methods.** Updated annotation of rabbit gene probes for microarray and Statistical analysis of microarray data. Click here for file

Additional file 10: Table S1 List of oligonucleotide primers used for qRT-PCR experiments. Click here for file
