# Supplementary material for: Early innate immunity determines outcome of Mycobacterium tuberculosis pulmonary infection in rabbits
Source: Cell Commun Signal. 2013 Aug 19;11:60. doi: 10.1186/1478-811X-11-60 (PMC3765177; doi:10.1186/1478-811X-11-60)
Supplement: Additional file 1: Table S2 — Level of expression and p-value significance of SDEG in the lungs of Mtb-infected rabbits at 3 hours. [file 1478-811X-11-60-S1.doc]

**Supplementary Table S2**: Level of expression and p-value significance of SDEG in the lungs of Mtb-infected rabbits at three hours

| **Genes** | **F.p.value**  **(HN-Uninf)** | **log Fold Change**  **(HN-UnInf)** | **Genes** | **F.p.value**  **(CDC-Uninf)** | **log Fold Change**  **(CDC-UnInf)** |
| --- | --- | --- | --- | --- | --- |
| *ITLN1* | 5.15E-10 | -3.98 | *SPP1* | 1.55E-09 | -3.28 |
| *GPM6A* | 5.94E-10 | -3.38 | *SLC6A14* | 7.88E-08 | -2.19 |
| *CLIC5* | 4.71E-10 | -3.28 | *CXCL1* | 1.41E-08 | -2.13 |
| *DEFA3* | 1.26E-10 | -3.24 | *CXCL2* | 1.41E-08 | -2.13 |
| *DEFA1* | 1.26E-10 | -3.24 | *CXCL3* | 1.41E-08 | -2.13 |
| *DEFA1B* | 1.26E-10 | -3.24 | *CHI3L2* | 1.04E-07 | -2.11 |
| *DEFA4* | 1.26E-10 | -3.24 | *CCL20* | 5.09E-09 | -2.05 |
| *CYP2B6* | 2.11E-09 | -3.05 | *ALDH1A2* | 9.75E-07 | -2.05 |
| *CCDC152* | 2.40E-09 | -2.74 | *IL1A* | 5.90E-07 | -2.00 |
| *PPM1L* | 2.29E-08 | -2.73 | *DNAH11* | 1.85E-08 | -1.89 |
| *CLDN18* | 3.09E-08 | -2.61 | *SLC26A4* | 2.43E-06 | -1.88 |
| *FAM25E* | 2.01E-06 | -2.60 | *LCN2* | 8.03E-08 | -1.83 |
| *FAM25G* | 2.01E-06 | -2.60 | *PPM1L* | 5.78E-07 | -1.80 |
| *FAM25A* | 2.01E-06 | -2.60 | *GPRC5A* | 9.98E-07 | -1.79 |
| *FAM25B* | 2.01E-06 | -2.60 | *CFI* | 3.12E-07 | -1.75 |
| *FAM25C* | 2.01E-06 | -2.60 | *WDR52* | 1.53E-06 | -1.73 |
| *EFEMP1* | 6.59E-08 | -2.60 | *C14orf105* | 1.83E-06 | -1.73 |
| *SLC15A2* | 1.19E-09 | -2.59 | *EFEMP1* | 1.54E-06 | -1.72 |
| *GPRC5A* | 6.53E-08 | -2.55 | *KMO* | 7.20E-07 | -1.70 |
| *DTWD2* | 1.76E-06 | -2.54 | *SPINK2* | 3.47E-07 | -1.68 |
| *CCDC3* | 9.49E-10 | -2.53 | *DNAH7* | 4.67E-07 | -1.66 |
| *TMED6* | 4.23E-08 | -2.53 | *NEK10* | 6.58E-07 | -1.64 |
| *C5* | 5.96E-09 | -2.53 | *EFCAB1* | 3.91E-07 | -1.63 |
| *PROS1* | 4.29E-08 | -2.50 | *C12orf55* | 1.87E-06 | -1.62 |
| *PLA2R1* | 2.66E-08 | -2.45 | *GPM6A* | 1.84E-07 | -1.62 |
| *PGRMC1* | 8.73E-09 | -2.43 | *TEX26* | 8.55E-07 | -1.58 |
| *NDNF* | 1.68E-09 | -2.41 | *KCNE1* | 6.06E-07 | -1.57 |
| *PHACTR2* | 4.61E-08 | -2.39 | *WDR63* | 3.18E-07 | -1.56 |
| *KDM4A* | 4.61E-08 | -2.39 | *C11orf88* | 3.98E-07 | -1.55 |
| *SPAG16* | 1.03E-06 | -2.38 | *KRT8* | 1.08E-06 | -1.54 |
| *ALDH1A2* | 3.07E-07 | -2.38 | *AGR2* | 2.08E-06 | -1.53 |
| *HTR3B* | 1.96E-06 | -2.30 | *CCDC113* | 2.87E-07 | -1.52 |
| *SEC14L3* | 7.23E-07 | -2.29 | *TAC1* | 1.43E-07 | -1.52 |
| *ANXA8L1* | 1.85E-08 | -2.29 | *HSPA1A* | 1.17E-06 | -1.52 |
| *ANXA8* | 1.85E-08 | -2.29 | *HSPA1B* | 1.17E-06 | -1.52 |
| *ANXA8L2* | 1.85E-08 | -2.29 | *CXCL5* | 1.71E-06 | -1.51 |
| *ANXA3* | 2.18E-08 | -2.27 | *CXCL6* | 1.71E-06 | -1.51 |
| *SEMA3B* | 2.24E-06 | -2.27 | *GPAM* | 8.89E-07 | -1.48 |
| *SCEL* | 1.18E-07 | -2.27 | *C10orf35* | 8.04E-08 | -1.48 |
| *TMEM212* | 6.81E-08 | -2.25 | *PLA2G2A* | 2.56E-07 | -1.48 |
| *LAMA3* | 3.48E-08 | -2.25 | *ATF3* | 4.63E-07 | -1.48 |
| *FGG* | 1.99E-08 | -2.25 | *C4orf47* | 2.52E-06 | -1.47 |
| *RTKN2* | 3.08E-08 | -2.22 | *UGP2* | 5.78E-07 | -1.47 |
| *C14orf37* | 5.73E-09 | -2.21 | *C6orf165* | 8.05E-08 | -1.47 |
| *CAV1* | 1.33E-08 | -2.21 | *SPAG17* | 1.42E-06 | -1.47 |
| *MAP2* | 2.04E-08 | -2.19 | *ATP13A5* | 1.37E-07 | -1.46 |
| *SGCE* | 4.46E-07 | -2.19 | *SPAG6* | 2.49E-07 | -1.46 |
| *GHR* | 1.59E-08 | -2.17 | *TTC6* | 2.02E-06 | -1.46 |
| *NICN1* | 4.87E-09 | -2.17 | *KRTCAP3* | 1.70E-06 | -1.45 |
| *GSTA1* | 1.51E-08 | -2.16 | *CSF2* | 2.92E-07 | -1.44 |
| *GSTA2* | 1.51E-08 | -2.16 | *CCDC173* | 1.61E-06 | -1.44 |
| *GSTA3* | 1.51E-08 | -2.16 | *LAMP3* | 1.76E-07 | -1.43 |
| *GSTA5* | 1.51E-08 | -2.16 | *FABP3* | 1.18E-06 | -1.42 |
| *CES1* | 2.30E-08 | -2.15 | *HBB* | 9.80E-07 | -1.41 |
| *METTL7A* | 4.15E-08 | -2.15 | *HBD* | 9.80E-07 | -1.41 |
| *PENK* | 2.89E-06 | -2.14 | *FGF1* | 3.93E-07 | -1.39 |
| *GCLC* | 1.49E-07 | -2.13 | *STOX1* | 2.18E-06 | -1.39 |
| *NOX4* | 2.51E-07 | -2.12 | *TEX12* | 1.59E-06 | -1.39 |
| *FBXO4* | 8.69E-09 | -2.12 | *PLA2R1* | 2.11E-06 | -1.39 |
| *MYL12B* | 3.84E-08 | -2.12 | *SULT1A3* | 2.15E-07 | -1.38 |
| *FGF1* | 1.60E-08 | -2.11 | *SULT1A2* | 2.15E-07 | -1.38 |
| *C1orf116* | 2.51E-06 | -2.11 | *SULT1A4* | 2.15E-07 | -1.38 |
| *PTPLAD2* | 1.56E-08 | -2.11 | *SULT1A1* | 2.15E-07 | -1.38 |
| *ZDHHC2* | 4.43E-09 | -2.10 | *SOD2* | 6.98E-07 | -1.37 |
| *UCHL1* | 5.03E-08 | -2.09 | *INSIG1* | 3.87E-07 | -1.37 |
| *LEPREL1* | 6.65E-07 | -2.08 | *AKAP14* | 4.87E-07 | -1.36 |
| *ZDHHC5* | 1.94E-08 | -2.07 | *CCDC110* | 1.46E-06 | -1.35 |
| *CCRL1* | 4.42E-08 | -2.06 | *SDCBP2* | 6.25E-07 | -1.35 |
| *SLC6A14* | 1.30E-07 | -2.05 | *TMEM56* | 1.61E-07 | -1.35 |
| *LMOD2* | 1.54E-06 | -2.03 | *ASB14* | 3.15E-07 | -1.34 |
| *LURAP1L* | 8.68E-09 | -2.03 | *CCDC153* | 7.85E-08 | -1.34 |
| *LIPH* | 1.23E-06 | -2.03 | *FANK1* | 8.44E-07 | -1.34 |
| *DNAH11* | 1.09E-08 | -2.02 | *CXADR* | 1.45E-07 | -1.32 |
| *CD9* | 7.30E-08 | -2.00 | *ST3GAL5* | 2.26E-06 | -1.32 |
| *KRT8* | 1.52E-07 | -1.99 | *RTKN2* | 1.86E-06 | -1.30 |
| *SRPX* | 4.40E-08 | -1.97 | *FAM216B* | 3.66E-07 | -1.30 |
| *ATP5S* | 2.93E-07 | -1.97 | *PIGR* | 2.91E-07 | -1.30 |
| *TAC1* | 1.93E-08 | -1.97 | *EFCAB10* | 1.07E-06 | -1.29 |
| *CREBL2* | 1.75E-08 | -1.96 | *RGS7BP* | 9.68E-07 | -1.28 |
| *TEX12* | 1.16E-07 | -1.95 | *SCD* | 1.27E-07 | -1.28 |
| *TEAD1* | 9.10E-07 | -1.95 | *SHMT1* | 1.11E-06 | -1.27 |
| *SCP2* | 3.58E-08 | -1.94 | *C5* | 1.32E-06 | -1.26 |
| *SPINK2* | 1.12E-07 | -1.94 | *GLRX* | 4.48E-07 | -1.26 |
| *TMEM56* | 9.50E-09 | -1.94 | *EVA1C* | 2.17E-06 | -1.24 |
| *F3* | 1.06E-08 | -1.93 | *EPCAM* | 2.36E-06 | -1.24 |
| *FLRT3* | 7.52E-09 | -1.92 | *PIH1D3* | 1.61E-06 | -1.24 |
| *KCNE1* | 1.27E-07 | -1.92 | *VEGFB* | 2.10E-06 | -1.24 |
| *CSF2* | 3.15E-08 | -1.92 | *CAPSL* | 1.80E-06 | -1.22 |
| *KIF21A* | 2.58E-08 | -1.91 | *KIF21A* | 7.96E-07 | -1.22 |
| *UTRN* | 3.92E-08 | -1.91 | *MORN2* | 2.35E-07 | -1.22 |
| *RAVER2* | 1.81E-08 | -1.90 | *PERP* | 2.29E-07 | -1.22 |
| *JAK1* | 1.81E-08 | -1.90 | *HSPH1* | 6.42E-07 | -1.22 |
| *FLRT1* | 2.53E-08 | -1.90 | *PON1* | 7.02E-07 | -1.21 |
| *FLRT2* | 2.53E-08 | -1.90 | *ZDHHC2* | 3.22E-07 | -1.21 |
| *FBXW12* | 2.20E-06 | -1.90 | *SLC15A2* | 4.46E-07 | -1.21 |
| *KLF5* | 4.96E-07 | -1.88 | *CYP2B6* | 2.73E-06 | -1.21 |
| *FGF13* | 3.47E-08 | -1.88 | *PGRMC1* | 2.37E-06 | -1.17 |
| *PERP* | 8.46E-09 | -1.86 | *EHF* | 2.15E-06 | -1.16 |
| *AKAP12* | 9.24E-08 | -1.84 | *CLEC7A* | 1.48E-06 | -1.15 |
| *HOPX* | 1.06E-08 | -1.84 | *PPIL6* | 2.26E-06 | -1.14 |
| *MAMDC2* | 2.47E-06 | -1.83 | *CLIC5* | 1.77E-06 | -1.14 |
| *MME* | 2.59E-07 | -1.83 | *ENKUR* | 6.88E-07 | -1.13 |
| *BCL2L2* | 7.31E-07 | -1.81 | *WDR66* | 2.43E-06 | -1.13 |
| *YAP1* | 7.31E-07 | -1.81 | *FAM183A* | 9.07E-07 | -1.12 |
| *TPMT* | 2.72E-06 | -1.81 | *FAM183B* | 9.07E-07 | -1.12 |
| *EPCAM* | 1.38E-07 | -1.80 | *RAB11FIP1* | 2.10E-06 | -1.12 |
| *ABCA5* | 2.20E-07 | -1.80 | *SLC7A7* | 5.47E-07 | -1.11 |
| *ANXA1* | 2.00E-08 | -1.80 | *TC2N* | 1.06E-06 | -1.10 |
| *PDZRN4* | 2.70E-06 | -1.79 | *DNAI1* | 8.26E-07 | -1.10 |
| *TMEM47* | 1.85E-07 | -1.78 | *ME1* | 1.79E-06 | -1.09 |
| *EN1* | 1.85E-07 | -1.78 | *WDR96* | 2.08E-06 | -1.07 |
| *NPC1* | 5.43E-08 | -1.78 | *ORM1* | 1.87E-06 | -1.07 |
| *CYB5A* | 5.66E-08 | -1.78 | *ORM2* | 1.87E-06 | -1.07 |
| *ARHGAP28* | 1.90E-07 | -1.77 | *TRPM6* | 1.87E-06 | -1.07 |
| *HEBP2* | 9.83E-08 | -1.77 | *NDNF* | 9.46E-07 | -1.07 |
| *VLDLR* | 1.63E-07 | -1.76 | *C1orf189* | 1.52E-06 | -1.05 |
| *MPP5* | 5.09E-07 | -1.76 | *SAA2* | 1.43E-06 | -1.03 |
| *CRYAB* | 9.52E-08 | -1.76 | *SAA1* | 1.43E-06 | -1.03 |
| *CAV2* | 1.84E-07 | -1.75 | *C22orf23* | 2.95E-06 | -1.03 |
| *LMO4* | 1.58E-07 | -1.75 | *FBXO4* | 2.68E-06 | -1.01 |
| *VEGFB* | 1.54E-07 | -1.74 | *ATP6V0D2* | 2.34E-06 | -0.98 |
| *FAM198B* | 4.49E-07 | -1.73 | *CYR61* | 1.89E-06 | -0.98 |
| *FAM189A2* | 1.45E-06 | -1.73 | *BTC* | 1.08E-06 | -0.97 |
| *TMEM116* | 2.22E-07 | -1.73 | *RND3* | 2.89E-06 | -0.95 |
| *FRAS1* | 4.40E-07 | -1.73 | *OSBPL6* | 2.25E-06 | -0.91 |
| *ANKRD29* | 1.09E-06 | -1.72 | *EWSR1* | 2.68E-06 | 0.86 |
| *ALDH2* | 1.75E-07 | -1.72 | *RNASE1* | 1.55E-06 | 0.88 |
| *TACSTD2* | 5.36E-07 | -1.72 | *ITGA6* | 2.57E-06 | 0.88 |
| *UBE2V2* | 2.14E-07 | -1.72 | *MLL5* | 2.47E-06 | 0.92 |
| *RNASE4* | 1.26E-07 | -1.72 | *RNASEH2B* | 1.08E-06 | 0.95 |
| *TMBIM4* | 1.22E-07 | -1.70 | *CYBRD1* | 2.02E-06 | 0.95 |
| *SH3YL1* | 1.02E-07 | -1.70 | *TLN2* | 1.62E-06 | 0.95 |
| *TMOD1* | 1.03E-06 | -1.68 | *GCSH* | 2.33E-06 | 0.96 |
| *PRMT8* | 3.87E-07 | -1.68 | *RC3H2* | 9.15E-07 | 0.96 |
| *LUM* | 3.80E-07 | -1.68 | *C1QTNF7* | 2.41E-06 | 1.00 |
| *C1GALT1C1* | 8.23E-07 | -1.68 | *FAM105B* | 9.94E-07 | 1.01 |
| *RNF34* | 3.83E-07 | -1.67 | *CHRM4* | 9.77E-07 | 1.01 |
| *CXADR* | 2.42E-08 | -1.66 | *GFRA3* | 2.89E-06 | 1.01 |
| *OGN* | 1.55E-07 | -1.66 | *H6PD* | 1.27E-06 | 1.03 |
| *AQP2* | 5.68E-07 | -1.65 | *SCARB1* | 1.27E-06 | 1.03 |
| *EVA1C* | 2.47E-07 | -1.65 | *HIGD1B* | 1.25E-06 | 1.03 |
| *CES2* | 3.78E-07 | -1.65 | *ZNF503* | 2.44E-06 | 1.04 |
| *DSTN* | 4.73E-08 | -1.64 | *SRGN* | 1.98E-06 | 1.04 |
| *PON1* | 6.78E-08 | -1.64 | *PRKCB* | 2.52E-06 | 1.04 |
| *SOD1* | 2.03E-08 | -1.63 | *GSN* | 1.41E-06 | 1.05 |
| *CCDC110* | 3.47E-07 | -1.63 | *C22orf39* | 1.25E-06 | 1.06 |
| *HMGN3* | 1.04E-07 | -1.63 | *ADH1B* | 2.28E-06 | 1.06 |
| *DCN* | 2.45E-07 | -1.63 | *ADH1A* | 2.28E-06 | 1.06 |
| *TRIM2* | 8.44E-08 | -1.62 | *KDELC1* | 1.84E-06 | 1.07 |
| *RAB11FIP1* | 1.23E-07 | -1.62 | *HEG1* | 1.41E-06 | 1.08 |
| *ALDH1A1* | 7.25E-07 | -1.62 | *RASGRP2* | 1.90E-06 | 1.08 |
| *FAM213A* | 3.05E-07 | -1.62 | *CTNNBIP1* | 2.56E-06 | 1.08 |
| *SMG7* | 1.19E-07 | -1.61 | *CALD1* | 2.31E-06 | 1.08 |
| *BSN* | 1.19E-07 | -1.61 | *UNC5A* | 1.70E-06 | 1.09 |
| *TMEM9B* | 1.19E-07 | -1.61 | *PRKCDBP* | 1.42E-06 | 1.10 |
| *RBM33* | 1.19E-07 | -1.61 | *IGHV4-4* | 8.01E-07 | 1.11 |
| *SPAG6* | 1.17E-07 | -1.61 | *IGHV4-39* | 8.01E-07 | 1.11 |
| *CALM2* | 5.97E-08 | -1.60 | *IGHV4-28* | 8.01E-07 | 1.11 |
| *FBP2* | 1.11E-06 | -1.60 | *IGHV4-61* | 8.01E-07 | 1.11 |
| *ATP13A5* | 6.66E-08 | -1.60 | *IGHV6-1* | 8.01E-07 | 1.11 |
| *GKAP1* | 1.90E-07 | -1.60 | *IGHV4-31* | 8.01E-07 | 1.11 |
| *NDUFA4* | 4.50E-08 | -1.60 | *IGHV4-59* | 8.01E-07 | 1.11 |
| *SFTPC* | 4.97E-07 | -1.60 | *IGHV4-34* | 8.01E-07 | 1.11 |
| *MFGE8* | 2.22E-06 | -1.60 | *VWF* | 9.38E-07 | 1.11 |
| *CRYBG3* | 1.08E-06 | -1.59 | *EFNB2* | 9.14E-07 | 1.12 |
| *DUSP26* | 1.07E-06 | -1.59 | *ADAM2* | 2.70E-06 | 1.12 |
| *CEP68* | 4.20E-07 | -1.59 | *STMN1* | 3.25E-07 | 1.12 |
| *RAB1A* | 4.20E-07 | -1.59 | *ICAM2* | 1.93E-06 | 1.13 |
| *CTTNBP2NL* | 5.30E-07 | -1.58 | *PGA3* | 2.08E-06 | 1.13 |
| *TMEM98* | 3.68E-07 | -1.57 | *PGA4* | 2.08E-06 | 1.13 |
| *FABP3* | 5.40E-07 | -1.57 | *PGA5* | 2.08E-06 | 1.13 |
| *MATN2* | 1.53E-06 | -1.57 | *INMT* | 9.54E-07 | 1.13 |
| *PTPRF* | 1.13E-06 | -1.57 | *ITGA1* | 1.05E-06 | 1.14 |
| *MFAP4* | 5.20E-08 | -1.57 | *AGTPBP1* | 9.26E-07 | 1.14 |
| *GNAI2* | 5.20E-08 | -1.57 | *APCDD1* | 1.62E-06 | 1.15 |
| *GPI* | 5.20E-08 | -1.57 | *DPY19L1* | 2.74E-06 | 1.15 |
| *MGST1* | 1.86E-07 | -1.57 | *FAM26F* | 1.17E-06 | 1.15 |
| *SCD* | 2.59E-08 | -1.57 | *SNTB2* | 1.60E-06 | 1.15 |
| *CCDC173* | 8.93E-07 | -1.55 | *KATNBL1* | 1.49E-06 | 1.15 |
| *CCPG1* | 1.43E-07 | -1.54 | *NPNT* | 1.71E-06 | 1.17 |
| *TSPAN13* | 1.81E-07 | -1.54 | *EPB41* | 3.73E-07 | 1.17 |
| *TMEM106B* | 5.30E-07 | -1.53 | *ARL6IP5* | 1.94E-06 | 1.18 |
| *SLC12A2* | 3.99E-07 | -1.53 | *PF4V1* | 8.35E-07 | 1.18 |
| *AMOT* | 8.52E-07 | -1.53 | *PF4* | 8.35E-07 | 1.18 |
| *SPTBN1* | 8.52E-07 | -1.53 | *PLEKHO2* | 5.30E-07 | 1.18 |
| *GPR22* | 8.52E-07 | -1.53 | *TBC1D13* | 5.38E-07 | 1.18 |
| *RPL24* | 1.30E-07 | -1.53 | *FSTL1* | 2.13E-06 | 1.19 |
| *PLS3* | 9.91E-07 | -1.52 | *RECQL5* | 1.22E-06 | 1.19 |
| *CD36* | 6.28E-07 | -1.52 | *SLC25A38* | 2.26E-06 | 1.20 |
| *ITM2A* | 3.39E-07 | -1.52 | *ACTC1* | 2.03E-06 | 1.20 |
| *ADH1B* | 1.49E-07 | -1.52 | *CDC26* | 1.27E-06 | 1.20 |
| *ADH1A* | 1.49E-07 | -1.52 | *TMEM173* | 1.74E-06 | 1.20 |
| *BCAR3* | 1.56E-06 | -1.52 | *SEMA4C* | 6.51E-07 | 1.20 |
| *APP* | 1.15E-06 | -1.51 | *CD3D* | 2.29E-06 | 1.20 |
| *WDR63* | 4.02E-07 | -1.51 | *PPP2R5E* | 8.29E-07 | 1.20 |
| *PTCHD1* | 8.05E-07 | -1.51 | *TRAT1* | 1.15E-06 | 1.21 |
| *KCNE2* | 5.18E-07 | -1.51 | *LMBR1L* | 1.41E-06 | 1.21 |
| *PEG10* | 2.38E-07 | -1.50 | *S100A8* | 3.07E-07 | 1.21 |
| *ITM2B* | 2.38E-07 | -1.50 | *TMEM54* | 7.11E-07 | 1.22 |
| *LAMP3* | 1.20E-07 | -1.50 | *CD81* | 8.33E-07 | 1.22 |
| *RNF11* | 1.02E-07 | -1.50 | *CD3G* | 1.17E-06 | 1.22 |
| *AS3MT* | 1.83E-06 | -1.50 | *JAM2* | 7.30E-07 | 1.23 |
| *FMO6P* | 1.72E-07 | -1.49 | *UACA* | 2.76E-06 | 1.23 |
| *BCHE* | 5.02E-07 | -1.49 | *RAMP1* | 2.28E-06 | 1.23 |
| *HSPB11* | 2.01E-07 | -1.49 | *CEP85L* | 1.09E-06 | 1.24 |
| *POR* | 5.86E-08 | -1.48 | *TCF12* | 9.31E-07 | 1.24 |
| *RADIL* | 1.03E-06 | -1.47 | *CCDC28B* | 1.96E-06 | 1.25 |
| *TTC6* | 1.84E-06 | -1.47 | *WWTR1* | 7.63E-07 | 1.25 |
| *NEK4* | 6.82E-07 | -1.47 | *C2CD3* | 1.09E-06 | 1.25 |
| *CLDN4* | 3.79E-07 | -1.46 | *KRT25* | 2.23E-06 | 1.25 |
| *TMEM59* | 1.01E-07 | -1.46 | *ACP2* | 3.68E-07 | 1.27 |
| *FMO2* | 1.45E-07 | -1.46 | *TMEM88* | 1.20E-06 | 1.27 |
| *BDNF* | 1.02E-07 | -1.46 | *AHDC1* | 9.84E-07 | 1.27 |
| *PPM1A* | 3.14E-07 | -1.45 | *SKAP2* | 5.29E-07 | 1.27 |
| *HCFC2* | 2.40E-06 | -1.45 | *ZNF346* | 5.75E-07 | 1.28 |
| *PHKB* | 2.26E-07 | -1.45 | *MAP7D3* | 8.48E-07 | 1.28 |
| *COL4A6* | 5.41E-07 | -1.45 | *CD79B* | 2.29E-06 | 1.28 |
| *CUL3* | 1.93E-06 | -1.44 | *RBM20* | 2.01E-07 | 1.28 |
| *CHMP2A* | 7.78E-08 | -1.44 | *DHDH* | 2.93E-06 | 1.28 |
| *PHYH* | 1.45E-06 | -1.44 | *MRE11A* | 2.20E-07 | 1.29 |
| *GPAM* | 1.12E-06 | -1.44 | *PTRF* | 1.93E-06 | 1.29 |
| *SULT1A3* | 1.55E-07 | -1.44 | *THYN1* | 5.93E-07 | 1.29 |
| *SULT1A2* | 1.55E-07 | -1.44 | *ABCG2* | 1.21E-07 | 1.29 |
| *SULT1A4* | 1.55E-07 | -1.44 | *EIF5A2* | 2.86E-06 | 1.30 |
| *SULT1A1* | 1.55E-07 | -1.44 | *ADRB3* | 2.89E-06 | 1.30 |
| *HMGCS2* | 1.23E-06 | -1.44 | *UGCG* | 4.11E-07 | 1.30 |
| *FAM149A* | 2.57E-07 | -1.44 | *SLC9B2* | 1.67E-06 | 1.31 |
| *NPEPPS* | 2.69E-06 | -1.43 | *TRAC* | 1.03E-06 | 1.31 |
| *CPQ* | 5.24E-07 | -1.43 | *GPR116* | 5.93E-07 | 1.31 |
| *ACYP1* | 1.68E-07 | -1.43 | *TGFBI* | 1.76E-07 | 1.31 |
| *ACAT1* | 4.65E-07 | -1.43 | *TREM1* | 6.65E-07 | 1.31 |
| *PMS1* | 2.47E-07 | -1.42 | *LRP5L* | 2.42E-06 | 1.32 |
| *RPL21* | 8.44E-08 | -1.42 | *LRP5* | 2.42E-06 | 1.32 |
| *DNAH6* | 1.33E-06 | -1.41 | *ZBTB10* | 2.94E-06 | 1.32 |
| *FRA10AC1* | 3.89E-07 | -1.41 | *INPP1* | 1.85E-06 | 1.33 |
| *WWC2* | 6.89E-07 | -1.41 | *POLD4* | 1.76E-06 | 1.33 |
| *BMI1* | 2.03E-06 | -1.41 | *AQP8* | 2.44E-06 | 1.33 |
| *DNAJC1* | 2.03E-06 | -1.41 | *BPI* | 1.13E-07 | 1.33 |
| *NCAM1* | 1.34E-07 | -1.40 | *NIPSNAP3A* | 1.41E-07 | 1.34 |
| *LIMCH1* | 1.34E-07 | -1.40 | *CSDC2* | 1.84E-06 | 1.34 |
| *DNAJB9* | 2.29E-07 | -1.40 | *ESYT1* | 2.78E-06 | 1.35 |
| *SLC6A4* | 1.33E-06 | -1.40 | *MOSPD1* | 2.04E-06 | 1.35 |
| *OAT* | 1.59E-07 | -1.40 | *MYLK* | 5.62E-07 | 1.35 |
| *ANKRD5* | 9.32E-07 | -1.40 | *MICAL3* | 2.76E-07 | 1.36 |
| *TMEM30A* | 1.25E-06 | -1.40 | *PMP22* | 2.76E-07 | 1.36 |
| *POLR2K* | 2.15E-06 | -1.40 | *CSNK1A1L* | 2.76E-07 | 1.36 |
| *PTP4A1* | 1.55E-06 | -1.39 | *SEC16B* | 5.65E-07 | 1.36 |
| *VEPH1* | 1.23E-06 | -1.39 | *NUAK1* | 4.45E-07 | 1.36 |
| *RSPO1* | 2.94E-06 | -1.39 | *IRX5* | 4.45E-07 | 1.36 |
| *HHIP* | 2.83E-06 | -1.39 | *OR2C3* | 1.30E-06 | 1.36 |
| *BCO2* | 1.79E-07 | -1.38 | *SGK1* | 6.07E-07 | 1.36 |
| *WDR35* | 5.85E-07 | -1.38 | *ZNF397* | 1.50E-06 | 1.36 |
| *TGFB2* | 2.18E-06 | -1.38 | *RAET1L* | 1.48E-06 | 1.37 |
| *GSK3B* | 1.27E-07 | -1.38 | *RAET1G* | 1.48E-06 | 1.37 |
| *LPL* | 3.98E-07 | -1.36 | *ULBP1* | 1.48E-06 | 1.37 |
| *SVIL* | 1.25E-06 | -1.36 | *ULBP2* | 1.48E-06 | 1.37 |
| *PON3* | 1.50E-07 | -1.36 | *AVPR2* | 1.68E-06 | 1.37 |
| *SEC62* | 1.96E-07 | -1.36 | *ROBO1* | 2.72E-06 | 1.37 |
| *NDUFB4* | 4.84E-07 | -1.35 | *SPARCL1* | 5.25E-07 | 1.37 |
| *RPS27L* | 7.49E-07 | -1.35 | *PIP5K1A* | 9.73E-07 | 1.37 |
| *C1QTNF2* | 4.65E-07 | -1.34 | *ANTXR1* | 3.48E-07 | 1.37 |
| *ARHGEF26* | 2.11E-06 | -1.34 | *P2RX3* | 2.66E-06 | 1.38 |
| *C7orf23* | 5.65E-07 | -1.33 | *ALKBH5* | 2.00E-06 | 1.38 |
| *SCGB1A1* | 6.01E-07 | -1.33 | *KIAA1875* | 5.86E-07 | 1.38 |
| *MED30* | 1.50E-06 | -1.33 | *FST* | 2.04E-07 | 1.38 |
| *ABLIM1* | 2.53E-07 | -1.33 | *GRPEL2* | 1.39E-06 | 1.38 |
| *OAS3* | 2.53E-07 | -1.33 | *RB1CC1* | 1.38E-06 | 1.39 |
| *FADS6* | 2.58E-06 | -1.32 | *DISP1* | 2.82E-07 | 1.39 |
| *STARD3NL* | 2.07E-06 | -1.32 | *RPL27A* | 2.31E-07 | 1.39 |
| *ARHGEF12* | 1.52E-06 | -1.32 | *CNRIP1* | 1.30E-06 | 1.40 |
| *BLOC1S6* | 1.52E-06 | -1.32 | *CIB2* | 5.59E-07 | 1.40 |
| *PSMD12* | 1.46E-07 | -1.32 | *PDLIM7* | 3.20E-07 | 1.40 |
| *WRB* | 4.53E-07 | -1.31 | *MCM10* | 1.63E-06 | 1.41 |
| *SULT1C4* | 1.72E-06 | -1.31 | *MARCO* | 4.35E-07 | 1.41 |
| *PEX2* | 1.07E-06 | -1.31 | *DEFB124* | 2.10E-06 | 1.41 |
| *MECOM* | 6.28E-07 | -1.31 | *RTP2* | 9.65E-07 | 1.41 |
| *NR3C2* | 2.24E-06 | -1.31 | *IL10RA* | 1.84E-06 | 1.41 |
| *UGP2* | 1.40E-06 | -1.31 | *FRRS1L* | 2.25E-06 | 1.42 |
| *ACADM* | 2.01E-06 | -1.30 | *HOXA4* | 1.41E-06 | 1.42 |
| *COL4A4* | 2.90E-06 | -1.30 | *PSMD9* | 6.18E-07 | 1.43 |
| *TMSB15A* | 1.54E-06 | -1.30 | *FOXN3* | 2.29E-06 | 1.43 |
| *CETN2* | 1.84E-06 | -1.30 | *ABI3* | 8.51E-07 | 1.43 |
| *BAG2* | 1.35E-06 | -1.29 | *LRRC10* | 1.28E-06 | 1.43 |
| *CETN3* | 1.67E-06 | -1.29 | *ATMIN* | 1.43E-07 | 1.43 |
| *HTATSF1* | 4.30E-07 | -1.29 | *SLC16A9* | 3.93E-07 | 1.43 |
| *GOLIM4* | 1.80E-07 | -1.29 | *SLC29A1* | 1.32E-06 | 1.43 |
| *NEDD9* | 1.93E-07 | -1.29 | *HLA-A* | 6.34E-08 | 1.44 |
| *MLF1* | 5.06E-07 | -1.28 | *CNOT4* | 5.41E-07 | 1.44 |
| *GABARAPL2* | 3.37E-07 | -1.28 | *EFNB1* | 1.72E-07 | 1.45 |
| *DCBLD2* | 2.99E-07 | -1.28 | *ELTD1* | 5.40E-07 | 1.45 |
| *SDCBP2* | 9.37E-07 | -1.28 | *SVEP1* | 3.10E-07 | 1.45 |
| *CCDC113* | 1.10E-06 | -1.28 | *MAP3K10* | 1.92E-06 | 1.45 |
| *BDH2* | 2.55E-07 | -1.28 | *KHDRBS3* | 1.46E-06 | 1.45 |
| *ZHX1* | 2.68E-06 | -1.28 | *GATA3* | 7.31E-07 | 1.46 |
| *PLSCR1* | 1.06E-06 | -1.27 | *NID1* | 2.38E-07 | 1.46 |
| *PLSCR2* | 1.06E-06 | -1.27 | *FBN1* | 1.06E-06 | 1.46 |
| *ASB14* | 4.77E-07 | -1.27 | *ASS1* | 1.35E-06 | 1.47 |
| *SKP1* | 8.49E-07 | -1.27 | *KPNA4* | 4.38E-07 | 1.47 |
| *TCEAL8* | 2.37E-06 | -1.27 | *SLC8A1* | 4.38E-07 | 1.47 |
| *CCDC23* | 7.96E-07 | -1.27 | *KANK3* | 2.65E-06 | 1.47 |
| *FAM192A* | 2.27E-06 | -1.27 | *TUFT1* | 2.52E-06 | 1.47 |
| *HOXB2* | 2.27E-06 | -1.27 | *TSPAN6* | 7.26E-07 | 1.47 |
| *ZNF706* | 3.39E-07 | -1.27 | *FAM60A* | 2.57E-06 | 1.47 |
| *FAM162A* | 4.43E-07 | -1.27 | *TLE4* | 7.73E-08 | 1.47 |
| *SLC39A8* | 2.33E-06 | -1.27 | *TLE1* | 7.73E-08 | 1.47 |
| *DIO2* | 2.73E-07 | -1.27 | *COL8A2* | 1.65E-07 | 1.48 |
| *CAPN2* | 7.65E-07 | -1.27 | *ALKBH1* | 2.52E-06 | 1.48 |
| *TMEM126A* | 2.03E-06 | -1.26 | *SLC38A11* | 1.17E-06 | 1.48 |
| *PAPSS2* | 2.08E-06 | -1.26 | *TM4SF20* | 3.14E-07 | 1.48 |
| *VPS53* | 3.18E-07 | -1.26 | *BMP4* | 1.84E-07 | 1.48 |
| *GNG12* | 3.13E-07 | -1.25 | *YAF2* | 2.39E-07 | 1.48 |
| *OSBPL6* | 1.94E-07 | -1.25 | *CADM1* | 2.39E-07 | 1.48 |
| *PPAP2A* | 2.40E-06 | -1.25 | *C17orf103* | 2.39E-07 | 1.48 |
| *EML1* | 5.68E-07 | -1.25 | *LDLRAD2* | 8.31E-07 | 1.49 |
| *ANXA5* | 8.09E-07 | -1.25 | *DIP2A* | 1.40E-07 | 1.49 |
| *NEK1* | 3.99E-07 | -1.25 | *DIP2B* | 1.40E-07 | 1.49 |
| *TMEM14A* | 1.27E-06 | -1.25 | *TMEM37* | 3.95E-07 | 1.49 |
| *TUSC3* | 1.85E-06 | -1.25 | *C17orf107* | 4.56E-07 | 1.49 |
| *TIMP3* | 1.31E-06 | -1.24 | *RERG* | 9.45E-07 | 1.49 |
| *KRT1* | 1.12E-06 | -1.24 | *PCYOX1L* | 1.45E-06 | 1.49 |
| *KRT6C* | 1.12E-06 | -1.24 | *TMEM204* | 4.06E-07 | 1.49 |
| *KRT6A* | 1.12E-06 | -1.24 | *PTH1R* | 2.53E-07 | 1.50 |
| *KRT6B* | 1.12E-06 | -1.24 | *DUSP15* | 9.55E-08 | 1.50 |
| *CISD1* | 1.99E-06 | -1.24 | *MAZ* | 9.55E-08 | 1.50 |
| *MRPL48* | 3.63E-07 | -1.24 | *ZNF703* | 9.55E-08 | 1.50 |
| *MCFD2* | 1.57E-06 | -1.24 | *TMPO* | 6.67E-07 | 1.50 |
| *CYR61* | 3.26E-07 | -1.23 | *SLC13A3* | 1.55E-06 | 1.50 |
| *HBB* | 2.68E-06 | -1.23 | *CMTM5* | 3.14E-07 | 1.50 |
| *HBD* | 2.68E-06 | -1.23 | *EPHA4* | 2.49E-07 | 1.50 |
| *TM9SF2* | 3.76E-07 | -1.23 | *DAPL1* | 1.02E-07 | 1.50 |
| *AKTIP* | 1.97E-06 | -1.23 | *CYP11B1* | 8.53E-07 | 1.50 |
| *RND3* | 4.13E-07 | -1.23 | *CYP11B2* | 8.53E-07 | 1.50 |
| *PLP2* | 1.64E-06 | -1.23 | *ARHGEF25* | 3.87E-07 | 1.50 |
| *MDK* | 2.89E-06 | -1.22 | *PRR16* | 1.23E-06 | 1.50 |
| *CRYL1* | 4.19E-07 | -1.22 | *MS4A15* | 1.49E-06 | 1.51 |
| *VKORC1* | 5.31E-07 | -1.21 | *PDE4A* | 2.32E-07 | 1.51 |
| *KCTD1* | 4.31E-07 | -1.21 | *DGKH* | 8.62E-07 | 1.51 |
| *OSBPL3* | 1.01E-06 | -1.20 | *SLC6A4* | 7.09E-07 | 1.52 |
| *C6orf165* | 3.79E-07 | -1.20 | *TRBC2* | 4.97E-07 | 1.52 |
| *IL18* | 2.75E-07 | -1.20 | *PIEZO2* | 2.90E-07 | 1.52 |
| *LXN* | 2.63E-06 | -1.20 | *SGIP1* | 4.48E-07 | 1.52 |
| *TM2D1* | 2.47E-06 | -1.20 | *PALM2-AKAP2* | 1.11E-06 | 1.53 |
| *IRAK1BP1* | 5.27E-07 | -1.20 | *AKAP2* | 1.11E-06 | 1.53 |
| *LRRC36* | 7.07E-07 | -1.19 | *CEPT1* | 5.68E-07 | 1.53 |
| *S100A14* | 1.39E-06 | -1.19 | *ONECUT1* | 5.44E-07 | 1.53 |
| *RGCC* | 9.48E-07 | -1.19 | *FAM198A* | 6.58E-07 | 1.53 |
| *NIPA2* | 1.08E-06 | -1.18 | *FN1* | 5.82E-07 | 1.54 |
| *EMC2* | 1.30E-06 | -1.18 | *CBLN3* | 7.08E-07 | 1.54 |
| *NPNT* | 1.62E-06 | -1.18 | *CALCRL* | 1.15E-06 | 1.54 |
| *ATG10* | 1.16E-06 | -1.18 | *RNF144A* | 8.02E-08 | 1.55 |
| *RPS23* | 1.16E-06 | -1.18 | *COL11A2* | 7.57E-07 | 1.55 |
| *RPL34* | 4.43E-07 | -1.17 | *ITGA5* | 7.57E-07 | 1.55 |
| *WDR96* | 1.10E-06 | -1.17 | *KLF8* | 1.33E-06 | 1.55 |
| *DEGS1* | 2.80E-06 | -1.17 | *BASP1* | 4.50E-08 | 1.55 |
| *CITED1* | 3.66E-07 | -1.17 | *LPCAT4* | 1.50E-07 | 1.55 |
| *SFTPB* | 4.04E-07 | -1.17 | *LTBP1* | 3.64E-07 | 1.55 |
| *ST7* | 2.11E-06 | -1.17 | *FCF1* | 1.32E-06 | 1.55 |
| *EIF4A2* | 1.19E-06 | -1.17 | *PDIK1L* | 4.28E-07 | 1.56 |
| *CLIC4* | 1.03E-06 | -1.17 | *LPHN2* | 2.00E-07 | 1.56 |
| *OCIAD2* | 9.02E-07 | -1.17 | *KCNA2* | 8.77E-07 | 1.57 |
| *NDUFC2* | 1.43E-06 | -1.16 | *FYN* | 1.99E-07 | 1.57 |
| *NDUFC2-KCTD14* | 1.43E-06 | -1.16 | *FAM124B* | 1.07E-06 | 1.57 |
| *DNAJC19* | 7.77E-07 | -1.15 | *VTN* | 2.21E-07 | 1.57 |
| *RPS4XP21* | 1.58E-06 | -1.15 | *SLC36A4* | 9.16E-07 | 1.57 |
| *C5orf24* | 1.18E-06 | -1.15 | *TEK* | 1.24E-06 | 1.58 |
| *ALAD* | 2.05E-06 | -1.15 | *SEMA6C* | 8.07E-08 | 1.58 |
| *FAIM* | 2.80E-06 | -1.15 | *ITGB3* | 4.58E-08 | 1.59 |
| *TMEM163* | 2.15E-06 | -1.14 | *FGFBP1* | 2.71E-06 | 1.59 |
| *HSD17B4* | 1.51E-06 | -1.14 | *PODXL* | 1.45E-07 | 1.59 |
| *UBE2E1* | 2.08E-06 | -1.14 | *GJA5* | 7.24E-07 | 1.60 |
| *FBXL3* | 2.44E-06 | -1.14 | *DVL2* | 1.48E-06 | 1.60 |
| *STOX2* | 1.46E-06 | -1.14 | *IGSF10* | 1.37E-07 | 1.60 |
| *DERA* | 2.15E-06 | -1.13 | *SET* | 9.05E-08 | 1.60 |
| *PEX7* | 2.04E-06 | -1.13 | *HOXC11* | 1.54E-06 | 1.61 |
| *CAMK2D* | 5.24E-07 | -1.13 | *APOD* | 1.17E-07 | 1.61 |
| *COL4A5* | 2.44E-06 | -1.12 | *ATHL1* | 5.24E-07 | 1.61 |
| *LAPTM4A* | 8.37E-07 | -1.12 | *NADKD1* | 1.32E-07 | 1.61 |
| *LRIG3* | 1.38E-06 | -1.12 | *MYH10* | 6.01E-07 | 1.62 |
| *FAM82A1* | 4.97E-07 | -1.12 | *TREH* | 3.69E-07 | 1.62 |
| *NME7* | 1.10E-06 | -1.12 | *PRRT2* | 6.62E-07 | 1.62 |
| *PGM3* | 2.28E-06 | -1.11 | *QPCTL* | 3.11E-07 | 1.62 |
| *PGD* | 1.84E-06 | -1.11 | *IGF2BP3* | 1.14E-06 | 1.62 |
| *MBIP* | 1.62E-06 | -1.11 | *FAT4* | 1.01E-07 | 1.63 |
| *MYO1E* | 1.17E-06 | -1.11 | *SLC25A47* | 1.15E-06 | 1.63 |
| *RHEB* | 4.71E-07 | -1.11 | *EDNRB* | 3.44E-07 | 1.63 |
| *MED31* | 2.19E-06 | -1.10 | *NCF1* | 2.50E-07 | 1.63 |
| *USB1* | 1.90E-06 | -1.10 | *HRASLS* | 5.26E-07 | 1.63 |
| *AKAP14* | 2.56E-06 | -1.09 | *OR13H1* | 2.48E-06 | 1.63 |
| *IGFBP6* | 1.43E-06 | -1.09 | *ADCY4* | 1.39E-07 | 1.63 |
| *TTPAL* | 9.33E-07 | -1.09 | *FURIN* | 1.15E-06 | 1.64 |
| *FAM134B* | 1.63E-06 | -1.09 | *ADAMTSL3* | 4.92E-08 | 1.64 |
| *CTNNA1* | 7.14E-07 | -1.08 | *IGF1R* | 6.32E-07 | 1.64 |
| *S100A13* | 1.95E-06 | -1.08 | *GIMAP5* | 3.24E-07 | 1.64 |
| *PTGES3* | 1.41E-06 | -1.08 | *ATXN7L1* | 2.92E-07 | 1.64 |
| *C22orf23* | 2.08E-06 | -1.08 | *ACTA2* | 1.70E-07 | 1.65 |
| *TXNIP* | 2.19E-06 | -1.08 | *ADAMTS6* | 1.67E-06 | 1.65 |
| *EPHX2* | 1.05E-06 | -1.08 | *LRAT* | 1.23E-06 | 1.65 |
| *RAB2A* | 1.27E-06 | -1.08 | *RGS5* | 4.31E-07 | 1.65 |
| *ALAS1* | 5.03E-07 | -1.08 | *BMPR2* | 3.65E-08 | 1.66 |
| *NCKAP1* | 1.79E-06 | -1.07 | *TGFBR3* | 4.12E-07 | 1.66 |
| *TMBIM6* | 2.24E-06 | -1.07 | *KCNQ1* | 4.95E-07 | 1.67 |
| *RPL35A* | 1.87E-06 | -1.07 | *KCNG4* | 6.10E-07 | 1.67 |
| *GSTM2* | 7.20E-07 | -1.07 | *RGR* | 2.66E-07 | 1.68 |
| *COMMD1* | 6.85E-07 | -1.06 | *VIPR1* | 3.62E-07 | 1.68 |
| *C7orf55* | 2.71E-06 | -1.05 | *OTUD7B* | 1.94E-06 | 1.68 |
| *CNN3* | 2.52E-06 | -1.05 | *CD300LG* | 2.74E-08 | 1.69 |
| *RPL7* | 1.47E-06 | -1.05 | *ARMC5* | 1.35E-06 | 1.69 |
| *IGFBP7* | 1.77E-06 | -1.05 | *LAMA2* | 9.99E-08 | 1.69 |
| *CAPNS1* | 9.97E-07 | -1.05 | *PTPN22* | 5.27E-08 | 1.69 |
| *IFT80* | 8.10E-07 | -1.05 | *KIAA1598* | 4.51E-07 | 1.70 |
| *RPL17* | 9.86E-07 | -1.05 | *LRP3* | 1.63E-07 | 1.70 |
| *REXO2* | 1.09E-06 | -1.05 | *ADAMTS1* | 5.03E-08 | 1.70 |
| *APOO* | 1.85E-06 | -1.04 | *DBH* | 1.03E-07 | 1.71 |
| *HSPE1* | 1.11E-06 | -1.04 | *NCAM2* | 2.74E-07 | 1.71 |
| *ACADL* | 8.66E-07 | -1.04 | *IL11RA* | 6.59E-07 | 1.71 |
| *ANXA4* | 1.47E-06 | -1.04 | *ASB1* | 9.61E-07 | 1.71 |
| *RPL22L1* | 1.95E-06 | -1.04 | *ZDHHC24* | 2.90E-06 | 1.71 |
| *KLHDC2* | 2.05E-06 | -1.03 | *FBXO2* | 1.30E-06 | 1.72 |
| *ARG2* | 2.94E-06 | -1.03 | *ROBO2* | 1.59E-07 | 1.72 |
| *STAG2* | 2.35E-06 | -1.03 | *CLDN11* | 1.00E-06 | 1.72 |
| *CWC27* | 2.00E-06 | -1.02 | *TXNDC2* | 6.16E-07 | 1.72 |
| *TMEM230* | 2.71E-06 | -1.02 | *HAUS6* | 5.13E-07 | 1.72 |
| *EMP1* | 1.21E-06 | -1.02 | *COL7A1* | 1.36E-07 | 1.72 |
| *PPP3CA* | 1.54E-06 | -1.02 | *ANKS1A* | 4.76E-07 | 1.73 |
| *RARRES2* | 1.33E-06 | -1.01 | *NUMB* | 7.05E-07 | 1.73 |
| *DCAF4L2* | 2.73E-06 | -1.01 | *LOXL2* | 7.09E-08 | 1.73 |
| *DCAF4* | 2.73E-06 | -1.01 | *CNNM3* | 1.34E-06 | 1.73 |
| *DCAF4L1* | 2.73E-06 | -1.01 | *PCDHB11* | 4.80E-07 | 1.74 |
| *RFK* | 2.73E-06 | -1.01 | *PCDHB12* | 4.80E-07 | 1.74 |
| *FKBP3* | 2.31E-06 | -1.01 | *ART1* | 1.86E-06 | 1.74 |
| *ZC2HC1A* | 2.79E-06 | -1.00 | *ESPL1* | 1.25E-06 | 1.74 |
| *FGF9* | 2.39E-06 | -0.99 | *CCL21* | 5.43E-08 | 1.75 |
| *VAMP7* | 2.39E-06 | -0.99 | *MAD2L1BP* | 1.39E-06 | 1.75 |
| *UBXN4* | 1.51E-06 | -0.98 | *ASB13* | 4.62E-08 | 1.75 |
| *SAP18* | 1.48E-06 | -0.98 | *TAGLN* | 5.88E-08 | 1.75 |
| *SHOC2* | 1.25E-06 | -0.98 | *ADAMTS3* | 8.24E-07 | 1.75 |
| *TXNDC17* | 1.69E-06 | -0.98 | *SHKBP1* | 2.13E-07 | 1.75 |
| *NDFIP1* | 2.22E-06 | -0.97 | *TBATA* | 2.60E-07 | 1.75 |
| *ACN9* | 9.66E-07 | -0.97 | *TNS1* | 2.00E-08 | 1.76 |
| *RPL26* | 1.83E-06 | -0.97 | *TCN2* | 9.19E-07 | 1.76 |
| *RPL37A* | 2.21E-06 | -0.95 | *SLC47A1* | 6.36E-07 | 1.76 |
| *DYNC2LI1* | 2.87E-06 | -0.94 | *SUV420H1* | 1.62E-06 | 1.76 |
| *ANKRD50* | 1.58E-06 | -0.93 | *ASB4* | 3.09E-07 | 1.77 |
| *GRAMD1B* | 2.34E-06 | -0.92 | *TRIOBP* | 5.85E-07 | 1.77 |
| *RPL23* | 1.91E-06 | -0.91 | *C2orf40* | 4.33E-08 | 1.78 |
| *MYO1C* | 1.84E-06 | -0.90 | *FHL3* | 7.03E-08 | 1.78 |
| *ROMO1* | 2.41E-06 | -0.85 | *EME1* | 6.99E-07 | 1.78 |
| *SLC25A4* | 2.59E-06 | -0.84 | *ADD2* | 2.39E-06 | 1.78 |
| *S100A4* | 2.21E-06 | 0.91 | *CA14* | 7.10E-07 | 1.79 |
| *EWSR1* | 1.56E-06 | 0.93 | *TERF1* | 7.10E-07 | 1.79 |
| *USP4* | 2.69E-06 | 0.93 | *TBX3* | 1.71E-06 | 1.79 |
| *PCDH17* | 1.73E-06 | 0.94 | *ZNF808* | 7.11E-08 | 1.80 |
| *TNFRSF1A* | 2.07E-06 | 0.99 | *ZNF646* | 1.65E-07 | 1.80 |
| *TGFBI* | 1.48E-06 | 0.99 | *SLC22A9* | 1.00E-06 | 1.80 |
| *ZC3H11A* | 2.81E-06 | 1.00 | *SLC22A25* | 1.00E-06 | 1.80 |
| *RECQL* | 1.39E-06 | 1.00 | *CCDC117* | 6.65E-07 | 1.80 |
| *SWAP70* | 1.29E-06 | 1.00 | *THOC5* | 7.61E-07 | 1.81 |
| *SNRNP48* | 1.80E-06 | 1.01 | *ATP2B1* | 4.98E-07 | 1.81 |
| *RNF144A* | 1.88E-06 | 1.02 | *RPAP3* | 1.07E-07 | 1.81 |
| *AP1S2* | 1.11E-06 | 1.03 | *SPATA5* | 5.56E-07 | 1.81 |
| *CKLF* | 1.17E-06 | 1.04 | *GPX3* | 5.29E-08 | 1.81 |
| *ATP6V0C* | 1.37E-06 | 1.05 | *ELF2* | 2.51E-07 | 1.82 |
| *S100A12* | 1.06E-06 | 1.06 | *TAS1R2* | 6.63E-07 | 1.82 |
| *SNTB2* | 2.82E-06 | 1.07 | *SLC25A44* | 5.71E-08 | 1.82 |
| *STX17* | 2.53E-06 | 1.09 | *RASGRP1* | 2.97E-08 | 1.82 |
| *PYCARD* | 1.03E-06 | 1.15 | *GPR174* | 5.89E-07 | 1.83 |
| *CDK1* | 1.09E-06 | 1.15 | *CACNA1C* | 2.89E-06 | 1.83 |
| *BLNK* | 3.33E-07 | 1.15 | *SCN9A* | 5.42E-07 | 1.83 |
| *MRPL17* | 1.61E-06 | 1.15 | *MYH11* | 6.44E-08 | 1.83 |
| *BCL10* | 2.17E-06 | 1.15 | *NBR1* | 1.63E-06 | 1.83 |
| *PPP2R5C* | 2.59E-06 | 1.16 | *ACE* | 7.93E-09 | 1.84 |
| *CTSS* | 2.77E-06 | 1.16 | *C1orf95* | 1.37E-06 | 1.84 |
| *RECQL5* | 1.52E-06 | 1.16 | *ATL2* | 5.54E-07 | 1.84 |
| *PAPD4* | 1.51E-06 | 1.16 | *SMTN* | 5.38E-07 | 1.84 |
| *ERO1L* | 2.59E-06 | 1.16 | *SMTNL2* | 5.38E-07 | 1.84 |
| *NUAK1* | 1.40E-06 | 1.17 | *PDGFB* | 5.38E-07 | 1.84 |
| *IRX5* | 1.40E-06 | 1.17 | *RIMS2* | 2.76E-06 | 1.85 |
| *A2M* | 1.83E-06 | 1.17 | *IL6R* | 8.49E-07 | 1.85 |
| *MSH3* | 2.24E-06 | 1.17 | *GALNTL4* | 7.17E-07 | 1.85 |
| *RNF114* | 5.77E-07 | 1.18 | *ZNF697* | 2.28E-07 | 1.85 |
| *DCP1A* | 1.45E-06 | 1.19 | *GNG11* | 9.71E-08 | 1.85 |
| *CRY2* | 1.96E-06 | 1.19 | *COL1A1* | 1.01E-08 | 1.85 |
| *COL1A2* | 4.45E-07 | 1.19 | *GTF3C4* | 1.01E-08 | 1.85 |
| *LGALS3* | 1.13E-06 | 1.21 | *FAM210B* | 7.56E-07 | 1.86 |
| *SATB1* | 2.36E-06 | 1.21 | *COL24A1* | 9.22E-08 | 1.86 |
| *CEP170* | 4.51E-07 | 1.21 | *ABCA6* | 2.01E-06 | 1.86 |
| *C1S* | 1.79E-06 | 1.21 | *DOLK* | 2.82E-06 | 1.86 |
| *GPR19* | 1.14E-06 | 1.21 | *KRT31* | 2.88E-07 | 1.87 |
| *AIM1* | 1.20E-06 | 1.22 | *KRT34* | 2.88E-07 | 1.87 |
| *C10orf137* | 1.76E-06 | 1.22 | *KRT33A* | 2.88E-07 | 1.87 |
| *SRGN* | 5.90E-07 | 1.22 | *KRT33B* | 2.88E-07 | 1.87 |
| *SLC25A34* | 5.07E-07 | 1.22 | *TMEM2* | 3.21E-08 | 1.87 |
| *RAB13* | 1.46E-06 | 1.23 | *FIGF* | 7.33E-07 | 1.88 |
| *VTN* | 1.47E-06 | 1.23 | *SLC40A1* | 4.71E-07 | 1.88 |
| *COL24A1* | 2.21E-06 | 1.23 | *FAM117A* | 1.27E-06 | 1.88 |
| *NCAPG2* | 2.22E-06 | 1.23 | *OR8B1P* | 2.49E-06 | 1.89 |
| *FOXN2* | 7.32E-07 | 1.23 | *TMEM222* | 8.09E-07 | 1.89 |
| *COL3A1* | 7.21E-07 | 1.23 | *NCKAP5L* | 7.36E-08 | 1.89 |
| *VPS36* | 1.60E-06 | 1.23 | *FRMD5* | 5.05E-08 | 1.90 |
| *ZNF366* | 4.58E-07 | 1.24 | *LRRC8B* | 2.56E-07 | 1.90 |
| *TPI1* | 3.46E-07 | 1.24 | *PCDH17* | 7.60E-09 | 1.90 |
| *MDM4* | 2.22E-06 | 1.24 | *S100A9* | 3.66E-07 | 1.90 |
| *EVI2A* | 1.29E-06 | 1.25 | *SRRM5* | 4.38E-08 | 1.90 |
| *CERS6* | 2.82E-06 | 1.25 | *CSPG4* | 1.81E-07 | 1.91 |
| *UGCG* | 5.30E-07 | 1.26 | *KRTAP9-4* | 1.35E-06 | 1.91 |
| *MSL1* | 2.14E-06 | 1.26 | *KRTAP9-6* | 1.35E-06 | 1.91 |
| *SPAG1* | 1.35E-06 | 1.27 | *KRTAP9-7* | 1.35E-06 | 1.91 |
| *TMEM89* | 2.68E-06 | 1.27 | *KRTAP9-2* | 1.35E-06 | 1.91 |
| *SKAP2* | 5.54E-07 | 1.27 | *KRTAP9-3* | 1.35E-06 | 1.91 |
| *NCAM2* | 2.67E-06 | 1.27 | *KRTAP9-8* | 1.35E-06 | 1.91 |
| *FLT3* | 1.05E-06 | 1.28 | *KRTAP9-9* | 1.35E-06 | 1.91 |
| *CSNK1G1* | 9.05E-07 | 1.28 | *EFNA3* | 1.84E-06 | 1.92 |
| *TNFAIP8L2* | 1.60E-06 | 1.28 | *RXFP2* | 2.24E-06 | 1.92 |
| *PDLIM7* | 6.17E-07 | 1.29 | *PAIP2* | 1.58E-06 | 1.92 |
| *NSUN7* | 2.23E-06 | 1.29 | *CTLA4* | 1.69E-06 | 1.92 |
| *WDFY4* | 2.86E-06 | 1.29 | *NUPL2* | 7.97E-08 | 1.93 |
| *IGF1* | 1.67E-06 | 1.29 | *PRL* | 3.85E-07 | 1.93 |
| *CHST15* | 1.69E-06 | 1.30 | *HSPBAP1* | 1.47E-06 | 1.93 |
| *IL15* | 4.10E-07 | 1.30 | *COL6A1* | 6.25E-07 | 1.93 |
| *RAB33B* | 2.79E-06 | 1.30 | *DYRK4* | 1.47E-07 | 1.93 |
| *MTHFD1L* | 1.83E-06 | 1.31 | *MAOA* | 2.97E-07 | 1.94 |
| *CRYZ* | 8.46E-07 | 1.31 | *SLC2A4* | 2.97E-07 | 1.94 |
| *LGALS4* | 1.26E-06 | 1.31 | *TRBV30* | 2.39E-07 | 1.94 |
| *MCM10* | 2.75E-06 | 1.31 | *TIMP1* | 4.41E-07 | 1.94 |
| *ARAP2* | 1.01E-06 | 1.32 | *MYOCD* | 1.09E-08 | 1.94 |
| *CCDC85C* | 2.87E-06 | 1.32 | *SHC3* | 2.75E-06 | 1.95 |
| *HOXA9* | 2.87E-06 | 1.32 | *WDR87* | 1.84E-06 | 1.95 |
| *BMP1* | 5.86E-07 | 1.33 | *PALB2* | 6.60E-07 | 1.95 |
| *PSAP* | 1.24E-07 | 1.33 | *FAM96A* | 1.43E-06 | 1.95 |
| *TRIML2* | 1.54E-06 | 1.33 | *FUBP1* | 2.23E-07 | 1.95 |
| *MARCKSL1* | 2.36E-07 | 1.35 | *GHRHR* | 1.95E-06 | 1.95 |
| *MTF1* | 5.08E-07 | 1.35 | *GLIPR1* | 8.81E-07 | 1.95 |
| *STMN1* | 7.38E-08 | 1.36 | *14-Sep* | 2.87E-06 | 1.95 |
| *POLE* | 2.42E-06 | 1.37 | *RIPK4* | 2.41E-07 | 1.95 |
| *TMEM173* | 6.35E-07 | 1.37 | *SCOC* | 7.42E-08 | 1.96 |
| *LDLRAD2* | 1.52E-06 | 1.37 | *PXN* | 2.14E-06 | 1.96 |
| *TMEM74* | 2.60E-06 | 1.37 | *KRTAP4-16P* | 6.13E-07 | 1.96 |
| *TLR4* | 2.68E-06 | 1.38 | *KRTAP3-2* | 3.12E-07 | 1.96 |
| *KCNA2* | 2.35E-06 | 1.38 | *KRTAP3-3* | 3.12E-07 | 1.96 |
| *WDHD1* | 1.20E-06 | 1.38 | *BMF* | 1.28E-07 | 1.96 |
| *ABCC13* | 1.01E-06 | 1.38 | *SCN4B* | 2.24E-08 | 1.97 |
| *BPI* | 8.31E-08 | 1.38 | *GALR2* | 1.13E-06 | 1.98 |
| *GPC3* | 5.10E-07 | 1.39 | *OR8B12* | 3.49E-07 | 1.99 |
| *NOP56* | 2.87E-07 | 1.39 | *TSPYL1* | 3.85E-07 | 1.99 |
| *OTUD6A* | 4.79E-07 | 1.40 | *PNMAL1* | 3.56E-07 | 1.99 |
| *CCNB2* | 1.76E-06 | 1.40 | *FAM81B* | 1.43E-07 | 1.99 |
| *HPX* | 8.06E-07 | 1.40 | *DPPA2* | 5.96E-07 | 1.99 |
| *RDH10* | 3.98E-07 | 1.41 | *CTF1* | 6.65E-09 | 1.99 |
| *PYY* | 3.04E-07 | 1.41 | *RGS20* | 2.38E-06 | 1.99 |
| *SH2B3* | 4.67E-07 | 1.41 | *PXDNL* | 2.22E-06 | 1.99 |
| *BZW2* | 1.15E-06 | 1.42 | *COX7A2* | 1.56E-07 | 2.00 |
| *RABGGTA* | 8.03E-07 | 1.42 | *RPL14* | 1.56E-07 | 2.00 |
| *SH3BGRL3* | 6.20E-07 | 1.43 | *CTSD* | 1.56E-07 | 2.00 |
| *PSME1* | 2.61E-06 | 1.43 | *APH1B* | 4.59E-07 | 2.00 |
| *NCKAP5L* | 6.27E-07 | 1.43 | *GAS2* | 2.93E-06 | 2.01 |
| *LY96* | 8.54E-07 | 1.44 | *QKI* | 8.91E-08 | 2.01 |
| *ZNF217* | 8.82E-08 | 1.45 | *LAMA4* | 3.63E-07 | 2.01 |
| *CASP3* | 1.47E-06 | 1.45 | *MYADML2* | 2.59E-07 | 2.02 |
| *PTPN1* | 1.04E-06 | 1.45 | *COL16A1* | 4.83E-08 | 2.02 |
| *TRPV2* | 3.98E-07 | 1.45 | *ACTG2* | 1.63E-07 | 2.03 |
| *NF2* | 1.23E-06 | 1.45 | *RNF43* | 3.34E-07 | 2.03 |
| *ENTPD1* | 5.78E-07 | 1.45 | *GLYR1* | 4.87E-07 | 2.04 |
| *NABP1* | 1.18E-06 | 1.47 | *OSBP* | 1.53E-06 | 2.04 |
| *FTH1* | 1.68E-06 | 1.47 | *ECT2* | 1.72E-06 | 2.04 |
| *TMEM8B* | 4.55E-07 | 1.48 | *ZKSCAN5* | 2.95E-07 | 2.05 |
| *HIST1H4D* | 1.63E-06 | 1.49 | *WIPI2* | 1.11E-07 | 2.05 |
| *RSC1A1* | 2.22E-07 | 1.49 | *DCTN3* | 2.51E-07 | 2.05 |
| *IGF2BP3* | 2.10E-06 | 1.49 | *EPPIN* | 1.93E-07 | 2.05 |
| *SLC25A19* | 4.67E-07 | 1.50 | *CCDC41* | 2.01E-06 | 2.06 |
| *IL1R2* | 5.23E-08 | 1.50 | *LRRC4C* | 8.70E-07 | 2.06 |
| *SPP1* | 6.68E-07 | 1.50 | *VSTM2A* | 3.85E-07 | 2.06 |
| *TRBV5-6* | 2.53E-06 | 1.50 | *C1orf51* | 2.72E-07 | 2.06 |
| *TRBV5-7* | 2.53E-06 | 1.50 | *FBLN1* | 1.39E-07 | 2.07 |
| *TRBV5-1* | 2.53E-06 | 1.50 | *VPS36* | 2.94E-08 | 2.07 |
| *TRBV5-3* | 2.53E-06 | 1.50 | *IDE* | 4.58E-07 | 2.08 |
| *TRBV5-4* | 2.53E-06 | 1.50 | *GPR37* | 7.29E-09 | 2.08 |
| *TRBV5-5* | 2.53E-06 | 1.50 | *ENC1* | 3.58E-07 | 2.08 |
| *PILRB* | 1.49E-06 | 1.50 | *MAP7D1* | 2.08E-06 | 2.09 |
| *PILRA* | 1.49E-06 | 1.50 | *PALLD* | 1.28E-08 | 2.10 |
| *ACTR3* | 1.45E-07 | 1.50 | *GABRQ* | 6.15E-07 | 2.11 |
| *CNPY3* | 1.41E-06 | 1.51 | *TPST1* | 6.88E-07 | 2.11 |
| *NMI* | 3.72E-07 | 1.51 | *USP19* | 1.84E-08 | 2.11 |
| *AGR2* | 2.27E-06 | 1.51 | *KCNMB2* | 1.07E-07 | 2.11 |
| *DENND2D* | 2.47E-08 | 1.51 | *NSUN7* | 5.13E-08 | 2.11 |
| *ARCN1* | 5.03E-07 | 1.52 | *CLDN12* | 1.74E-06 | 2.11 |
| *SH3KBP1* | 3.37E-07 | 1.52 | *OTOP2* | 1.33E-06 | 2.12 |
| *OSTM1* | 2.79E-06 | 1.52 | *CPE* | 6.96E-07 | 2.12 |
| *BUB1B* | 1.27E-07 | 1.52 | *F11R* | 1.06E-06 | 2.13 |
| *PITPNM2* | 2.92E-06 | 1.53 | *DOCK4* | 1.52E-07 | 2.13 |
| *RNF115* | 3.29E-07 | 1.53 | *PRKAB2* | 2.04E-07 | 2.13 |
| *BPIFB1* | 1.18E-07 | 1.54 | *GPR162* | 8.25E-07 | 2.14 |
| *LYN* | 2.54E-06 | 1.54 | *LIN28A* | 2.38E-07 | 2.14 |
| *MCOLN2* | 1.11E-06 | 1.54 | *LIN28AP1* | 2.38E-07 | 2.14 |
| *PCK2* | 3.14E-07 | 1.54 | *CD1A* | 4.82E-07 | 2.15 |
| *INHBA* | 2.01E-06 | 1.54 | *TMEM225* | 3.25E-07 | 2.15 |
| *YEATS4* | 7.51E-08 | 1.55 | *UBE2G1* | 2.85E-06 | 2.16 |
| *SLC27A1* | 2.67E-07 | 1.55 | *KRTAP11-1* | 2.37E-06 | 2.17 |
| *NSL1* | 1.64E-06 | 1.56 | *ART4* | 2.72E-07 | 2.17 |
| *LIMD2* | 9.19E-07 | 1.56 | *MEGF8* | 2.21E-06 | 2.17 |
| *MAN2B1* | 1.36E-06 | 1.56 | *COL4A2* | 3.48E-08 | 2.18 |
| *EPHX4* | 2.44E-07 | 1.56 | *WDR77* | 4.45E-07 | 2.19 |
| *OLFML2B* | 2.96E-07 | 1.56 | *FOXF1* | 1.46E-07 | 2.19 |
| *FAM81B* | 9.05E-07 | 1.56 | *SIGLEC10* | 1.42E-07 | 2.19 |
| *CXCL5* | 1.30E-06 | 1.57 | *SIGLEC11* | 1.42E-07 | 2.19 |
| *CXCL6* | 1.30E-06 | 1.57 | *FBXL7* | 1.19E-07 | 2.19 |
| *TBC1D13* | 6.00E-08 | 1.57 | *HDAC7* | 1.04E-08 | 2.19 |
| *SERPINE1* | 2.90E-07 | 1.57 | *NUS1* | 1.04E-08 | 2.19 |
| *KIF26B* | 1.10E-06 | 1.58 | *9-Mar* | 1.52E-08 | 2.19 |
| *BAZ1A* | 4.84E-07 | 1.58 | *DPPA4* | 1.97E-06 | 2.19 |
| *FAS* | 3.19E-07 | 1.58 | *OR2T27* | 6.52E-08 | 2.20 |
| *TSPO* | 8.11E-07 | 1.58 | *UBXN6* | 7.19E-07 | 2.20 |
| *HLA-DQB1* | 5.65E-07 | 1.58 | *CRYGC* | 8.68E-07 | 2.20 |
| *HLA-DQB2* | 5.65E-07 | 1.58 | *GPR158* | 1.29E-06 | 2.20 |
| *CSTB* | 4.10E-08 | 1.58 | *OR52N2* | 2.01E-06 | 2.20 |
| *CASP8* | 7.93E-07 | 1.59 | *CIT* | 1.06E-07 | 2.21 |
| *CORO6* | 1.12E-06 | 1.59 | *TFAP2A* | 8.78E-07 | 2.21 |
| *PIP4K2A* | 1.03E-06 | 1.59 | *FBXL20* | 3.22E-07 | 2.21 |
| *IL8* | 9.84E-07 | 1.59 | *CDH16* | 1.50E-06 | 2.22 |
| *NDC80* | 6.43E-07 | 1.60 | *IGHG1* | 8.39E-09 | 2.22 |
| *CAPG* | 3.72E-07 | 1.60 | *IGHG2* | 8.39E-09 | 2.22 |
| *FCGR3A* | 4.50E-07 | 1.60 | *IGHG3* | 8.39E-09 | 2.22 |
| *FCGR3B* | 4.50E-07 | 1.60 | *IGHG4* | 8.39E-09 | 2.22 |
| *GIT2* | 1.28E-06 | 1.61 | *ZNF75A* | 2.80E-07 | 2.22 |
| *WARS* | 1.74E-07 | 1.61 | *OSMR* | 4.11E-07 | 2.22 |
| *GPR15* | 4.92E-07 | 1.61 | *PIPOX* | 1.24E-06 | 2.23 |
| *FNBP4* | 2.01E-07 | 1.61 | *EGFEM1P* | 2.11E-08 | 2.23 |
| *DGAT2* | 2.44E-07 | 1.61 | *CKM* | 2.14E-08 | 2.23 |
| *CIT* | 1.15E-06 | 1.62 | *LRRTM4* | 1.45E-06 | 2.23 |
| *DOCK11* | 2.03E-07 | 1.62 | *THRSP* | 2.81E-07 | 2.23 |
| *PALLD* | 9.71E-08 | 1.62 | *MRC2* | 1.86E-07 | 2.24 |
| *PSME2* | 2.26E-08 | 1.63 | *ERN1* | 6.44E-07 | 2.24 |
| *GRN* | 5.97E-08 | 1.63 | *PATZ1* | 4.92E-07 | 2.24 |
| *SLC4A7* | 5.28E-07 | 1.63 | *CD248* | 4.58E-09 | 2.24 |
| *WIPF1* | 4.80E-07 | 1.63 | *MAP2K4* | 1.02E-06 | 2.24 |
| *NCAPH* | 1.86E-06 | 1.63 | *THSD1* | 1.20E-08 | 2.25 |
| *PTGER4* | 5.53E-08 | 1.64 | *TESPA1* | 3.37E-07 | 2.25 |
| *PGLYRP1* | 7.12E-07 | 1.65 | *MSX2* | 1.83E-06 | 2.25 |
| *COL7A1* | 1.95E-07 | 1.65 | *GMNC* | 4.63E-07 | 2.25 |
| *CYP7A1* | 5.64E-07 | 1.65 | *COL15A1* | 1.82E-08 | 2.25 |
| *FAM49B* | 1.07E-06 | 1.65 | *MTR* | 1.95E-07 | 2.27 |
| *TMCO4* | 1.62E-07 | 1.65 | *GTF2H2* | 6.29E-07 | 2.27 |
| *FXYD1* | 1.89E-07 | 1.66 | *GTF2H2C* | 6.29E-07 | 2.27 |
| *FXYD5* | 2.16E-07 | 1.66 | *SERINC1* | 7.55E-07 | 2.27 |
| *FUT1* | 2.76E-07 | 1.67 | *HCN1* | 8.38E-07 | 2.28 |
| *SAMHD1* | 2.82E-07 | 1.67 | *NF2* | 3.82E-08 | 2.28 |
| *KPNA4* | 1.59E-07 | 1.67 | *NACC2* | 3.25E-08 | 2.28 |
| *SLC8A1* | 1.59E-07 | 1.67 | *SPTA1* | 3.81E-07 | 2.29 |
| *C1QTNF1* | 3.53E-08 | 1.68 | *HPDL* | 2.05E-06 | 2.29 |
| *FRMD5* | 1.29E-07 | 1.68 | *CD55* | 1.45E-07 | 2.29 |
| *TRGV10* | 1.20E-07 | 1.68 | *PLA2G4B* | 1.43E-06 | 2.30 |
| *ARHGAP25* | 2.28E-07 | 1.68 | *JMJD7-PLA2G4B* | 1.43E-06 | 2.30 |
| *BASP1* | 2.30E-08 | 1.69 | *RASSF6* | 7.70E-07 | 2.30 |
| *NFKB2* | 1.43E-07 | 1.69 | *COL3A1* | 5.68E-09 | 2.30 |
| *SFTPA1* | 4.34E-08 | 1.69 | *THEM5* | 1.49E-06 | 2.31 |
| *SFTPA2* | 4.34E-08 | 1.69 | *BIRC6* | 3.99E-07 | 2.31 |
| *TOP2A* | 4.35E-07 | 1.69 | *PLEKHG2* | 2.74E-07 | 2.31 |
| *CCDC69* | 9.28E-07 | 1.70 | *HOXC6* | 2.11E-07 | 2.31 |
| *FBXL20* | 2.41E-06 | 1.70 | *SLC5A1* | 1.35E-06 | 2.33 |
| *CIITA* | 1.95E-07 | 1.70 | *ARL4A* | 6.83E-08 | 2.33 |
| *CFB* | 3.42E-07 | 1.71 | *OR2W1* | 2.44E-07 | 2.34 |
| *TNFAIP8* | 4.25E-07 | 1.71 | *ENTPD3* | 2.63E-06 | 2.35 |
| *JAK2* | 1.82E-06 | 1.71 | *OR4N5* | 2.15E-07 | 2.35 |
| *CREB3L1* | 1.30E-06 | 1.72 | *ZBTB22* | 3.78E-07 | 2.35 |
| *CFP* | 4.53E-07 | 1.72 | *GIPR* | 4.93E-07 | 2.36 |
| *NCKAP1L* | 1.89E-07 | 1.72 | *AGXT* | 1.30E-07 | 2.36 |
| *CCDC71L* | 1.02E-06 | 1.73 | *KIF26B* | 4.88E-08 | 2.36 |
| *HNMT* | 6.57E-08 | 1.73 | *EXTL3* | 4.32E-07 | 2.36 |
| *RPAP3* | 1.47E-07 | 1.74 | *C15orf40* | 2.75E-07 | 2.36 |
| *ATP5B* | 4.86E-08 | 1.74 | *SFTPA1* | 3.16E-09 | 2.37 |
| *CRAMP1L* | 2.56E-06 | 1.75 | *SFTPA2* | 3.16E-09 | 2.37 |
| *PSMB8* | 2.87E-07 | 1.76 | *COL1A2* | 2.10E-09 | 2.37 |
| *NAMPT* | 2.18E-06 | 1.76 | *MOGAT3* | 5.50E-07 | 2.38 |
| *NAMPTL* | 2.18E-06 | 1.76 | *CLDN15* | 4.78E-07 | 2.38 |
| *C12orf40* | 3.11E-07 | 1.76 | *IGHV3-49* | 7.09E-08 | 2.38 |
| *MTHFD2* | 1.05E-06 | 1.76 | *CHRNA4* | 4.82E-08 | 2.38 |
| *S100A8* | 1.63E-08 | 1.77 | *OR6X1* | 5.14E-07 | 2.38 |
| *PGA3* | 6.65E-08 | 1.77 | *MEF2C* | 1.35E-06 | 2.39 |
| *PGA4* | 6.65E-08 | 1.77 | *NECAP2* | 3.53E-08 | 2.40 |
| *PGA5* | 6.65E-08 | 1.77 | *SLC24A4* | 1.02E-06 | 2.41 |
| *ANKRD13B* | 1.03E-06 | 1.78 | *FSTL5* | 2.91E-06 | 2.41 |
| *GAPDHP65* | 4.00E-08 | 1.78 | *ATP5B* | 3.91E-09 | 2.41 |
| *GAPDH* | 4.00E-08 | 1.78 | *FAM120A* | 1.76E-06 | 2.41 |
| *RAC2* | 1.15E-07 | 1.79 | *IL1RAP* | 5.66E-07 | 2.41 |
| *COL11A2* | 2.50E-07 | 1.79 | *SPTSSB* | 2.15E-07 | 2.42 |
| *ITGA5* | 2.50E-07 | 1.79 | *IGHE* | 4.22E-08 | 2.42 |
| *CTSE* | 6.21E-07 | 1.79 | *PDGFRA* | 4.49E-08 | 2.42 |
| *CD163* | 7.52E-07 | 1.79 | *RBM10* | 3.66E-09 | 2.43 |
| *HLA-DMB* | 3.42E-08 | 1.80 | *CNOT1* | 3.66E-09 | 2.43 |
| *IGHV3OR16-6* | 1.18E-07 | 1.81 | *GPBAR1* | 1.58E-08 | 2.43 |
| *CD48* | 1.04E-07 | 1.81 | *GNG8* | 1.07E-06 | 2.44 |
| *CHIT1* | 2.81E-06 | 1.81 | *SLC22A3* | 2.00E-07 | 2.44 |
| *CD14* | 1.62E-08 | 1.81 | *OR5K3* | 6.37E-07 | 2.44 |
| *FBLN1* | 3.76E-07 | 1.82 | *KRT13* | 4.06E-07 | 2.44 |
| *SPTA1* | 2.16E-06 | 1.82 | *ZFHX4* | 1.78E-07 | 2.45 |
| *CDC20* | 1.89E-07 | 1.83 | *PHEX* | 1.32E-07 | 2.45 |
| *TLR10* | 2.74E-07 | 1.83 | *MYF6* | 9.86E-07 | 2.46 |
| *TAP2* | 1.06E-06 | 1.84 | *OR5C1* | 2.60E-07 | 2.46 |
| *IRF1* | 9.30E-07 | 1.84 | *PPFIA2* | 3.96E-07 | 2.46 |
| *CD53* | 3.47E-08 | 1.86 | *FAM195B* | 7.02E-08 | 2.47 |
| *COL15A1* | 8.22E-08 | 1.86 | *CRISPLD1* | 1.06E-06 | 2.47 |
| *CCL2* | 1.43E-06 | 1.87 | *DUXA* | 3.22E-08 | 2.48 |
| *DIAPH3* | 1.63E-07 | 1.87 | *INHBE* | 3.59E-07 | 2.48 |
| *CASP5* | 6.47E-08 | 1.88 | *6-Sep* | 1.16E-06 | 2.48 |
| *CASP4* | 6.47E-08 | 1.88 | *PKD1L1* | 1.35E-07 | 2.48 |
| *IGHV3-49* | 4.24E-07 | 1.89 | *DBP* | 1.78E-08 | 2.49 |
| *SLC29A3* | 2.79E-08 | 1.89 | *PEBP4* | 7.94E-07 | 2.50 |
| *DUXA* | 2.52E-07 | 1.90 | *PPP2R3A* | 7.08E-07 | 2.51 |
| *C1RL* | 9.14E-08 | 1.91 | *AOC3* | 1.58E-07 | 2.51 |
| *TNS1* | 1.08E-08 | 1.91 | *C7orf72* | 2.20E-09 | 2.52 |
| *CTSB* | 1.04E-07 | 1.91 | *LZTR1* | 4.60E-07 | 2.52 |
| *FBXO5* | 7.00E-08 | 1.91 | *FAM135A* | 1.53E-07 | 2.52 |
| *IGHM* | 5.20E-08 | 1.91 | *ARIH2OS* | 4.86E-07 | 2.52 |
| *EPPIN* | 3.28E-07 | 1.92 | *ARIH2* | 4.86E-07 | 2.52 |
| *NAT8* | 2.31E-07 | 1.92 | *SHPK* | 9.44E-08 | 2.53 |
| *DHX58* | 1.02E-07 | 1.92 | *DSCAML1* | 9.40E-09 | 2.53 |
| *DESI1* | 1.24E-07 | 1.92 | *CSF3* | 1.44E-06 | 2.53 |
| *RBM10* | 2.23E-08 | 1.93 | *SMIM3* | 1.48E-07 | 2.53 |
| *CNOT1* | 2.23E-08 | 1.93 | *MAP3K4* | 8.81E-08 | 2.53 |
| *LAP3* | 2.16E-08 | 1.94 | *SASH1* | 3.01E-07 | 2.53 |
| *C4A* | 1.23E-07 | 1.94 | *NRN1* | 1.17E-09 | 2.54 |
| *C4B* | 1.23E-07 | 1.94 | *ZNF518B* | 1.17E-09 | 2.54 |
| *CCDC111* | 1.98E-07 | 1.94 | *CREG1* | 4.35E-08 | 2.54 |
| *LAPTM5* | 4.15E-08 | 1.95 | *RBL1* | 8.47E-07 | 2.55 |
| *IDO1* | 2.50E-08 | 1.95 | *FADS1* | 3.71E-07 | 2.55 |
| *NR4A1* | 1.80E-08 | 1.95 | *B2M* | 2.82E-09 | 2.56 |
| *MAP3K8* | 1.41E-08 | 1.96 | *SLFN14* | 2.82E-09 | 2.56 |
| *SLC26A4* | 1.76E-06 | 1.96 | *SLC17A9* | 4.78E-08 | 2.56 |
| *NTRK2* | 4.22E-09 | 1.96 | *OR52A5* | 1.63E-07 | 2.56 |
| *GUSB* | 1.15E-07 | 1.96 | *GRIN1* | 2.33E-07 | 2.57 |
| *ITGB7* | 1.20E-06 | 1.96 | *MYH7* | 1.16E-07 | 2.59 |
| *KIF14* | 2.14E-06 | 1.97 | *PTCHD2* | 3.11E-07 | 2.59 |
| *IGHA1* | 3.18E-08 | 1.97 | *OR12D2* | 1.34E-07 | 2.61 |
| *IGHA2* | 3.18E-08 | 1.97 | *SH3BGR* | 1.41E-06 | 2.62 |
| *EZH2* | 2.16E-07 | 1.97 | *DPY30* | 3.95E-07 | 2.62 |
| *PHLDB1* | 1.78E-06 | 1.98 | *HSF4* | 4.87E-08 | 2.62 |
| *NOD1* | 2.53E-07 | 1.98 | *POLR2J* | 3.52E-07 | 2.62 |
| *LY86* | 3.10E-08 | 1.98 | *TTI2* | 9.81E-07 | 2.64 |
| *P2RY10* | 2.20E-07 | 1.98 | *POLH* | 1.88E-08 | 2.64 |
| *GLIPR2* | 1.00E-07 | 1.99 | *SNX29P2* | 1.01E-07 | 2.65 |
| *CD200R1L* | 9.27E-07 | 1.99 | *SNX29* | 1.01E-07 | 2.65 |
| *CD200R1* | 9.27E-07 | 1.99 | *DDAH1* | 9.48E-07 | 2.65 |
| *TNFSF13B* | 7.17E-08 | 1.99 | *CDSN* | 6.85E-07 | 2.65 |
| *CD96* | 2.75E-08 | 1.99 | *MYO5B* | 1.77E-08 | 2.66 |
| *ATP8B4* | 5.09E-07 | 2.00 | *INSR* | 4.73E-08 | 2.67 |
| *PLAU* | 1.83E-07 | 2.00 | *LMBRD2* | 3.87E-07 | 2.69 |
| *ITGB2* | 6.09E-09 | 2.01 | *CENPQ* | 1.61E-06 | 2.70 |
| *HLA-DRA* | 5.65E-07 | 2.01 | *EIF2S1* | 1.33E-08 | 2.70 |
| *IGHV4-4* | 7.52E-09 | 2.02 | *NRXN1* | 1.42E-08 | 2.71 |
| *IGHV4-39* | 7.52E-09 | 2.02 | *CTRB2* | 4.25E-07 | 2.71 |
| *IGHV4-28* | 7.52E-09 | 2.02 | *NR4A1* | 1.39E-09 | 2.71 |
| *IGHV4-61* | 7.52E-09 | 2.02 | *NOG* | 9.91E-07 | 2.71 |
| *IGHV6-1* | 7.52E-09 | 2.02 | *STAR* | 2.95E-06 | 2.71 |
| *IGHV4-31* | 7.52E-09 | 2.02 | *HDHD3* | 2.27E-07 | 2.72 |
| *IGHV4-59* | 7.52E-09 | 2.02 | *ELMO2* | 1.93E-06 | 2.74 |
| *IGHV4-34* | 7.52E-09 | 2.02 | *NFAT5* | 1.93E-06 | 2.74 |
| *FAM18A* | 3.69E-08 | 2.02 | *CELF3* | 1.93E-06 | 2.74 |
| *IKBKE* | 1.31E-07 | 2.03 | *ELMO1* | 1.93E-06 | 2.74 |
| *PTPN22* | 1.26E-08 | 2.04 | *SLC6A5* | 2.97E-08 | 2.74 |
| *UBE2C* | 1.12E-07 | 2.04 | *TRBV21OR9-2* | 4.30E-08 | 2.74 |
| *PTGDR* | 2.79E-06 | 2.04 | *CRYZ* | 2.71E-09 | 2.75 |
| *COX7A2* | 1.28E-07 | 2.05 | *IL4R* | 4.49E-08 | 2.76 |
| *RPL14* | 1.28E-07 | 2.05 | *TCP11L1* | 7.55E-08 | 2.78 |
| *CTSD* | 1.28E-07 | 2.05 | *SEL1L* | 1.92E-09 | 2.78 |
| *PIP* | 1.03E-06 | 2.05 | *COL4A1* | 1.92E-09 | 2.78 |
| *NCF4* | 5.15E-08 | 2.05 | *PITPNM2* | 2.86E-08 | 2.79 |
| *HLA-DMA* | 1.54E-08 | 2.05 | *IGLL1* | 1.77E-08 | 2.80 |
| *FCRLA* | 4.19E-07 | 2.06 | *IGLC7* | 1.77E-08 | 2.80 |
| *ADAMTS6* | 2.96E-07 | 2.07 | *IGLC2* | 1.77E-08 | 2.80 |
| *ABI3* | 4.90E-08 | 2.07 | *IGLL5* | 1.77E-08 | 2.80 |
| *SLC15A3* | 7.02E-08 | 2.08 | *IGLC1* | 1.77E-08 | 2.80 |
| *HN1* | 5.22E-09 | 2.08 | *IGLC3* | 1.77E-08 | 2.80 |
| *FBLN5* | 1.39E-06 | 2.08 | *PAK3* | 4.64E-07 | 2.80 |
| *RASGRP1* | 1.06E-08 | 2.08 | *EZH2* | 1.39E-08 | 2.81 |
| *DENND1C* | 1.64E-08 | 2.08 | *TBX20* | 4.80E-10 | 2.81 |
| *CD27* | 5.48E-09 | 2.08 | *SERPINB13* | 2.48E-06 | 2.81 |
| *PKM* | 1.66E-08 | 2.08 | *MMD* | 4.06E-08 | 2.81 |
| *MYO5B* | 1.13E-07 | 2.09 | *OR1E2* | 2.85E-06 | 2.82 |
| *B2M* | 1.36E-08 | 2.09 | *OR1E1* | 2.85E-06 | 2.82 |
| *SLFN14* | 1.36E-08 | 2.09 | *DVL3* | 2.97E-07 | 2.82 |
| *ATP1A4* | 4.54E-07 | 2.10 | *LRRC8C* | 1.30E-07 | 2.82 |
| *TF* | 5.25E-09 | 2.10 | *FAM103A1* | 8.30E-09 | 2.82 |
| *C1R* | 4.79E-08 | 2.11 | *CYP7A1* | 8.48E-09 | 2.83 |
| *KCNK7* | 1.55E-07 | 2.11 | *GJB1* | 3.75E-09 | 2.84 |
| *GGCX* | 2.79E-08 | 2.12 | *BMS1* | 3.49E-08 | 2.84 |
| *ONECUT1* | 4.35E-08 | 2.12 | *IGKC* | 1.08E-09 | 2.84 |
| *MPEG1* | 1.76E-06 | 2.12 | *GGCX* | 2.79E-09 | 2.84 |
| *CORO1A* | 4.55E-08 | 2.12 | *MS4A4A* | 1.02E-07 | 2.85 |
| *CKS2* | 7.46E-09 | 2.13 | *B3GNT2* | 2.03E-07 | 2.86 |
| *CKB* | 1.17E-07 | 2.13 | *FOXF2* | 2.12E-09 | 2.86 |
| *TEAD2* | 2.52E-06 | 2.14 | *CLUL1* | 7.50E-08 | 2.86 |
| *TEAD3* | 2.52E-06 | 2.14 | *GPC3* | 1.77E-09 | 2.88 |
| *CD37* | 2.52E-06 | 2.14 | *SERPINE1* | 2.49E-09 | 2.89 |
| *TEAD4* | 2.52E-06 | 2.14 | *PTGER2* | 3.77E-09 | 2.89 |
| *AURKB* | 7.30E-07 | 2.14 | *ATP12A* | 6.45E-08 | 2.90 |
| *TMEM40* | 1.01E-07 | 2.14 | *FXYD1* | 2.38E-09 | 2.91 |
| *CTBS* | 9.57E-09 | 2.15 | *PTN* | 8.97E-08 | 2.91 |
| *TIMP1* | 1.98E-07 | 2.15 | *BTBD7* | 4.27E-09 | 2.91 |
| *PSMB10* | 3.16E-07 | 2.16 | *JUN* | 3.14E-07 | 2.92 |
| *BID* | 3.38E-08 | 2.16 | *HOMER2* | 3.41E-07 | 2.92 |
| *SH3BP2* | 1.01E-06 | 2.16 | *SPACA4* | 1.57E-08 | 2.93 |
| *SLA* | 9.17E-07 | 2.16 | *OR7D4* | 2.94E-08 | 2.93 |
| *APITD1* | 4.24E-07 | 2.16 | *SLC43A2* | 6.61E-07 | 2.95 |
| *SLC7A5* | 2.15E-08 | 2.16 | *TMEM132E* | 6.45E-07 | 2.96 |
| *BUB1* | 8.88E-07 | 2.17 | *GOLGB1* | 5.46E-07 | 2.98 |
| *KIAA0101* | 2.57E-08 | 2.17 | *KL* | 2.53E-07 | 2.98 |
| *UHRF1BP1* | 1.36E-08 | 2.17 | *ZCCHC10* | 1.42E-07 | 2.98 |
| *RNASE6* | 1.99E-08 | 2.18 | *RGL4* | 1.67E-06 | 2.98 |
| *FTL* | 5.36E-08 | 2.18 | *RPA3* | 4.57E-08 | 2.99 |
| *ACSL1* | 2.71E-06 | 2.18 | *NIPAL4* | 1.19E-06 | 3.00 |
| *EDEM1* | 3.19E-08 | 2.18 | *CNP* | 1.43E-08 | 3.01 |
| *MKI67* | 1.12E-07 | 2.18 | *PLEKHG6* | 1.13E-09 | 3.01 |
| *PLEKHO2* | 4.20E-09 | 2.20 | *BCAP31* | 1.69E-07 | 3.02 |
| *CD274* | 3.73E-07 | 2.22 | *SPC25* | 3.40E-09 | 3.03 |
| *TRAT1* | 1.01E-08 | 2.23 | *INHBA* | 1.12E-08 | 3.03 |
| *PPARGC1B* | 4.15E-08 | 2.23 | *FAM123C* | 8.89E-08 | 3.05 |
| *TBX20* | 2.81E-09 | 2.24 | *RPL22* | 6.10E-07 | 3.05 |
| *CLCN7* | 1.92E-09 | 2.25 | *HSP90B1* | 4.82E-08 | 3.07 |
| *IRF7* | 6.60E-09 | 2.25 | *C19orf54* | 4.82E-08 | 3.07 |
| *TFEC* | 5.75E-07 | 2.25 | *CXCL16* | 4.82E-08 | 3.07 |
| *RUNDC3B* | 2.09E-07 | 2.26 | *AMDHD1* | 2.38E-06 | 3.08 |
| *CLU* | 7.85E-08 | 2.26 | *RLTPR* | 6.91E-09 | 3.09 |
| *IL1RN* | 1.81E-08 | 2.26 | *TMEM202* | 8.39E-08 | 3.09 |
| *LRRC37B* | 2.36E-06 | 2.28 | *CSDE1* | 7.72E-08 | 3.10 |
| *FYN* | 1.10E-08 | 2.28 | *PHLDB1* | 5.66E-08 | 3.10 |
| *COL1A1* | 2.01E-09 | 2.28 | *TRIML2* | 2.27E-09 | 3.10 |
| *GTF3C4* | 2.01E-09 | 2.28 | *SACS* | 7.38E-07 | 3.10 |
| *PTGER2* | 2.40E-08 | 2.28 | *OR5W2* | 2.02E-06 | 3.11 |
| *LTB* | 6.29E-07 | 2.31 | *MRS2* | 1.46E-07 | 3.11 |
| *IGHG1* | 6.09E-09 | 2.31 | *ZNF709* | 7.04E-07 | 3.12 |
| *IGHG2* | 6.09E-09 | 2.31 | *ZNF470* | 7.04E-07 | 3.12 |
| *IGHG3* | 6.09E-09 | 2.31 | *GFOD2* | 7.68E-08 | 3.12 |
| *IGHG4* | 6.09E-09 | 2.31 | *CEP78* | 1.46E-07 | 3.13 |
| *NFKBIE* | 1.62E-08 | 2.31 | *NTRK2* | 1.06E-10 | 3.13 |
| *PRAM1* | 6.16E-08 | 2.33 | *MFSD5* | 1.90E-07 | 3.15 |
| *GBP4* | 9.26E-08 | 2.34 | *VDAC3* | 6.74E-08 | 3.17 |
| *GBP7* | 9.26E-08 | 2.34 | *SLC6A13* | 1.66E-07 | 3.17 |
| *GNA11* | 2.56E-08 | 2.35 | *CERS3* | 1.33E-07 | 3.18 |
| *HLA-A* | 1.29E-09 | 2.36 | *HTR2A* | 1.73E-06 | 3.18 |
| *IGSF6* | 1.82E-08 | 2.38 | *MMP2* | 1.51E-09 | 3.18 |
| *IL23R* | 2.10E-07 | 2.39 | *LTBR* | 4.23E-07 | 3.18 |
| *ACAP1* | 1.34E-08 | 2.40 | *ABCC5* | 1.61E-07 | 3.18 |
| *IKZF3* | 2.18E-07 | 2.40 | *HNRNPA1L2* | 4.88E-09 | 3.21 |
| *ADCYAP1* | 6.49E-07 | 2.41 | *HNRNPA1* | 4.88E-09 | 3.21 |
| *C16orf72* | 8.68E-07 | 2.41 | *AK2* | 8.24E-08 | 3.21 |
| *PIK3CD* | 6.25E-08 | 2.41 | *SPRY2* | 1.48E-07 | 3.25 |
| *IL18BP* | 4.70E-08 | 2.41 | *TMX1* | 1.87E-08 | 3.25 |
| *LDHA* | 3.87E-09 | 2.41 | *ELAVL4* | 5.76E-07 | 3.26 |
| *ELF2* | 2.74E-08 | 2.42 | *PPIL4* | 1.29E-08 | 3.27 |
| *VCAN* | 1.41E-07 | 2.42 | *DCUN1D1* | 4.49E-09 | 3.28 |
| *PTPRC* | 1.44E-08 | 2.42 | *XIRP1* | 4.49E-09 | 3.28 |
| *RASAL3* | 8.76E-08 | 2.43 | *C17orf104* | 3.37E-07 | 3.29 |
| *APOBEC1* | 5.20E-07 | 2.43 | *CHN1* | 1.68E-08 | 3.29 |
| *ATP6V0D2* | 2.13E-09 | 2.43 | *ITPR2* | 1.39E-08 | 3.30 |
| *TNFAIP6* | 3.82E-09 | 2.43 | *CDK16* | 1.92E-06 | 3.31 |
| *RASSF4* | 4.01E-08 | 2.45 | *USP22* | 1.15E-06 | 3.34 |
| *SAMD3* | 1.94E-08 | 2.45 | *NAA25* | 5.59E-08 | 3.35 |
| *IRF8* | 8.96E-09 | 2.47 | *TTLL5* | 1.46E-08 | 3.36 |
| *RNF19B* | 1.36E-08 | 2.47 | *LIPF* | 1.29E-06 | 3.36 |
| *LCP1* | 9.11E-08 | 2.47 | *TDRD12* | 1.09E-07 | 3.36 |
| *CHN1* | 1.53E-07 | 2.48 | *SLC2A12* | 5.42E-07 | 3.37 |
| *HAVCR1* | 2.06E-07 | 2.48 | *IDI1* | 1.37E-07 | 3.39 |
| *HCK* | 2.26E-07 | 2.49 | *ABHD12B* | 6.96E-07 | 3.40 |
| *GZMB* | 5.71E-09 | 2.49 | *GIT1* | 8.04E-07 | 3.40 |
| *PSTPIP2* | 1.35E-08 | 2.50 | *NAE1* | 9.29E-07 | 3.40 |
| *GPR18* | 1.06E-06 | 2.51 | *TSC22D3* | 1.64E-07 | 3.41 |
| *CD79B* | 1.28E-08 | 2.51 | *C16orf72* | 6.02E-08 | 3.41 |
| *IRF5* | 6.40E-08 | 2.51 | *EIF4E1B* | 2.63E-07 | 3.42 |
| *ATAD2* | 1.79E-07 | 2.51 | *GZMK* | 2.87E-08 | 3.44 |
| *LIPG* | 4.92E-07 | 2.53 | *CCDC169-SOHLH2* | 1.06E-07 | 3.46 |
| *ZAP70* | 4.01E-07 | 2.53 | *SOHLH2* | 1.06E-07 | 3.46 |
| *HTRA4* | 1.54E-07 | 2.53 | *SLC39A12* | 1.69E-07 | 3.46 |
| *BIRC3* | 5.54E-09 | 2.53 | *GYS2* | 2.22E-07 | 3.47 |
| *FYB* | 9.17E-09 | 2.54 | *FBLN5* | 2.64E-08 | 3.48 |
| *HLA-C* | 1.77E-09 | 2.56 | *CDK5RAP2* | 2.19E-07 | 3.48 |
| *HLA-F* | 1.77E-09 | 2.56 | *CCDC54* | 4.22E-08 | 3.49 |
| *LCP2* | 3.86E-09 | 2.56 | *SLC38A5* | 3.54E-07 | 3.49 |
| *RGS1* | 8.86E-09 | 2.58 | *LHX1* | 2.12E-09 | 3.52 |
| *CD3D* | 6.24E-09 | 2.59 | *CYP39A1* | 2.12E-09 | 3.52 |
| *FABP4* | 1.49E-08 | 2.60 | *PDCD11* | 1.00E-07 | 3.54 |
| *CXCL13* | 8.25E-08 | 2.60 | *FAM167A* | 1.99E-06 | 3.55 |
| *AOAH* | 1.64E-07 | 2.61 | *FANCC* | 2.05E-07 | 3.55 |
| *TDRD12* | 7.65E-07 | 2.61 | *FGF5* | 6.89E-07 | 3.55 |
| *SH2D1A* | 1.21E-08 | 2.63 | *KBTBD10* | 6.64E-09 | 3.55 |
| *INPP4B* | 1.25E-07 | 2.64 | *HMGXB3* | 1.69E-07 | 3.55 |
| *DMBT1* | 2.01E-07 | 2.65 | *FAM53C* | 9.14E-08 | 3.56 |
| *NCR1* | 1.85E-06 | 2.66 | *ADCY2* | 8.98E-08 | 3.59 |
| *BCL2A1* | 6.72E-10 | 2.66 | *LPGAT1* | 6.62E-08 | 3.61 |
| *TAGAP* | 6.05E-10 | 2.68 | *MPZL2* | 6.62E-08 | 3.61 |
| *GZMK* | 1.98E-07 | 2.68 | *FGD1* | 6.62E-08 | 3.61 |
| *AKR1E2* | 6.80E-07 | 2.68 | *DNAJB6* | 6.62E-08 | 3.61 |
| *MAL* | 1.95E-09 | 2.70 | *SI* | 1.23E-06 | 3.62 |
| *TRAF3IP3* | 8.03E-09 | 2.70 | *ASB9* | 2.77E-09 | 3.66 |
| *TACC3* | 2.52E-09 | 2.73 | *DIP2C* | 1.86E-07 | 3.67 |
| *CYBB* | 2.62E-06 | 2.73 | *LRRC37B* | 6.03E-08 | 3.67 |
| *RRM2* | 1.10E-07 | 2.73 | *CYP2C19* | 1.27E-07 | 3.69 |
| *SRC* | 2.92E-08 | 2.74 | *CYP2C9* | 1.27E-07 | 3.69 |
| *SNX10* | 1.53E-08 | 2.74 | *CYP2C8* | 1.27E-07 | 3.69 |
| *HPR* | 1.39E-08 | 2.76 | *PRICKLE4* | 8.85E-07 | 3.75 |
| *HP* | 1.39E-08 | 2.76 | *ADCYAP1* | 2.07E-08 | 3.76 |
| *BST1* | 3.43E-07 | 2.76 | *EHBP1* | 4.52E-08 | 3.77 |
| *CTSK* | 1.81E-09 | 2.77 | *KDELC2* | 1.03E-07 | 3.83 |
| *EPSTI1* | 2.45E-08 | 2.77 | *GLP2R* | 3.69E-08 | 3.87 |
| *CP* | 1.08E-09 | 2.79 | *RUNDC3B* | 2.98E-09 | 3.89 |
| *THEMIS* | 2.54E-07 | 2.79 | *FER1L5* | 1.08E-06 | 3.92 |
| *PSMB9* | 5.31E-10 | 2.82 | *AKD1* | 1.37E-09 | 3.97 |
| *PRKCB* | 1.10E-09 | 2.83 | *PNLIP* | 1.75E-07 | 4.00 |
| *AMICA1* | 2.51E-08 | 2.84 | *NPIPP1* | 1.57E-07 | 4.01 |
| *CD3G* | 1.66E-09 | 2.85 | *PKD1* | 1.57E-07 | 4.01 |
| *LAG3* | 2.90E-08 | 2.88 | *RUNX3* | 9.30E-08 | 4.06 |
| *TRGV8* | 9.00E-09 | 2.88 | *USP12* | 3.32E-07 | 4.08 |
| *TRGV5* | 9.00E-09 | 2.88 | *IFNAR1* | 3.38E-07 | 4.19 |
| *TRGC1* | 9.00E-09 | 2.88 | *SSR1* | 4.06E-10 | 4.37 |
| *TRGC2* | 9.00E-09 | 2.88 | *AKR1E2* | 1.42E-08 | 4.42 |
| *TRGV3* | 9.00E-09 | 2.88 | *SLC17A6* | 1.03E-08 | 4.53 |
| *TRGV4* | 9.00E-09 | 2.88 | *ZP2* | 1.37E-07 | 4.56 |
| *TRGV1* | 9.00E-09 | 2.88 | *THPO* | 2.64E-08 | 4.57 |
| *TRGV2* | 9.00E-09 | 2.88 | *VPS28* | 7.30E-10 | 4.57 |
| *THPO* | 9.22E-07 | 2.88 | *SLC44A5* | 1.24E-08 | 4.60 |
| *SPHK1* | 9.48E-09 | 2.89 | *OLFML2B* | 5.86E-11 | 4.65 |
| *ATP12A* | 6.71E-08 | 2.89 | *STT3B* | 2.69E-08 | 4.71 |
| *SLC44A5* | 4.42E-07 | 2.90 | *PDE6H* | 9.40E-09 | 4.72 |
| *TRIM69* | 1.33E-07 | 2.91 | *MYRIP* | 4.28E-09 | 4.89 |
| *NPIPP1* | 1.82E-06 | 2.91 | *PAX6* | 3.30E-10 | 5.03 |
| *PKD1* | 1.82E-06 | 2.91 |  |  |  |
| *MARCO* | 1.52E-09 | 2.92 |  |  |  |
| *CXCR6* | 1.27E-07 | 2.93 |  |  |  |
| *PNLIP* | 1.80E-06 | 2.94 |  |  |  |
| *MAP4K1* | 5.17E-10 | 2.98 |  |  |  |
| *KYNU* | 8.95E-08 | 3.06 |  |  |  |
| *GIMAP5* | 2.51E-09 | 3.07 |  |  |  |
| *C1orf162* | 5.68E-09 | 3.14 |  |  |  |
| *LCK* | 1.41E-08 | 3.17 |  |  |  |
| *TAP1* | 1.72E-10 | 3.18 |  |  |  |
| *CD68* | 6.81E-10 | 3.19 |  |  |  |
| *CCL19* | 6.31E-07 | 3.22 |  |  |  |
| *BDKRB1* | 1.56E-08 | 3.23 |  |  |  |
| *HCST* | 5.40E-10 | 3.27 |  |  |  |
| *SLC17A6* | 1.24E-07 | 3.29 |  |  |  |
| *PLA2G2A* | 5.07E-10 | 3.29 |  |  |  |
| *ICOS* | 5.25E-08 | 3.30 |  |  |  |
| *TNF* | 6.69E-10 | 3.31 |  |  |  |
| *CCL5* | 2.94E-10 | 3.31 |  |  |  |
| *SLAMF7* | 4.09E-10 | 3.35 |  |  |  |
| *KMO* | 3.75E-09 | 3.36 |  |  |  |
| *CD2* | 7.25E-10 | 3.39 |  |  |  |
| *CLEC4E* | 1.30E-10 | 3.39 |  |  |  |
| *C1QC* | 6.45E-10 | 3.40 |  |  |  |
| *EGLN3* | 2.75E-10 | 3.42 |  |  |  |
| *FPR1* | 1.02E-08 | 3.47 |  |  |  |
| *NCF1* | 6.91E-10 | 3.47 |  |  |  |
| *USP12* | 1.08E-06 | 3.49 |  |  |  |
| *TLR2* | 3.18E-10 | 3.52 |  |  |  |
| *FCGR2B* | 5.63E-07 | 3.57 |  |  |  |
| *FCGR2A* | 5.63E-07 | 3.57 |  |  |  |
| *TRAC* | 4.05E-10 | 3.58 |  |  |  |
| *TRBC2* | 6.14E-10 | 3.59 |  |  |  |
| *GPR171* | 7.58E-09 | 3.60 |  |  |  |
| *CD5* | 2.14E-09 | 3.70 |  |  |  |
| *XDH* | 2.68E-07 | 3.77 |  |  |  |
| *C1QB* | 3.44E-09 | 3.79 |  |  |  |
| *DCSTAMP* | 1.71E-07 | 3.83 |  |  |  |
| *PRF1* | 3.33E-09 | 3.89 |  |  |  |
| *CD38* | 9.76E-11 | 3.95 |  |  |  |
| *CXCL10* | 4.96E-10 | 3.98 |  |  |  |
| *STAT1* | 1.59E-10 | 4.10 |  |  |  |
| *CCL4L1* | 1.56E-11 | 4.23 |  |  |  |
| *CCL4* | 1.56E-11 | 4.23 |  |  |  |
| *CCL4L2* | 1.56E-11 | 4.23 |  |  |  |
| *BPIFA1* | 1.67E-08 | 4.24 |  |  |  |
| *ORM1* | 3.47E-11 | 4.34 |  |  |  |
| *ORM2* | 3.47E-11 | 4.34 |  |  |  |
| *GBP5* | 7.07E-10 | 4.63 |  |  |  |
| *CXCL9* | 8.28E-11 | 5.12 |  |  |  |
| *SAA2* | 5.16E-12 | 5.13 |  |  |  |
| *SAA1* | 5.16E-12 | 5.13 |  |  |  |
| *ARG1* | 3.80E-09 | 5.18 |  |  |  |
| *FAM26F* | 6.01E-12 | 5.47 |  |  |  |
| *MMP1* | 3.12E-09 | 5.57 |  |  |  |
| *GBP3* | 5.20E-10 | 5.72 |  |  |  |
| *GBP1* | 5.20E-10 | 5.72 |  |  |  |
| *GZMA* | 2.20E-12 | 5.77 |  |  |  |
| *GVINP1* | 1.96E-10 | 5.87 |  |  |  |
