# Supplementary material for: Early innate immunity determines outcome of Mycobacterium tuberculosis pulmonary infection in rabbits
Source: Cell Commun Signal. 2013 Aug 19;11:60. doi: 10.1186/1478-811X-11-60 (PMC3765177; doi:10.1186/1478-811X-11-60)
Supplement: Additional file 2: Table S3 — Validation of microarray gene expression in the lungs of Mtb-infected rabbits at 3 hours by qRT-PCR. [file 1478-811X-11-60-S2.doc]

|  | ***CDC1551*** | | | | | ***HN878*** | | | |  |
| --- | --- | --- | --- | --- | --- | --- | --- | --- | --- | --- |
| **Gene** | **Array*** | **qPCR*** | | | | **Array*** | | **qPCR*** | |  |
| **Symbol** |  | ***Avg*** | | ***sd*** | |  | | ***Avg*** | ***sd*** | ***p value***^†^ |
| *TNF* | -0.52 | -1.42 | | 0.27 | | 3.31 | | 1.63 | 0.44 | 0.021501 |
| *IL4R* | 2.76 | 2.55 | | 0.16 | | 0.87 | | 3.48 | 0.15 | 0.003198 |
| *CD36* | 0.57 | 0.28 | | 0.05 | | -1.52 | | -2.68 | 0.80 | 0.000001 |
| *CXCL10* | -0.73 | -2.95 | | 0.33 | | 3.98 | | 1.90 | 0.23 | 0.031926 |
| *IL1A* | -2.00 | -2.16 | | 0.25 | | 0.66 | | 0.44 | 0.14 | 0.022345 |
| *CAV1* | -0.02 | 0.35 | | 0.37 | | -2.21 | | -3.60 | 0.49 | 0.067308 |
| *TGFB2* | -0.24 | -1.03 | | 0.15 | | -1.38 | | -2.68 | 0.17 | 0.023706 |
| *SPP1* | -3.28 | -3.59 | | 0.19 | | 1.50 | | 1.09 | 0.01 | 0.000044 |
| *CCL4* | -0.54 | -1.47 | | 0.78 | | 4.23 | | 2.79 | 0.67 | 0.030037 |
| *IL18* | -0.64 | -1.20 | | 0.48 | | -1.20 | | -3.38 | 0.43 | 0.099643 |
| *CCL2* | -1.46 | -2.14 | | 0.00 | | 1.87 | | 0.52 | 0.63 | 0.030235 |
| *IRF5* | 0.30 | -0.13 | | 0.79 | | 2.50 | | 1.53 | 0.30 | 0.011415 |
| *CD38* | 0.57 | 0.76 | | 0.62 | | 3.95 | | 3.99 | 0.46 | 0.038813 |
| *STAT1* | 0.26 | -0.50 | | 0.00 | | 4.1 | | 1.71 | 0.02 | 0.000176 |
| * log_2_ scale | |  | |  | |  | |  |  |  |
| ^†^ comparison between CDC1551 and HN878 qPCR data | | | | | | | | |  |  |
| Avg- average | | |  | |  | |  |  |  |  |
| sd-standard deviation | | | | |  | |  |  |  |  |

**Supplementary Table S3.** Validation of microarray gene expression in the lungs of Mtb-infected rabbits at three hours by qRT-PCR
