# Supplementary material for: Early innate immunity determines outcome of Mycobacterium tuberculosis pulmonary infection in rabbits
Source: Cell Commun Signal. 2013 Aug 19;11:60. doi: 10.1186/1478-811X-11-60 (PMC3765177; doi:10.1186/1478-811X-11-60)
Supplement: Additional file 3: Table S4 — List of SDEG involved in inflammatory response in Mtb-infected rabbit lungs at 3 hours. [file 1478-811X-11-60-S3.doc]

**Supplementary Table S4.** List of SDEG involved in inflammatory response in Mtb-infected rabbit lungs at three hours

| **Gene Symbol** | **Gene Name** | **Location** | **Log_2_ ratio**  **HN878 CDC1551** | | **Human Gene ID** |
| --- | --- | --- | --- | --- | --- |
| **Cytokines/Chemokines** |  |  |  |  |  |
| *CCL18* | chemokine (C-C motif) ligand 18 | Extracellular Space | 2.01 | 0.13 | 6362 |
| *CCL19* | chemokine (C-C motif) ligand 19 | Extracellular Space | 3.22 | 1.06 | 6363 |
| *CCL2* | chemokine (C-C motif) ligand 2 | Extracellular Space | 1.87 | -1.46 | 6347 |
| *CCL20* | chemokine (C-C motif) ligand 20 | Extracellular Space | -0.70 | -2.05 | 6364 |
| *CCL21* | chemokine (C-C motif) ligand 21 | Extracellular Space | 0.18 | 1.75 | 6366 |
| *CCL3* | chemokine (C-C motif) ligand 3 | Extracellular Space | 2.01 | 0.13 | 6348 |
| *CCL3L1/CCL3L3* | chemokine (C-C motif) ligand 3-like 1 | Extracellular Space | 2.01 | 0.13 | 6349|414062 |
| *CCL4* | chemokine (C-C motif) ligand 4 | Extracellular Space | 4.23 | -0.54 | 6351 |
| *CCL5* | chemokine (C-C motif) ligand 5 | Extracellular Space | 3.31 | -0.35 | 6352 |
| *CSF2* | colony stimulating factor 2 (granulocyte-macrophage) | Extracellular Space | -1.92 | -1.44 | 1437 |
| *CSF3* | colony stimulating factor 3 (granulocyte) | Extracellular Space | 0.19 | 2.53 | 1440 |
| *CTF1* | cardiotrophin 1 | Extracellular Space | 0.19 | 1.99 | 1489 |
| *CXCL1* | chemokine (C-X-C motif) ligand 1 | Extracellular Space | -0.25 | -2.13 | 2919 |
| *CXCL10* | chemokine (C-X-C motif) ligand 10 | Extracellular Space | 3.98 | -0.73 | 3627 |
| *CXCL13* | chemokine (C-X-C motif) ligand 13 | Extracellular Space | 2.60 | 1.52 | 10563 |
| *CXCL16* | chemokine (C-X-C motif) ligand 16 | Extracellular Space | 0.88 | 3.07 | 58191 |
| *CXCL2* | chemokine (C-X-C motif) ligand 2 | Extracellular Space | -0.25 | -2.13 | 2920 |
| *CXCL3* | chemokine (C-X-C motif) ligand 3 | Extracellular Space | -0.25 | -2.13 | 2921 |
| *CXCL5* | chemokine (C-X-C motif) ligand 5 | Extracellular Space | 1.57 | -1.51 | 6374 |
| *CXCL6* | chemokine (C-X-C motif) ligand 6 | Extracellular Space | 1.57 | -1.51 | 6372 |
| *CXCL9* | chemokine (C-X-C motif) ligand 9 | Extracellular Space | 5.12 | 0.08 | 4283 |
| *EDN1* | endothelin 1 | Extracellular Space | 0.21 | 1.34 | 1906 |
| *IL15* | interleukin 15 | Extracellular Space | 1.30 | -0.35 | 3600 |
| *IL18* | interleukin 18 (interferon-gamma-inducing factor) | Extracellular Space | -1.20 | -0.64 | 3606 |
| *IL1A* | interleukin 1, alpha | Extracellular Space | 0.66 | -2.00 | 3552 |
| *IL1RN* | interleukin 1 receptor antagonist | Extracellular Space | 2.26 | -0.32 | 3557 |
| *IL22* | interleukin 22 | Extracellular Space | 1.23 | 0.02 | 50616 |
| *IL5* | interleukin 5 (colony-stimulating factor, eosinophil) | Extracellular Space | -0.31 | 1.79 | 3567 |
| *IL8* | interleukin 8 | Extracellular Space | 1.59 | -0.99 | 3576 |
| *LTB* | lymphotoxin beta (TNF superfamily, member 3) | Extracellular Space | 2.31 | 0.68 | 4050 |
| *PF4* | platelet factor 4 | Extracellular Space | -0.36 | 1.18 | 5196 |
| *PRL* | prolactin | Extracellular Space | 1.27 | 1.93 | 5617 |
| *SPP1* | secreted phosphoprotein 1 | Extracellular Space | 1.50 | -3.28 | 6696 |
| *THPO* | thrombopoietin | Extracellular Space | 2.88 | 4.57 | 7066 |
| *TNF* | tumor necrosis factor | Extracellular Space | 3.31 | -0.52 | 7124 |
| *TNFSF13B* | tumor necrosis factor (ligand) superfamily, member 13b | Extracellular Space | 1.99 | -0.75 | 10673 |
|  |  |  |  |  |  |
| **Enzymes** |  |  |  |  |  |
| *BIRC3* | baculoviral IAP repeat containing 3 | Cytoplasm | 2.53 | -0.54 | 330 |
| *CD274* | CD274 molecule | Plasma Membrane | 2.22 | -0.94 | 29126 |
| *CD38* | CD38 molecule | Plasma Membrane | 3.95 | 0.57 | 952 |
| *CD44* | CD44 molecule (Indian blood group) | Plasma Membrane | 0.82 | -0.57 | 960 |
| *CYBB* | cytochrome b-245, beta polypeptide | Cytoplasm | 2.73 | -0.82 | 1536 |
| *DDX58* | DEAD (Asp-Glu-Ala-Asp) box polypeptide 58 | Cytoplasm | 1.25 | -0.17 | 23586 |
| *FN1* | fibronectin 1 | Extracellular Space | 0.33 | 1.54 | 2335 |
| *GNA11* | guanine nucleotide binding protein (G protein), alpha 11 | Plasma Membrane | 2.35 | 0.21 | 2767 |
| *GNAI2* | guanine nucleotide binding protein (G protein), alpha inhibiting activity polypeptide 2 | Plasma Membrane | -1.57 | -0.46 | 2771 |
| *GNAS* | GNAS complex locus | Plasma Membrane | -0.89 | 0.13 | 2778 |
| *GUCY1B3* | guanylate cyclase 1, soluble, beta 3 | Cytoplasm | 0.08 | 1.01 | 2983 |
| *HSP90AA1* | heat shock protein 90kDa alpha (cytosolic), class A member 1 | Cytoplasm | 1.07 | -0.38 | 3320 |
| *LAMB2* | laminin, beta 2 (laminin S) | Extracellular Space | 0.05 | 1.44 | 3913 |
| *MX1* | myxovirus (influenza virus) resistance 1, interferon-inducible protein p78 (mouse) | Nucleus | 1.13 | -0.18 | 4599 |
| *NCF1* | neutrophil cytosolic factor 1 | Cytoplasm | 3.47 | 1.63 | 653361 |
| *NOS3* | nitric oxide synthase 3 (endothelial cell) | Cytoplasm | -0.01 | 1.21 | 4846 |
| *OCLN* | occludin | Plasma Membrane | -1.19 | -0.11 | 1.01E+08 |
| *PDE5A* | phosphodiesterase 5A, cGMP-specific | Cytoplasm | -0.76 | 1.13 | 8654 |
| *PTGS2* | prostaglandin-endoperoxide synthase 2 (prostaglandin G/H synthase and cyclooxygenase) | Cytoplasm | 0.60 | -0.35 | 5743 |
| *RAC2* | ras-related C3 botulinum toxin substrate 2 (rho family, small GTP binding protein Rac2) | Cytoplasm | 1.79 | 0.30 | 5880 |
| *RHOB* | ras homolog family member B | Cytoplasm | 0.16 | 1.04 | 388 |
| *SMURF2* | SMAD specific E3 ubiquitin protein ligase 2 | Cytoplasm | -0.61 | 0.25 | 64750 |
| *SOD1* | superoxide dismutase 1, soluble | Cytoplasm | -1.63 | -0.74 | 6647 |
|  |  |  |  |  |  |
| **G-protein coupled receptors** | |  |  |  |  |
| *BDKRB1* | bradykinin receptor B1 | Plasma Membrane | 3.23 | 0.18 | 623 |
| *CHRM2* | cholinergic receptor, muscarinic 2 | Plasma Membrane | -0.14 | 1.18 | 1129 |
| *CXCR2* | chemokine (C-X-C motif) receptor 2 | Plasma Membrane | 1.05 | 0.07 | 3579 |
| *CXCR6* | chemokine (C-X-C motif) receptor 6 | Plasma Membrane | 2.93 | 0.74 | 10663 |
| *EDNRB* | endothelin receptor type B | Plasma Membrane | -1.01 | 1.63 | 1910 |
| *FPR1* | formyl peptide receptor 1 | Plasma Membrane | 3.47 | -0.37 | 2357 |
| *FPR2* | formyl peptide receptor 2 | Plasma Membrane | 3.01 | -0.27 | 2358 |
| *GPR17* | G protein-coupled receptor 17 | Plasma Membrane | 0.21 | 1.04 | 2840 |
| *HTR2A* | 5-hydroxytryptamine (serotonin) receptor 2A, G protein-coupled | Plasma Membrane | 0.46 | 3.18 | 3356 |
| *P2RY1* | purinergic receptor P2Y, G-protein coupled, 1 | Plasma Membrane | -1.19 | -0.13 | 5028 |
| *PTGDR* | prostaglandin D2 receptor (DP) | Plasma Membrane | 2.04 | 0.07 | 5729 |
| *PTGER2* | prostaglandin E receptor 2 (subtype EP2), 53kDa | Plasma Membrane | 2.28 | 2.89 | 5732 |
| *PTGER3* | prostaglandin E receptor 3 (subtype EP3) | Plasma Membrane | -0.52 | 1.04 | 5733 |
| *PTGER4* | prostaglandin E receptor 4 (subtype EP4) | Plasma Membrane | 1.64 | 0.50 | 5734 |
|  |  |  |  |  |  |
| **Growth factors** |  |  |  |  |  |
| *ANGPT1* | angiopoietin 1 | Extracellular Space | -0.66 | 1.13 | 284 |
| *FIGF* | c-fos induced growth factor (vascular endothelial growth factor D) | Extracellular Space | 0.02 | 1.88 | 2277 |
| *HGF* | hepatocyte growth factor (hepapoietin A; scatter factor) | Extracellular Space | -0.39 | 0.81 | 3082 |
| *INHBA* | inhibin, beta A | Extracellular Space | 1.54 | 3.03 | 3624 |
| *PDGFB* | platelet-derived growth factor beta polypeptide | Extracellular Space | 0.69 | 1.84 | 5155 |
| *TGFB2* | transforming growth factor, beta 2 | Extracellular Space | -1.38 | -0.24 | 7042 |
| *VEGFC* | vascular endothelial growth factor C | Extracellular Space | -0.41 | 1.17 | 7424 |
|  |  |  |  |  |  |
| **Kinases** |  |  |  |  |  |
| *BMPR2* | bone morphogenetic protein receptor, type II (serine/threonine kinase) | Plasma Membrane | -0.17 | 1.66 | 659 |
| *CDKN1A* | cyclin-dependent kinase inhibitor 1A (p21, Cip1) | Nucleus | -0.03 | -1.19 | 1026 |
| *CSF1R* | colony stimulating factor 1 receptor | Plasma Membrane | 1.85 | -0.21 | 1436 |
| *FLT3* | fms-related tyrosine kinase 3 | Plasma Membrane | 1.28 | 0.68 | 2322 |
| *FYN* | FYN oncogene related to SRC, FGR, YES | Plasma Membrane | 2.28 | 1.57 | 2534 |
| *GRK5* | G protein-coupled receptor kinase 5 | Plasma Membrane | -0.53 | 0.79 | 2869 |
| *GRK6* | G protein-coupled receptor kinase 6 | Plasma Membrane | -0.53 | 0.79 | 2870 |
| *GSK3B* | glycogen synthase kinase 3 beta | Nucleus | -1.38 | -0.17 | 2932 |
| *HCK* | hemopoietic cell kinase | Cytoplasm | 2.49 | 0.18 | 3055 |
| *IKBKE* | inhibitor of kappa light polypeptide gene enhancer in B-cells, kinase epsilon | Cytoplasm | 2.03 | -0.52 | 9641 |
| *JAK1* | Janus kinase 1 | Cytoplasm | -1.90 | -0.51 | 3716 |
| *JAK2* | Janus kinase 2 | Cytoplasm | 1.71 | -0.10 | 3717 |
| *LCK* | lymphocyte-specific protein tyrosine kinase | Cytoplasm | 3.17 | 1.14 | 3932 |
| *LYN* | v-yes-1 Yamaguchi sarcoma viral related oncogene homolog | Cytoplasm | 1.54 | 0.40 | 4067 |
| *MAP3K8* | mitogen-activated protein kinase kinase kinase 8 | Cytoplasm | 1.96 | 0.10 | 1326 |
| *MAPK1* | mitogen-activated protein kinase 1 | Cytoplasm | -1.56 | -0.25 | 5594 |
| *MYLK* | myosin light chain kinase | Cytoplasm | 0.15 | 1.35 | 4638 |
| *NCK1* | NCK adaptor protein 1 | Cytoplasm | -0.42 | 1.92 | 4690 |
| *PDGFRA* | platelet-derived growth factor receptor, alpha polypeptide | Plasma Membrane | 0.99 | 2.42 | 5156 |
| *PIK3CD* | phosphatidylinositol-4,5-bisphosphate 3-kinase, catalytic subunit delta | Cytoplasm | 2.41 | 0.23 | 5293 |
| *PRKCB* | protein kinase C, beta | Cytoplasm | 2.83 | 1.04 | 5579 |
| *PRKCD* | protein kinase C, delta | Cytoplasm | -0.24 | -1.13 | 5580 |
| *PRKCQ* | protein kinase C, theta | Cytoplasm | 1.80 | -0.03 | 5588 |
| *PRKG1* | protein kinase, cGMP-dependent, type I | Cytoplasm | -1.70 | -0.06 | 5592 |
| *SHC1* | SHC (Src homology 2 domain containing) transforming protein 1 | Cytoplasm | 0.06 | 1.23 | 6464 |
| *SPHK1* | sphingosine kinase 1 | Cytoplasm | 2.89 | -0.23 | 8877 |
| *SRC* | v-src sarcoma (Schmidt-Ruppin A-2) viral oncogene homolog (avian) | Cytoplasm | 2.74 | 0.65 | 6714 |
| *TEK* | TEK tyrosine kinase, endothelial | Plasma Membrane | -0.94 | 1.58 | 7010 |
| *ZAP70* | zeta-chain (TCR) associated protein kinase 70kDa | Plasma Membrane | 2.53 | 0.41 | 7535 |
|  |  |  |  |  |  |
| **Nuclear receptor** |  |  |  |  |  |
| *NR4A1* | nuclear receptor subfamily 4, group A, member 1 | Nucleus | 1.95 | 2.71 | 3164 |
|  |  |  |  |  |  |
| **Peptidases** |  |  |  |  |  |
| *CASP1* | caspase 1, apoptosis-related cysteine peptidase | Cytoplasm | 1.28 | -0.14 | 834 |
| *CASP4* | caspase 4, apoptosis-related cysteine peptidase | Cytoplasm | 1.88 | -0.05 | 837 |
| *CASP8* | caspase 8, apoptosis-related cysteine peptidase | Nucleus | 1.59 | 0.88 | 841 |
| *CFB* | complement factor B | Extracellular Space | 1.71 | -0.65 | 629 |
| *CTSB* | cathepsin B | Cytoplasm | 1.91 | -0.12 | 1508 |
| *CTSH* | cathepsin H | Cytoplasm | 0.83 | -0.62 | 1512 |
| *CTSS* | cathepsin S | Cytoplasm | 1.16 | -0.03 | 1520 |
| *F2* | coagulation factor II (thrombin) | Extracellular Space | -0.88 | 0.35 | 2147 |
| *GZMA* | granzyme A (granzyme 1, cytotoxic T-lymphocyte-associated serine esterase 3) | Cytoplasm | 5.77 | 0.68 | 3001 |
| *GZMB* | granzyme B (granzyme 2, cytotoxic T-lymphocyte-associated serine esterase 1) | Cytoplasm | 2.49 | -0.85 | 3002 |
| *MME* | membrane metallo-endopeptidase | Plasma Membrane | -1.83 | -0.56 | 4311 |
| *MMP1* | matrix metallopeptidase 1 (interstitial collagenase) | Extracellular Space | 5.57 | -0.59 | 4312 |
| *PDIA3* | protein disulfide isomerase family A, member 3 | Cytoplasm | 0.95 | 0.02 | 2923 |
| *PLAU* | plasminogen activator, urokinase | Extracellular Space | 2.00 | 0.32 | 5328 |
| *PSMB10* | proteasome (prosome, macropain) subunit, beta type, 10 | Cytoplasm | 2.16 | 0.55 | 5699 |
| *PSMB8* | proteasome (prosome, macropain) subunit, beta type, 8 (large multifunctional peptidase 7) | Cytoplasm | 1.76 | 0.27 | 5696 |
| *PSMB9* | proteasome (prosome, macropain) subunit, beta type, 9 (large multifunctional peptidase 2) | Cytoplasm | 2.82 | 0.47 | 5698 |
| *PSME2* | proteasome (prosome, macropain) activator subunit 2 (PA28 beta) | Cytoplasm | 1.63 | 0.09 | 5721 |
|  |  |  |  |  |  |
| **Phosphatases** |  |  |  |  |  |
| *PPP3CA* | protein phosphatase 3, catalytic subunit, alpha isozyme | Cytoplasm | -1.02 | -0.48 | 5530 |
| *PTEN* | phosphatase and tensin homolog | Cytoplasm | -0.47 | 0.71 | 5728 |
| *PTPN1* | protein tyrosine phosphatase, non-receptor type 1 | Cytoplasm | 1.45 | 0.33 | 5770 |
| *PTPN2* | protein tyrosine phosphatase, non-receptor type 2 | Cytoplasm | 1.28 | 0.01 | 5771 |
| *PTPRC* | protein tyrosine phosphatase, receptor type, C | Plasma Membrane | 2.42 | 0.27 | 5788 |
|  |  |  |  |  |  |
| **Transcription regulators** | |  |  |  |  |
| *BCL10* | B-cell CLL/lymphoma 10 | Cytoplasm | 1.15 | 0.30 | 8915 |
| *CIITA* | class II, major histocompatibility complex, transactivator | Nucleus | 1.70 | 0.04 | 4261 |
| *GATA3* | GATA binding protein 3 | Nucleus | 0.33 | 1.46 | 2625 |
| *HMGB1* | high mobility group box 1 | Nucleus | -0.41 | 0.46 | 3146 |
| *IRF1* | interferon regulatory factor 1 | Nucleus | 1.84 | -0.60 | 3659 |
| *IRF5* | interferon regulatory factor 5 | Nucleus | 2.51 | 0.30 | 3663 |
| *IRF7* | interferon regulatory factor 7 | Nucleus | 2.25 | 0.53 | 3665 |
| *IRF8* | interferon regulatory factor 8 | Nucleus | 2.47 | 0.89 | 3394 |
| *JUN* | jun proto-oncogene | Nucleus | 1.07 | 2.92 | 3725 |
| *MEF2C* | myocyte enhancer factor 2C | Nucleus | 0.86 | 2.39 | 4208 |
| *NFKB2* | nuclear factor of kappa light polypeptide gene enhancer in B-cells 2 (p49/p100) | Nucleus | 1.69 | 0.02 | 4791 |
| *NFKBIA* | nuclear factor of kappa light polypeptide gene enhancer in B-cells inhibitor, alpha | Cytoplasm | 0.50 | -0.43 | 4792 |
| *PYCARD* | PYD and CARD domain containing | Cytoplasm | 1.15 | 0.29 | 29108 |
| *STAT1* | signal transducer and activator of transcription 1, 91kDa | Nucleus | 4.10 | 0.26 | 6772 |
|  |  |  |  |  |  |
| **Transmembrane receptors** | |  |  |  |  |
| *B2M* | beta-2-microglobulin | Plasma Membrane | 2.09 | 2.56 | 567 |
| *CD14* | CD14 molecule | Plasma Membrane | 1.81 | 0.42 | 929 |
| *CD2* | CD2 molecule | Plasma Membrane | 3.39 | 0.12 | 914 |
| *CD27* | CD27 molecule | Plasma Membrane | 2.08 | 0.70 | 939 |
| *CD36* | CD36 molecule (thrombospondin receptor) | Plasma Membrane | -1.52 | 0.57 | 948 |
| *CD3D* | CD3d molecule, delta (CD3-TCR complex) | Plasma Membrane | 2.59 | 1.20 | 915 |
| *CD3E* | CD3e molecule, epsilon (CD3-TCR complex) | Plasma Membrane | 2.17 | 0.55 | 916 |
| *CD3G* | CD3g molecule, gamma (CD3-TCR complex) | Plasma Membrane | 2.85 | 1.22 | 917 |
| *CD74* | CD74 molecule, major histocompatibility complex, class II invariant chain | Plasma Membrane | 0.57 | -0.38 | 972 |
| *CD79B* | CD79b molecule, immunoglobulin-associated beta | Plasma Membrane | 2.51 | 1.28 | 974 |
| *CD86* | CD86 molecule | Plasma Membrane | 2.07 | -0.12 | 942 |
| *CR1* | complement component (3b/4b) receptor 1 (Knops blood group) | Plasma Membrane | 1.13 | 0.05 | 1378 |
| *CXADR* | coxsackie virus and adenovirus receptor | Plasma Membrane | -1.66 | -1.32 | 1525 |
| *EDNRA* | endothelin receptor type A | Plasma Membrane | -0.07 | 1.00 | 1909 |
| *F3* | coagulation factor III (thromboplastin, tissue factor) | Plasma Membrane | -1.93 | -0.19 | 2152 |
| *FAS* | Fas (TNF receptor superfamily, member 6) | Plasma Membrane | 1.58 | 0.05 | 355 |
| *FCGR2A* | Fc fragment of IgG, low affinity IIa, receptor (CD32) | Plasma Membrane | 3.57 | -0.27 | 2212 |
| *FCGR2B* | Fc fragment of IgG, low affinity IIb, receptor (CD32) | Plasma Membrane | 3.57 | -0.27 | 2213 |
| *FCGR3A* | Fc fragment of IgG, low affinity IIIa, receptor (CD16a) | Plasma Membrane | 1.60 | -0.57 | 2214 |
| *FCGR3B* | Fc fragment of IgG, low affinity IIIb, receptor (CD16b) | Plasma Membrane | 1.60 | -0.57 | 2215 |
| *HLA-DMA* | major histocompatibility complex, class II, DM alpha | Plasma Membrane | 2.05 | 0.24 | 3108 |
| *HLA-DMB* | major histocompatibility complex, class II, DM beta | Plasma Membrane | 1.80 | 0.00 | 3109 |
| *HLA-DQA1* | major histocompatibility complex, class II, DQ alpha 1 | Plasma Membrane | 0.86 | -0.40 | 3117 |
| *HLA-DRA* | major histocompatibility complex, class II, DR alpha | Plasma Membrane | 2.01 | -0.31 | 3122 |
| *HLA-F* | major histocompatibility complex, class I, F | Plasma Membrane | 2.56 | 0.93 | 3134 |
| *IFNAR1* | interferon (alpha, beta and omega) receptor 1 | Plasma Membrane | 0.92 | 4.19 | 3454 |
| *IL11RA* | interleukin 11 receptor, alpha | Plasma Membrane | -0.86 | 1.71 | 3590 |
| *IL1RAP* | interleukin 1 receptor accessory protein | Plasma Membrane | 1.91 | 2.41 | 3556 |
| *IL23R* | interleukin 23 receptor | Plasma Membrane | 2.39 | 0.82 | 149233 |
| *IL2RA* | interleukin 2 receptor, alpha | Plasma Membrane | 1.83 | 0.30 | 3559 |
| *IL4R* | interleukin 4 receptor | Plasma Membrane | 0.87 | 2.76 | 3566 |
| *IL6R* | interleukin 6 receptor | Plasma Membrane | 0.50 | 1.85 | 3570 |
| *ITGB1* | integrin, beta 1 (fibronectin receptor, beta polypeptide, antigen CD29 includes MDF2, MSK12) | Plasma Membrane | -0.56 | 0.49 | 3688 |
| *ITGB3* | integrin, beta 3 (platelet glycoprotein IIIa, antigen CD61) | Plasma Membrane | 0.59 | 1.59 | 3690 |
| *ITGB7* | integrin, beta 7 | Plasma Membrane | 1.96 | 0.88 | 3695 |
| *LTBR* | lymphotoxin beta receptor (TNFR superfamily, member 3) | Plasma Membrane | 0.46 | 3.18 | 4055 |
| *NCR1* | natural cytotoxicity triggering receptor 1 | Plasma Membrane | 2.66 | -0.43 | 9437 |
| *OLR1* | oxidized low density lipoprotein (lectin-like) receptor 1 | Plasma Membrane | 0.96 | -0.62 | 4973 |
| *TLR10* | toll-like receptor 10 | Plasma Membrane | 1.83 | 0.63 | 81793 |
| *TLR2* | toll-like receptor 2 | Plasma Membrane | 3.52 | 0.65 | 7097 |
| *TLR4* | toll-like receptor 4 | Plasma Membrane | 1.38 | 0.07 | 7099 |
|  |  |  |  |  |  |
| **Transporters** |  |  |  |  |  |
| *A2M* | alpha-2-macroglobulin | Extracellular Space | 1.17 | 0.58 | 2 |
| *APOB* | apolipoprotein B (including Ag(x) antigen) | Extracellular Space | -1.14 | 0.39 | 338 |
| *SFTPA1* | surfactant protein A1 | Extracellular Space | 1.69 | 2.37 | 653509 |
| *TAP1* | transporter 1, ATP-binding cassette, sub-family B (MDR/TAP) | Cytoplasm | 3.18 | 0.37 | 6890 |
| *TF* | transferrin | Extracellular Space | 2.10 | 0.01 | 7018 |
|  |  |  |  |  |  |
| **Others** |  |  |  |  |  |
| *AMICA1* | adhesion molecule, interacts with CXADR antigen 1 | Plasma Membrane | 2.84 | 0.92 | 120425 |
| *ANXA1* | annexin A1 | Plasma Membrane | -1.80 | -0.94 | 301 |
| *APP* | amyloid beta (A4) precursor protein | Plasma Membrane | -1.51 | -0.02 | 351 |
| *BGN* | biglycan | Extracellular Space | 0.31 | 1.37 | 633 |
| *BID* | BH3 interacting domain death agonist | Cytoplasm | 2.16 | 0.64 | 637 |
| *BLNK* | B-cell linker | Cytoplasm | 1.15 | 0.09 | 29760 |
| *C4B (includes others)* | complement component 4B (Chido blood group) | Extracellular Space | 1.94 | 0.51 | 720|100293534|721 |
| *CADM1* | cell adhesion molecule 1 | Plasma Membrane | 0.30 | 1.48 | 23705 |
| *CAV1* | caveolin 1, caveolae protein, 22kDa | Plasma Membrane | -2.21 | -0.02 | 857 |
| *CD1A* | CD1a molecule | Plasma Membrane | 1.52 | 2.15 | 909 |
| *CD1D* | CD1d molecule | Plasma Membrane | -0.89 | 0.81 | 912 |
| *CD200R1* | CD200 receptor 1 | Plasma Membrane | 1.99 | 0.51 | 131450 |
| *CD226* | CD226 molecule | Plasma Membrane | 2.50 | 0.04 | 10666 |
| *CD48* | CD48 molecule | Plasma Membrane | 1.81 | 1.03 | 962 |
| *CD55* | CD55 molecule, decay accelerating factor for complement (Cromer blood group) | Plasma Membrane | -0.01 | 2.29 | 1604 |
| *CD81* | CD81 molecule | Plasma Membrane | 0.04 | 1.22 | 975 |
| *CD9* | CD9 molecule | Plasma Membrane | -2.00 | -1.08 | 928 |
| *CLU* | clusterin | Extracellular Space | 2.26 | -0.48 | 1191 |
| *COL1A1* | collagen, type I, alpha 1 | Extracellular Space | 2.28 | 1.85 | 1277 |
| *COL4A1* | collagen, type IV, alpha 1 | Extracellular Space | 0.73 | 2.78 | 1282 |
| *COL4A3* | collagen, type IV, alpha 3 (Goodpasture antigen) | Extracellular Space | -1.29 | -0.04 | 1285 |
| *COL4A4* | collagen, type IV, alpha 4 | Extracellular Space | -1.30 | -0.19 | 1286 |
| *COL4A5* | collagen, type IV, alpha 5 | Extracellular Space | -1.12 | 0.17 | 1287 |
| *COL4A6* | collagen, type IV, alpha 6 | Extracellular Space | -1.45 | -0.45 | 1288 |
| *CSPG4* | chondroitin sulfate proteoglycan 4 | Plasma Membrane | 0.73 | 1.91 | 1464 |
| *DEFA1 (includes others)* | defensin, alpha 1 | Cytoplasm | -3.24 | -0.52 | 728358|1667|1668 |
| *DOCK8* | dedicator of cytokinesis 8 | Cytoplasm | 0.31 | -0.48 | 81704 |
| *ELMO1* | engulfment and cell motility 1 | Cytoplasm | 0.93 | 2.74 | 9844 |
| *F11R* | F11 receptor | Plasma Membrane | 0.52 | 2.13 | 50848 |
| *FBN1* | fibrillin 1 | Extracellular Space | -0.26 | 1.46 | 2200 |
| *FGG* | fibrinogen gamma chain | Extracellular Space | -2.25 | -1.07 | 2266 |
| *GRB2* | growth factor receptor-bound protein 2 | Cytoplasm | 0.85 | 0.07 | 2885 |
| *HLA-A* | major histocompatibility complex, class I, A | Plasma Membrane | 2.36 | 1.44 | 3105 |
| *HLA-C* | major histocompatibility complex, class I, C | Plasma Membrane | 2.56 | 0.93 | 3107 |
| *HLA-DQB1* | major histocompatibility complex, class II, DQ beta 1 | Plasma Membrane | 1.58 | 0.14 | 3119 |
| *HSP90B1* | heat shock protein 90kDa beta (Grp94), member 1 | Cytoplasm | 0.88 | 3.07 | 7184 |
| *HSPA1A/HSPA1B* | heat shock 70kDa protein 1A | Cytoplasm | 0.20 | -1.52 | 3303|3304 |
| *ICAM2* | intercellular adhesion molecule 2 | Plasma Membrane | 0.20 | 1.13 | 3384 |
| *ICOS* | inducible T-cell co-stimulator | Plasma Membrane | 3.30 | 1.21 | 29851 |
| *ITGA1* | integrin, alpha 1 | Plasma Membrane | -0.08 | 1.14 | 3672 |
| *ITGA5* | integrin, alpha 5 (fibronectin receptor, alpha polypeptide) | Plasma Membrane | 1.79 | 1.55 | 3678 |
| *ITGAL* | integrin, alpha L (antigen CD11A (p180), lymphocyte function-associated antigen 1; alpha polypeptide) | Plasma Membrane | 1.98 | -0.52 | 3683 |
| *ITGAX* | integrin, alpha X (complement component 3 receptor 4 subunit) | Plasma Membrane | 1.00 | -0.76 | 3687 |
| *ITGB2* | integrin, beta 2 (complement component 3 receptor 3 and 4 subunit) | Plasma Membrane | 2.01 | 0.21 | 3689 |
| *LAMA2* | laminin, alpha 2 | Extracellular Space | -0.08 | 1.69 | 3908 |
| *LCP2* | lymphocyte cytosolic protein 2 (SH2 domain containing leukocyte protein of 76kDa) | Cytoplasm | 2.56 | -0.01 | 3937 |
| *LGALS3* | lectin, galactoside-binding, soluble, 3 | Extracellular Space | 1.21 | -0.58 | 3958 |
| *LY86* | lymphocyte antigen 86 | Plasma Membrane | 1.98 | -0.15 | 9450 |
| *LY96* | lymphocyte antigen 96 | Plasma Membrane | 1.44 | -0.51 | 23643 |
| *MYH10* | myosin, heavy chain 10, non-muscle | Cytoplasm | -0.32 | 1.62 | 4628 |
| *NCAM1* | neural cell adhesion molecule 1 | Plasma Membrane | -1.40 | 0.04 | 4684 |
| *NLRP3* | NLR family, pyrin domain containing 3 | Cytoplasm | 1.24 | -0.27 | 114548 |
| *NOD1* | nucleotide-binding oligomerization domain containing 1 | Cytoplasm | 1.98 | 1.06 | 10392 |
| *NTS* | neurotensin | Extracellular Space | -1.72 | -0.16 | 4922 |
| *PLEK* | pleckstrin | Cytoplasm | 2.43 | -0.65 | 5341 |
| *PRF1* | perforin 1 (pore forming protein) | Cytoplasm | 3.89 | 0.25 | 5551 |
| *PROS1* | protein S (alpha) | Extracellular Space | -2.50 | -0.42 | 5627 |
| *PSAP* | prosaposin | Extracellular Space | 1.33 | 0.27 | 5660 |
| *PSME1* | proteasome (prosome, macropain) activator subunit 1 (PA28 alpha) | Cytoplasm | 1.43 | -0.04 | 5720 |
| *PXN* | paxillin | Cytoplasm | 0.54 | 1.96 | 5829 |
| *PYY* | peptide YY | Extracellular Space | 1.41 | -0.69 | 5697 |
| *RASGRP1* | RAS guanyl releasing protein 1 (calcium and DAG-regulated) | Cytoplasm | 2.08 | 1.82 | 10125 |
| *RGS1* | regulator of G-protein signaling 1 | Plasma Membrane | 2.58 | -0.75 | 5996 |
| *S100A8* | S100 calcium binding protein A8 | Cytoplasm | 1.77 | 1.21 | 6279 |
| *S100A9* | S100 calcium binding protein A9 | Cytoplasm | 0.93 | 1.90 | 6280 |
| *SAA1* | serum amyloid A1 | Extracellular Space | 5.13 | -1.03 | 6288 |
| *SELL* | selectin L | Plasma Membrane | 0.55 | -0.37 | 6402 |
| *SERPINE1* | serpin peptidase inhibitor, clade E (nexin, plasminogen activator inhibitor type 1), member 1 | Extracellular Space | 1.57 | 2.89 | 5054 |
| *SERPINF2* | serpin peptidase inhibitor, clade F (alpha-2 antiplasmin, pigment epithelium derived factor), member 2 | Extracellular Space | 1.15 | -0.06 | 5345 |
| *SH2B3* | SH2B adaptor protein 3 | Cytoplasm | 1.41 | 0.89 | 10019 |
| *SH2D1A* | SH2 domain containing 1A | Cytoplasm | 2.63 | 0.56 | 4068 |
| *SH3BP2* | SH3-domain binding protein 2 | Cytoplasm | 2.16 | 0.53 | 6452 |
| *SOCS2* | suppressor of cytokine signaling 2 | Cytoplasm | -0.63 | 0.26 | 8835 |
| *SPARC* | secreted protein, acidic, cysteine-rich (osteonectin) | Extracellular Space | -0.52 | 0.60 | 6678 |
| *SRGN* | serglycin | Extracellular Space | 1.22 | 1.04 | 5552 |
| *TAC1* | tachykinin, precursor 1 | Extracellular Space | -1.97 | -1.52 | 6863 |
| *TFPI* | tissue factor pathway inhibitor (lipoprotein-associated coagulation inhibitor) | Extracellular Space | -1.16 | 0.04 | 7035 |
| *THBS1* | thrombospondin 1 | Extracellular Space | 0.53 | -1.70 | 7057 |
| *THBS2* | thrombospondin 2 | Extracellular Space | 0.39 | -0.97 | 7058 |
| *TIMP1* | TIMP metallopeptidase inhibitor 1 | Extracellular Space | 2.15 | 1.94 | 7076 |
| *TREM1* | triggering receptor expressed on myeloid cells 1 | Plasma Membrane | 0.41 | 1.31 | 54210 |
| *TXNIP* | thioredoxin interacting protein | Cytoplasm | -1.08 | 0.64 | 10628 |
| *VCAN* | versican | Extracellular Space | 2.42 | 0.59 | 1462 |
| *VEGFB* | vascular endothelial growth factor B | Extracellular Space | -1.74 | -1.24 | 7423 |
| *VTN* | vitronectin | Extracellular Space | 1.23 | 1.57 | 7448 |
| *VWF* | von Willebrand factor | Extracellular Space | 0.43 | 1.11 | 7450 |
| *WASL* | Wiskott-Aldrich syndrome-like | Cytoplasm | -0.90 | 0.17 | 8976 |
