# Supplementary material for: Early innate immunity determines outcome of Mycobacterium tuberculosis pulmonary infection in rabbits
Source: Cell Commun Signal. 2013 Aug 19;11:60. doi: 10.1186/1478-811X-11-60 (PMC3765177; doi:10.1186/1478-811X-11-60)
Supplement: Additional file 4: Table S5 — qRT-PCR analysis of gene expression in the blood of uninfected and HN878-infected rabbits at 3 hours. [file 1478-811X-11-60-S4.doc]

**Supplementary Table S5.** qRT-PCR analysis of gene expression in the blood of uninfected and HN878-infected rabbits at three hours

| **Gene** | **Uninfected** | | **HN878** | |
| --- | --- | --- | --- | --- |
| **Symbol** | ***Avg*** | ***sd*** | ***Avg*** | ***sd*** |
| *TNF* | 0.117 | 0.053 | 0.091 | 0.033 |
| *IL4R* | 0.009 | 0.009 | 0.018 | 0.015 |
| *CD36* | 0.120 | 0.074 | 0.144 | 0.041 |
| *CXCL10* | 0.023 | 0.022 | 0.055 | 0.047 |
| *IL1A* | 0.101 | 0.100 | 0.101 | 0.121 |
| *CAV1* | 0.217 | 0.111 | 0.171 | 0.076 |
| *TGFB2* | 0.015 | 0.012 | 0.021 | 0.014 |
| *SPP1* | 0.119 | 0.062 | 0.153 | 0.042 |
| *CCL4* | 0.035 | 0.030 | 0.051 | 0.037 |
| *IL18* | 0.136 | 0.069 | 0.087 | 0.025 |
| *CCL2* | 0.071 | 0.078 | 0.091 | 0.095 |
| *IRF5* | 0.028 | 0.012 | 0.025 | 0.015 |
| Avg- average | |  |  |  |
| sd-standard deviation | | |  |  |
