# Supplementary material for: Early innate immunity determines outcome of Mycobacterium tuberculosis pulmonary infection in rabbits
Source: Cell Commun Signal. 2013 Aug 19;11:60. doi: 10.1186/1478-811X-11-60 (PMC3765177; doi:10.1186/1478-811X-11-60)
Supplement: Additional file 5: Table S6 — List of top transcription regulator genes differentially expressed in the lungs of Mtb-infected rabbits at 3 hours. [file 1478-811X-11-60-S5.doc]

| **Supplementary Table S6.** List of top transcription regulator genes differentially expressed in the lungs of Mtb infected rabbits at three hours   |  | **Log_2_ Ratio** | | **Z score *** | | **p-value** | |  | | --- | --- | --- | --- | --- | --- | --- | --- | | **Regulator** | **HN878** | **CDC1551** | **HN878** | **CDC1551** | **HN878** | **CDC1551** | **Target molecules in dataset** | | *STAT1* | 4.096 | 0.264 | 4.671 | 0.144 | 2.10E-31 | 2.10E-31 | *A2M,B2M,CASP1,CASP4,CASP8,CCL19,CCL2,CCL3,CCL3L1/CCL3L3,CCL4,*  *CCL5,CD14,CD274,CD86,CDKN1A,CFB,CIITA,CSF2,CXCL10,CXCL3,CXCL9,*  *FAS,GATA3,GBP1,GBP5,GZMB,IGF1R,IL15,IL8,IRF1,IRF5,IRF7,IRF8,ITGAX,*  *JAK2,JUN,LY96,PF4,PRF1,PRL,PSMB10,PSMB8,PSMB9,PSME1,PSME2,PTGS2,*  *STAT1,TAP1,TLR4,TNF* | | *IRF5* | 2.509 | 0.297 | 2.734 | -0.369 | 3.50E-06 | 3.50E-06 | *CCL19,CCL21,CCL3,CCL4,CCL5,CDKN1A,IRF5,RIPK1,TNF* | | *IRF8* | 2.471 | 0.889 | 2.636 | -0.815 | 2.73E-13 | 2.73E-13 | *B2M,CCL5,CD86,CDKN1A,CFB,CIITA,CSF1R,CTSS,CXCL16,CXCL9,CYBB,FAS,*  *GBP1,IL15,IL18,IL5,IRF8,JAK1,STAT1,TNF* | | *IRF7* | 2.25 | 0.528 | 4.997 | -0.137 | 3.21E-22 | 3.21E-22 | *CASP4,CCL19,CCL5,CXCL10,DDX58,DHX58,GBP1,GBP4,GBP5,IFIT3,IL15,IRF1,*  *IRF8,ITGAX,JAK2,MX1,MX2,OAS2,OAS3,PSMB10,PSMB8,PSMB9,PSME1,PSME2,*  *S100A8,STAT1,TAP1,TAP2,TLR4,TNFSF13B,XAF1* | | *IRF1* | 1.845 | -0.596 | 3.954 | -0.462 | 3.67E-29 | 3.67E-29 | *B2M,C1R,CASP1,CASP8,CCL19,CCL2,CCL5,CDKN1A,CFB,CIITA,CTSS,CXCL10,*  *CXCL16,CYBB,FPR2,IFIT3,IL15,IL18,IL5,IL8,IRF1,IRF5,IRF7,JAK2,LTB,MX1,OAS2,*  *PF4,PSMB10,PSMB8,PSMB9,PSME1,PSME2,PTGS2,SELL,STAT1,TAP1,TAP2,TNF* | | *CIITA* | 1.704 | 1.704 | 2.134 | -1.145 | 3.54E-10 | 3.54E-10 | *B2M,CD74,COL1A1,COL1A2,HLA-A,HLA-DMB,HLA-DPA1,HLA-DQA1,HLA-DQB1,*  *HLA-DRA,IL5* | | *JUN* | 1.066 | 2.917 | 2.03 | -0.084 | 3.13E-21 | 3.13E-21 | *A2M,ANXA1,APP,BIRC3,CAPN2,CAV1,CCL2,CD14,CD274,CD44,CDC20,CDK1,*  *CDKN1A,CLU,COL1A1,COL1A2,CSF1R,CSF2,CXCL10,CXCL16,EDN1,F3,FAS,*  *FLNC,IGF1R,IL2RA,IL8,ITGB1,JUN,LGALS3,MMP1,NCAM1,NFKBIA,NTS,PAK3,*  *PLAU,PRL,PTEN,PTGS2,RHOB,SAA1,SERPINE1,SPARC,SPP1,STAT1,THBS1,TIMP1,*  *TNF* | | *NFKBIA* | 0.5 | -0.43 | 2.606 | 1.321 | 6.27E-32 | 6.27E-32 | *A2M,BID,BIRC3,BMP2,CASP4,CASP8,CCL2,CCL20,CCL3L1/CCL3L3,CCL5,CD86,*  *CDKN1A,CLU,COL1A2,COL3A1,COL5A2,CSF2,CSF3,CTSB,CXCL1,CXCL10,CXCL2,*  *CXCL3,CXCL6,DAG1,F3,FAS,FN1,GRK5,GZMB,HLA-C,HMGB1,IL15,IL1A,IL1RN,*  *IL2RA,IL5,IL8,IRF1,ITGB1,JUN,MMP1,NFKB2,NFKBIA,*  *NFKBIE,NID1,PLAU,PRL,PTEN,PTGS2,RIPK1,S100A8,S100A9,SFTPA1,SOD1,TAC1,*  *TFRC,TGFB2,TIMP1,TLR2,TLR4,TNF,TOLLIP,VEGFC* | | *HMGB1* | -0.411 | -0.411 | 2.197 | -2.279 | 2.96E-11 | 2.96E-11 | *CCL2,CCL20,CCL3,CCL4,CD86,CDKN1A,CXCL3,CXCL5,IL1A,IL8,MMP1,PTGS2,*  *TLR2,TLR4,TNF* | |  |  |  |  |  |
| --- | --- | --- | --- | --- | --- | --- | --- | --- | --- | --- | --- | --- | --- | --- | --- | --- | --- | --- | --- | --- | --- | --- | --- | --- | --- | --- | --- | --- | --- | --- | --- | --- | --- | --- | --- | --- | --- | --- | --- | --- | --- | --- | --- | --- | --- | --- | --- | --- | --- | --- | --- | --- | --- | --- | --- | --- | --- | --- | --- | --- | --- | --- | --- | --- | --- | --- | --- | --- | --- | --- | --- | --- | --- | --- | --- | --- | --- | --- | --- | --- | --- | --- | --- | --- | --- | --- | --- | --- | --- | --- | --- | --- | --- |

*z-score ≥ +2 indicates activation and ≤ -2 denotes inhibition of the downstream network.
