# Supplementary material for: Early innate immunity determines outcome of Mycobacterium tuberculosis pulmonary infection in rabbits
Source: Cell Commun Signal. 2013 Aug 19;11:60. doi: 10.1186/1478-811X-11-60 (PMC3765177; doi:10.1186/1478-811X-11-60)
Supplement: Additional file 6: Table S7 — List of SDEG involved in the canonical STAT1 mechanistic network in the lungs of Mtb-infected rabbits at 3 hours. [file 1478-811X-11-60-S6.doc]

**Supplementary Table S7.** List of SDEG involved in the canonical STAT1 mechanistic network in the lungs of Mtb-infected rabbits at three hours

|  |  |  | **Log_2_ Ratio** | |  |
| --- | --- | --- | --- | --- | --- |
| **Gene Symbol** | **Gene Name** | **Location** | **HN878** | **CDC1551** | **Human Gene ID** |
| **Cytokine/chemokines** |  |  |  |  |  |
| *CCL18* | chemokine (C-C motif) ligand 18 | Extracellular Space | 2.008 | 0.126 | 6362 |
| *CCL19* | chemokine (C-C motif) ligand 19 | Extracellular Space | 3.217 | 1.056 | 6363 |
| *CCL2* | chemokine (C-C motif) ligand 2 | Extracellular Space | 1.866 | -1.461 | 6347 |
| *CCL20* | chemokine (C-C motif) ligand 20 | Extracellular Space | -0.700 | -2.054 | 6364 |
| *CCL3* | chemokine (C-C motif) ligand 3 | Extracellular Space | 2.008 | 0.126 | 6348 |
| *CCL3L1/CCL3L3* | chemokine (C-C motif) ligand 3-like 1 | Extracellular Space | 2.008 | 0.126 | 6349|414062 |
| *CCL4* | chemokine (C-C motif) ligand 4 | Extracellular Space | 4.232 | -0.540 | 6351 |
| *CCL5* | chemokine (C-C motif) ligand 5 | Extracellular Space | 3.313 | -0.346 | 6352 |
| *CSF2* | colony stimulating factor 2 | Extracellular Space | -1.918 | -1.438 | 1437 |
| *CSF3* | colony stimulating factor 3 | Extracellular Space | 0.185 | 2.528 | 1440 |
| *CTF1* | cardiotrophin 1 | Extracellular Space | 0.194 | 1.990 | 1489 |
| *CXCL1* | chemokine (C-X-C motif) ligand 1 | Extracellular Space | -0.245 | -2.130 | 2919 |
| *CXCL10* | chemokine (C-X-C motif) ligand 10 | Extracellular Space | 3.976 | -0.733 | 3627 |
| *CXCL13* | chemokine (C-X-C motif) ligand 13 | Extracellular Space | 2.605 | 1.521 | 10563 |
| *CXCL16* | chemokine (C-X-C motif) ligand 16 | Extracellular Space | 0.882 | 3.070 | 58191 |
| *CXCL2* | chemokine (C-X-C motif) ligand 2 | Extracellular Space | -0.245 | -2.130 | 2920 |
| *CXCL3* | chemokine (C-X-C motif) ligand 3 | Extracellular Space | -0.245 | -2.130 | 2921 |
| *CXCL5* | chemokine (C-X-C motif) ligand 5 | Extracellular Space | 1.568 | -1.512 | 6374 |
| *CXCL6* | chemokine (C-X-C motif) ligand 6 | Extracellular Space | 1.568 | -1.512 | 6372 |
| *CXCL9* | chemokine (C-X-C motif) ligand 9 | Extracellular Space | 5.122 | 0.080 | 4283 |
| *EDN1* | endothelin 1 | Extracellular Space | 0.205 | 1.342 | 1906 |
| *IL15* | interleukin 15 | Extracellular Space | 1.301 | -0.350 | 3600 |
| *IL18* | interleukin 18 | Extracellular Space | -1.199 | -0.638 | 3606 |
| *IL1A* | interleukin 1, alpha | Extracellular Space | 0.657 | -1.996 | 3552 |
| *IL1RN* | interleukin 1 receptor antagonist | Extracellular Space | 2.262 | -0.316 | 3557 |
| *IL22* | interleukin 22 | Extracellular Space | 1.233 | 0.022 | 50616 |
| *IL5* | interleukin 5 | Extracellular Space | -0.311 | 1.793 | 3567 |
| *IL8* | interleukin 8 | Extracellular Space | 1.595 | -0.988 | 3576 |
| *LTB* | lymphotoxin beta (TNF superfamily, member 3) | Extracellular Space | 2.308 | 0.682 | 4050 |
| *PF4* | platelet factor 4 | Extracellular Space | -0.355 | 1.176 | 5196 |
| *PRL* | prolactin | Extracellular Space | 1.268 | 1.929 | 5617 |
| *SPP1* | secreted phosphoprotein 1 | Extracellular Space | 1.500 | -3.276 | 6696 |
| *THPO* | thrombopoietin | Extracellular Space | 2.884 | 4.571 | 7066 |
| *TNF* | tumor necrosis factor | Extracellular Space | 3.313 | -0.524 | 7124 |
| *TNFSF13B* | tumor necrosis factor superfamily, member 13b | Extracellular Space | 1.989 | -0.747 | 10673 |
| **Enzymes** |  |  |  |  |  |
| *ADCY7* | adenylate cyclase 7 | Plasma Membrane | 0.267 | -0.826 | 113 |
| *ARHGAP5* | Rho GTPase activating protein 5 | Cytoplasm | -0.980 | 0.020 | 394 |
| *BIRC3* | baculoviral IAP repeat containing 3 | Cytoplasm | 2.535 | -0.543 | 330 |
| *CD274* | CD274 molecule | Plasma Membrane | 2.220 | -0.943 | 29126 |
| *CD38* | CD38 molecule | Plasma Membrane | 3.954 | 0.572 | 952 |
| *CD44* | CD44 molecule (Indian blood group) | Plasma Membrane | 0.821 | -0.571 | 960 |
| *CYBB* | cytochrome b-245, beta polypeptide | Cytoplasm | 2.729 | -0.825 | 1536 |
| *DDX58* | DEAD (Asp-Glu-Ala-Asp) box polypeptide 58 | Cytoplasm | 1.251 | -0.172 | 23586 |
| *FN1* | fibronectin 1 | Extracellular Space | 0.330 | 1.539 | 2335 |
| *GBP1* | guanylate binding protein 1 | Cytoplasm | 5.723 | -0.074 | 2633 |
| *GBP4* | guanylate binding protein 4 | Cytoplasm | 2.336 | 1.256 | 115361 |
| *GBP5* | guanylate binding protein 5 | Plasma Membrane | 4.634 | 0.556 | 115362 |
| *GBP7* | guanylate binding protein 7 | Cytoplasm | 2.336 | 1.256 | 388646 |
| *GNAI2* | guanine nucleotide binding protein (G protein), alpha inhibiting activity polypeptide 2 | Plasma Membrane | -1.568 | -0.461 | 2771 |
| *HSP90AA1* | heat shock protein 90kDa alpha, class A member 1 | Cytoplasm | 1.072 | -0.376 | 3320 |
| *LAMB2* | laminin, beta 2 (laminin S) | Extracellular Space | 0.055 | 1.438 | 3913 |
| *MX1* | myxovirus (influenza virus) resistance 1, interferon-inducible protein p78 (mouse) | Nucleus | 1.129 | -0.184 | 4599 |
| *MX2* | myxovirus (influenza virus) resistance 2 (mouse) | Nucleus | 1.129 | -0.184 | 4600 |
| *MYH7* | myosin, heavy chain 7, cardiac muscle, beta | Cytoplasm | 0.470 | 2.588 | 4625 |
| *NOS3* | nitric oxide synthase 3 (endothelial cell) | Cytoplasm | -0.007 | 1.207 | 4846 |
| *OAS2* | 2'-5'-oligoadenylate synthetase 2, 69/71kDa | Cytoplasm | 3.124 | -0.086 | 4939 |
| *OAS3* | 2'-5'-oligoadenylate synthetase 3, 100kDa | Cytoplasm | -1.325 | -0.324 | 4940 |
| *OCLN* | occludin | Plasma Membrane | -1.194 | -0.113 | 100506658 |
| *PTGS2* | prostaglandin-endoperoxide synthase 2 (prostaglandin G/H synthase and cyclooxygenase) | Cytoplasm | 0.601 | -0.348 | 5743 |
| *RAC2* | ras-related C3 botulinum toxin substrate 2 (rho family, small GTP binding protein Rac2) | Cytoplasm | 1.787 | 0.305 | 5880 |
| *RHOB* | ras homolog family member B | Cytoplasm | 0.158 | 1.044 | 388 |
| *SMURF2* | SMAD specific E3 ubiquitin protein ligase 2 | Cytoplasm | -0.613 | 0.252 | 64750 |
| *SOD1* | superoxide dismutase 1, soluble | Cytoplasm | -1.633 | -0.736 | 6647 |
| *UBE2B* | ubiquitin-conjugating enzyme E2B | Cytoplasm | -0.882 | 0.064 | 7320 |
| *UBE2C* | ubiquitin-conjugating enzyme E2C | Cytoplasm | 2.040 | 0.452 | 11065 |
| *UBE2E1* | ubiquitin-conjugating enzyme E2E 1 | Cytoplasm | -1.142 | 0.302 | 7324 |
| *WWP1* | WW domain containing E3 ubiquitin protein ligase 1 | Cytoplasm | -0.931 | 0.045 | 11059 |
| **G-protein coupled receptors** | |  |  |  |  |
| *BDKRB1* | bradykinin receptor B1 | Plasma Membrane | 3.227 | 0.185 | 623 |
| *CXCR2* | chemokine (C-X-C motif) receptor 2 | Plasma Membrane | 1.054 | 0.067 | 3579 |
| *EDNRB* | endothelin receptor type B | Plasma Membrane | -1.009 | 1.630 | 1910 |
| *FPR1* | formyl peptide receptor 1 | Plasma Membrane | 3.466 | -0.370 | 2357 |
| *FPR2* | formyl peptide receptor 2 | Plasma Membrane | 3.011 | -0.266 | 2358 |
| *FZD2* | frizzled family receptor 2 | Plasma Membrane | -0.110 | 1.059 | 2535 |
| *GPR17* | G protein-coupled receptor 17 | Plasma Membrane | 0.211 | 1.042 | 2840 |
| *HTR2A* | 5-hydroxytryptamine (serotonin) receptor 2A, G protein-coupled | Plasma Membrane | 0.458 | 3.177 | 3356 |
| *P2RY1* | purinergic receptor P2Y, G-protein coupled, 1 | Plasma Membrane | -1.191 | -0.134 | 5028 |
| *TACR2* | tachykinin receptor 2 | Plasma Membrane | -0.181 | 0.973 | 6865 |
| *VIPR1* | vasoactive intestinal peptide receptor 1 | Plasma Membrane | -0.122 | 1.683 | 7433 |
| **Growth factors** | |  |  |  |  |
| *ANGPT1* | angiopoietin 1 | Extracellular Space | -0.664 | 1.127 | 284 |
| *BMP2* | bone morphogenetic protein 2 | Extracellular Space | -0.658 | 0.602 | 650 |
| *HGF* | hepatocyte growth factor (hepapoietin A; scatter factor) | Extracellular Space | -0.387 | 0.814 | 3082 |
| *INHBA* | inhibin, beta A | Extracellular Space | 1.544 | 3.026 | 3624 |
| *PDGFB* | platelet-derived growth factor beta polypeptide | Extracellular Space | 0.691 | 1.843 | 5155 |
| *TGFB2* | transforming growth factor, beta 2 | Extracellular Space | -1.382 | -0.238 | 7042 |
| *VEGFC* | vascular endothelial growth factor C | Extracellular Space | -0.410 | 1.173 | 7424 |
| *TRPC3* | transient receptor potential cation channel, subfamily C, member 3 | Plasma Membrane | -0.276 | 1.006 | 7222 |
| **Kinase** |  |  |  |  |  |
| *CAMK2D* | calcium/calmodulin-dependent protein kinase II delta | Cytoplasm | -1.125 | -0.461 | 817 |
| *CDK1* | cyclin-dependent kinase 1 | Nucleus | 1.148 | -0.057 | 983 |
| *CDKN1A* | cyclin-dependent kinase inhibitor 1A (p21, Cip1) | Nucleus | -0.027 | -1.194 | 1026 |
| *CSF1R* | colony stimulating factor 1 receptor | Plasma Membrane | 1.853 | -0.206 | 1436 |
| *FYN* | FYN oncogene related to SRC, FGR, YES | Plasma Membrane | 2.278 | 1.569 | 2534 |
| *GRK5* | G protein-coupled receptor kinase 5 | Plasma Membrane | -0.531 | 0.788 | 2869 |
| *GSK3B* | glycogen synthase kinase 3 beta | Nucleus | -1.378 | -0.174 | 2932 |
| *HCK* | hemopoietic cell kinase | Cytoplasm | 2.487 | 0.177 | 3055 |
| *IKBKE* | inhibitor of kappa light polypeptide gene enhancer in B-cells, kinase epsilon | Cytoplasm | 2.034 | -0.519 | 9641 |
| *JAK2* | Janus kinase 2 | Cytoplasm | 1.715 | -0.099 | 3717 |
| *LYN* | v-yes-1 Yamaguchi sarcoma viral related oncogene homolog | Cytoplasm | 1.535 | 0.397 | 4067 |
| *MAP2K4* | mitogen-activated protein kinase kinase 4 | Cytoplasm | 0.287 | 2.244 | 6416 |
| *MAP2K6* | mitogen-activated protein kinase kinase 6 | Cytoplasm | 0.197 | 2.750 | 5608 |
| *MAP3K8* | mitogen-activated protein kinase kinase kinase 8 | Cytoplasm | 1.957 | 0.101 | 1326 |
| *MAPK1* | mitogen-activated protein kinase 1 | Cytoplasm | -1.562 | -0.253 | 5594 |
| *MYLK* | myosin light chain kinase | Cytoplasm | 0.148 | 1.355 | 4638 |
| *PAK3* | p21 protein (Cdc42/Rac)-activated kinase 3 | Cytoplasm | 0.874 | 2.799 | 5063 |
| *PDGFRA* | platelet-derived growth factor receptor, alpha polypeptide | Plasma Membrane | 0.991 | 2.423 | 5156 |
| *PRKCB* | protein kinase C, beta | Cytoplasm | 2.834 | 1.043 | 5579 |
| *PRKCD* | protein kinase C, delta | Cytoplasm | -0.235 | -1.129 | 5580 |
| *PRKG1* | protein kinase, cGMP-dependent, type I | Cytoplasm | -1.696 | -0.063 | 5592 |
| *RIPK1* | receptor (TNFRSF)-interacting serine-threonine kinase 1 | Plasma Membrane | 0.836 | -0.005 | 8737 |
| *SHC1* | SHC (Src homology 2 domain containing) transforming protein 1 | Cytoplasm | 0.055 | 1.233 | 6464 |
| *SPHK1* | sphingosine kinase 1 | Cytoplasm | 2.885 | -0.225 | 8877 |
| *SRC* | v-src sarcoma (Schmidt-Ruppin A-2) viral oncogene homolog (avian) | Cytoplasm | 2.736 | 0.655 | 6714 |
| *YES1* | v-yes-1 Yamaguchi sarcoma viral oncogene homolog 1 | Cytoplasm | -0.511 | 0.613 | 7525 |
| *ZAP70* | zeta-chain (TCR) associated protein kinase 70kDa | Plasma Membrane | 2.527 | 0.407 | 7535 |
| **Nuclear receptor** | |  |  |  |  |
| *NR4A1* | nuclear receptor subfamily 4, group A, member 1 | Nucleus | 1.953 | 2.710 | 3164 |
| **Others** |  |  |  |  |  |
| *ANXA1* | annexin A1 | Plasma Membrane | -1.798 | -0.938 | 301 |
| *APP* | amyloid beta (A4) precursor protein | Plasma Membrane | -1.514 | -0.017 | 351 |
| *BID* | BH3 interacting domain death agonist | Cytoplasm | 2.160 | 0.639 | 637 |
| *BLNK* | B-cell linker | Cytoplasm | 1.148 | 0.091 | 29760 |
| *C1QB* | complement component 1, q subcomponent, B chain | Extracellular Space | 3.790 | -0.190 | 713 |
| *C1QC* | complement component 1, q subcomponent, C chain | Extracellular Space | 3.402 | 0.642 | 714 |
| *C4B (includes others)* | complement component 4B (Chido blood group) | Extracellular Space | 1.943 | 0.506 | 720|100293534|721 |
| *CAV1* | caveolin 1, caveolae protein, 22kDa | Plasma Membrane | -2.212 | -0.021 | 857 |
| *CD1A* | CD1a molecule | Plasma Membrane | 1.521 | 2.149 | 909 |
| *CD1D* | CD1d molecule | Plasma Membrane | -0.890 | 0.806 | 912 |
| *CD226* | CD226 molecule | Plasma Membrane | 2.504 | 0.040 | 10666 |
| *CD55* | CD55 molecule, decay accelerating factor for complement (Cromer blood group) | Plasma Membrane | -0.010 | 2.294 | 1604 |
| *CD9* | CD9 molecule | Plasma Membrane | -1.996 | -1.081 | 928 |
| *CDC20* | cell division cycle 20 homolog (S. cerevisiae) | Nucleus | 1.828 | 0.407 | 991 |
| *CDH2* | cadherin 2, type 1, N-cadherin (neuronal) | Plasma Membrane | 0.322 | 2.328 | 1000 |
| *CLU* | clusterin | Extracellular Space | 2.257 | -0.482 | 1191 |
| *COL1A1* | collagen, type I, alpha 1 | Extracellular Space | 2.278 | 1.854 | 1277 |
| *COL1A2* | collagen, type I, alpha 2 | Extracellular Space | 1.191 | 2.372 | 1278 |
| *COL3A1* | collagen, type III, alpha 1 | Extracellular Space | 1.233 | 2.305 | 1281 |
| *COL4A1* | collagen, type IV, alpha 1 | Extracellular Space | 0.734 | 2.783 | 1282 |
| *COL5A2* | collagen, type V, alpha 2 | Extracellular Space | 0.127 | 1.283 | 1290 |
| *CYR61* | cysteine-rich, angiogenic inducer, 61 | Extracellular Space | -1.232 | -0.978 | 3491 |
| *F11R* | F11 receptor | Plasma Membrane | 0.520 | 2.127 | 50848 |
| *FGG* | fibrinogen gamma chain | Extracellular Space | -2.246 | -1.075 | 2266 |
| *FLNC* | filamin C, gamma | Cytoplasm | 0.025 | 0.900 | 2318 |
| *FYB* | FYN binding protein | Nucleus | 2.538 | 0.375 | 2533 |
| *GRB2* | growth factor receptor-bound protein 2 | Cytoplasm | 0.852 | 0.073 | 2885 |
| *HLA-A* | major histocompatibility complex, class I, A | Plasma Membrane | 2.364 | 1.435 | 3105 |
| *HLA-C* | major histocompatibility complex, class I, C | Plasma Membrane | 2.556 | 0.933 | 3107 |
| *HLA-DQB1* | major histocompatibility complex, class II, DQ beta 1 | Plasma Membrane | 1.585 | 0.142 | 3119 |
| *HSPA1A/HSPA1B* | heat shock 70kDa protein 1A | Cytoplasm | 0.197 | -1.516 | 3303|3304 |
| *ICOS* | inducible T-cell co-stimulator | Plasma Membrane | 3.299 | 1.209 | 29851 |
| *IFIT3* | interferon-induced protein with tetratricopeptide repeats 3 | Cytoplasm | 1.430 | -0.129 | 3437 |
| *ITGA5* | integrin, alpha 5 (fibronectin receptor, alpha polypeptide) | Plasma Membrane | 1.788 | 1.548 | 3678 |
| *ITGAE* | integrin, alpha E (antigen CD103, human mucosal lymphocyte antigen 1; alpha polypeptide) | Plasma Membrane | 1.559 | 0.049 | 3682 |
| *ITGAL* | integrin, alpha L (antigen CD11A (p180), lymphocyte function-associated antigen 1; alpha polypeptide) | Plasma Membrane | 1.979 | -0.521 | 3683 |
| *ITGAX* | integrin, alpha X (complement component 3 receptor 4 subunit) | Plasma Membrane | 1.004 | -0.758 | 3687 |
| *ITGB2* | integrin, beta 2 (complement component 3 receptor 3 and 4 subunit) | Plasma Membrane | 2.011 | 0.210 | 3689 |
| *LCP2* | lymphocyte cytosolic protein 2 (SH2 domain containing leukocyte protein of 76kDa) | Cytoplasm | 2.563 | -0.007 | 3937 |
| *LGALS3* | lectin, galactoside-binding, soluble, 3 | Extracellular Space | 1.208 | -0.580 | 3958 |
| *LY96* | lymphocyte antigen 96 | Plasma Membrane | 1.439 | -0.514 | 23643 |
| *MARCKSL1* | MARCKS-like 1 | Cytoplasm | 1.346 | 0.596 | 65108 |
| *MYH10* | myosin, heavy chain 10, non-muscle | Cytoplasm | -0.318 | 1.615 | 4628 |
| *MYL9* | myosin, light chain 9, regulatory | Cytoplasm | -0.960 | 0.297 | 10398 |
| *NCAM1* | neural cell adhesion molecule 1 | Plasma Membrane | -1.404 | 0.043 | 4684 |
| *NID1* | nidogen 1 | Extracellular Space | -0.571 | 1.461 | 4811 |
| *NTS* | neurotensin | Extracellular Space | -1.719 | -0.156 | 4922 |
| *PLEK* | pleckstrin | Cytoplasm | 2.425 | -0.651 | 5341 |
| *PRF1* | perforin 1 (pore forming protein) | Cytoplasm | 3.893 | 0.247 | 5551 |
| *PROS1* | protein S (alpha) | Extracellular Space | -2.500 | -0.423 | 5627 |
| *PSAP* | prosaposin | Extracellular Space | 1.333 | 0.269 | 5660 |
| *PSMD12* | proteasome (prosome, macropain) 26S subunit, non-ATPase, 12 | Cytoplasm | -1.315 | -0.088 | 5718 |
| *PSME1* | proteasome (prosome, macropain) activator subunit 1 (PA28 alpha) | Cytoplasm | 1.427 | -0.035 | 5720 |
| *S100A8* | S100 calcium binding protein A8 | Cytoplasm | 1.773 | 1.214 | 6279 |
| *S100A9* | S100 calcium binding protein A9 | Cytoplasm | 0.929 | 1.901 | 6280 |
| *SAA1* | serum amyloid A1 | Extracellular Space | 5.133 | -1.030 | 6288 |
| *SELL* | selectin L | Plasma Membrane | 0.549 | -0.373 | 6402 |
| *SERPINE1* | serpin peptidase inhibitor, clade E (nexin, plasminogen activator inhibitor type 1), member 1 | Extracellular Space | 1.570 | 2.893 | 5054 |
| *SH2D1A* | SH2 domain containing 1A | Cytoplasm | 2.628 | 0.558 | 4068 |
| *SOCS2* | suppressor of cytokine signaling 2 | Cytoplasm | -0.633 | 0.258 | 8835 |
| *SPARC* | secreted protein, acidic, cysteine-rich (osteonectin) | Extracellular Space | -0.521 | 0.597 | 6678 |
| *TAC1* | tachykinin, precursor 1 | Extracellular Space | -1.968 | -1.519 | 6863 |
| *TGFBI* | transforming growth factor, beta-induced, 68kDa | Extracellular Space | 0.994 | 1.314 | 7045 |
| *THBS1* | thrombospondin 1 | Extracellular Space | 0.529 | -1.698 | 7057 |
| *THBS2* | thrombospondin 2 | Extracellular Space | 0.388 | -0.970 | 7058 |
| *TIMP1* | TIMP metallopeptidase inhibitor 1 | Extracellular Space | 2.155 | 1.941 | 7076 |
| *TOLLIP* | toll interacting protein | Cytoplasm | -0.547 | 0.190 | 54472 |
| *TREM1* | triggering receptor expressed on myeloid cells 1 | Plasma Membrane | 0.406 | 1.314 | 54210 |
| *TXNIP* | thioredoxin interacting protein | Cytoplasm | -1.077 | 0.645 | 10628 |
| *VCAN* | versican | Extracellular Space | 2.419 | 0.591 | 1462 |
| *XAF1* | XIAP associated factor 1 | Nucleus | 1.507 | 0.076 | 54739 |
| **Peptidase** | |  |  |  |  |
| *C1R* | complement component 1, r subcomponent | Extracellular Space | 2.106 | -0.319 | 715 |
| *CAPN2* | calpain 2, (m/II) large subunit | Cytoplasm | -1.265 | -0.380 | 824 |
| *CASP1* | caspase 1, apoptosis-related cysteine peptidase | Cytoplasm | 1.277 | -0.135 | 834 |
| *CASP4* | caspase 4, apoptosis-related cysteine peptidase | Cytoplasm | 1.879 | -0.054 | 837 |
| *CASP5* | caspase 5, apoptosis-related cysteine peptidase | Cytoplasm | 1.879 | -0.054 | 838 |
| *CASP8* | caspase 8, apoptosis-related cysteine peptidase | Nucleus | 1.587 | 0.881 | 841 |
| *CFB* | complement factor B | Extracellular Space | 1.706 | -0.651 | 629 |
| *CTSB* | cathepsin B | Cytoplasm | 1.908 | -0.125 | 1508 |
| *CTSD* | cathepsin D | Cytoplasm | 2.049 | 1.997 | 1509 |
| *CTSH* | cathepsin H | Cytoplasm | 0.829 | -0.623 | 1512 |
| *CTSK* | cathepsin K | Cytoplasm | 2.765 | 0.565 | 1513 |
| *CTSS* | cathepsin S | Cytoplasm | 1.161 | -0.027 | 1520 |
| *GZMA* | granzyme A (granzyme 1, cytotoxic T-lymphocyte-associated serine esterase 3) | Cytoplasm | 5.773 | 0.681 | 3001 |
| *GZMB* | granzyme B (granzyme 2, cytotoxic T-lymphocyte-associated serine esterase 1) | Cytoplasm | 2.493 | -0.848 | 3002 |
| *MMP1* | matrix metallopeptidase 1 (interstitial collagenase) | Extracellular Space | 5.566 | -0.591 | 4312 |
| *NPEPPS* | aminopeptidase puromycin sensitive | Cytoplasm | -1.434 | -0.069 | 9520 |
| *PLAU* | plasminogen activator, urokinase | Extracellular Space | 2.000 | 0.315 | 5328 |
| *PSMB10* | proteasome (prosome, macropain) subunit, beta type, 10 | Cytoplasm | 2.156 | 0.547 | 5699 |
| *PSMB8* | proteasome (prosome, macropain) subunit, beta type, 8 (large multifunctional peptidase 7) | Cytoplasm | 1.761 | 0.267 | 5696 |
| *PSMB9* | proteasome (prosome, macropain) subunit, beta type, 9 (large multifunctional peptidase 2) | Cytoplasm | 2.820 | 0.467 | 5698 |
| *PSME2* | proteasome (prosome, macropain) activator subunit 2 (PA28 beta) | Cytoplasm | 1.626 | 0.090 | 5721 |
| **Phosphatase** | |  |  |  |  |
| *PPP3CA* | protein phosphatase 3, catalytic subunit, alpha isozyme | Cytoplasm | -1.015 | -0.485 | 5530 |
| *PTEN* | phosphatase and tensin homolog | Cytoplasm | -0.470 | 0.714 | 5728 |
| *PTPN1* | protein tyrosine phosphatase, non-receptor type 1 | Cytoplasm | 1.449 | 0.333 | 5770 |
| *PTPN2* | protein tyrosine phosphatase, non-receptor type 2 | Cytoplasm | 1.275 | 0.014 | 5771 |
| **Transcription regulators** | |  |  |  |  |
| *CIITA* | class II, major histocompatibility complex, transactivator | Nucleus | 1.704 | 0.037 | 4261 |
| *GATA3* | GATA binding protein 3 | Nucleus | 0.329 | 1.460 | 2625 |
| *HMGB1* | high mobility group box 1 | Nucleus | -0.411 | 0.462 | 3146 |
| *IRF5* | interferon regulatory factor 5 | Nucleus | 2.509 | 0.297 | 3663 |
| *IRF7* | interferon regulatory factor 7 | Nucleus | 2.250 | 0.528 | 3665 |
| *IRF8* | interferon regulatory factor 8 | Nucleus | 2.471 | 0.889 | 3394 |
| *NFKB2* | nuclear factor of kappa light polypeptide gene enhancer in B-cells 2 (p49/p100) | Nucleus | 1.691 | 0.024 | 4791 |
| *NFKBIE* | nuclear factor of kappa light polypeptide gene enhancer in B-cells inhibitor, epsilon | Nucleus | 2.312 | -0.295 | 4794 |
| *PYCARD* | PYD and CARD domain containing | Cytoplasm | 1.148 | 0.294 | 29108 |
| **Transmembrane receptors** | |  |  |  |  |
| *B2M* | beta-2-microglobulin | Plasma Membrane | 2.093 | 2.559 | 567 |
| *CD14* | CD14 molecule | Plasma Membrane | 1.812 | 0.423 | 929 |
| *CD2* | CD2 molecule | Plasma Membrane | 3.389 | 0.122 | 914 |
| *CD36* | CD36 molecule (thrombospondin receptor) | Plasma Membrane | -1.520 | 0.572 | 948 |
| *CD3G* | CD3g molecule, gamma (CD3-TCR complex) | Plasma Membrane | 2.850 | 1.223 | 917 |
| *CD74* | CD74 molecule, major histocompatibility complex, class II invariant chain | Plasma Membrane | 0.571 | -0.381 | 972 |
| *CD86* | CD86 molecule | Plasma Membrane | 2.074 | -0.125 | 942 |
| *CR1* | complement component (3b/4b) receptor 1 (Knops blood group) | Plasma Membrane | 1.125 | 0.054 | 1378 |
| *DAG1* | dystroglycan 1 (dystrophin-associated glycoprotein 1) | Plasma Membrane | -1.371 | -0.450 | 1605 |
| *EDNRA* | endothelin receptor type A | Plasma Membrane | -0.071 | 0.997 | 1909 |
| *EPOR* | erythropoietin receptor | Plasma Membrane | -0.184 | 0.954 | 2057 |
| *F3* | coagulation factor III (thromboplastin, tissue factor) | Plasma Membrane | -1.929 | -0.193 | 2152 |
| *FAS* | Fas (TNF receptor superfamily, member 6) | Plasma Membrane | 1.579 | 0.052 | 355 |
| *FCGR2A* | Fc fragment of IgG, low affinity IIa, receptor (CD32) | Plasma Membrane | 3.571 | -0.272 | 2212 |
| *FCGR2B* | Fc fragment of IgG, low affinity IIb, receptor (CD32) | Plasma Membrane | 3.571 | -0.272 | 2213 |
| *FCGR3A* | Fc fragment of IgG, low affinity IIIa, receptor (CD16a) | Plasma Membrane | 1.605 | -0.569 | 2214 |
| *HLA-DMA* | major histocompatibility complex, class II, DM alpha | Plasma Membrane | 2.052 | 0.244 | 3108 |
| *HLA-DMB* | major histocompatibility complex, class II, DM beta | Plasma Membrane | 1.799 | -0.004 | 3109 |
| *HLA-DQA1* | major histocompatibility complex, class II, DQ alpha 1 | Plasma Membrane | 0.859 | -0.401 | 3117 |
| *HLA-DRA* | major histocompatibility complex, class II, DR alpha | Plasma Membrane | 2.012 | -0.307 | 3122 |
| *HLA-F* | major histocompatibility complex, class I, F | Plasma Membrane | 2.556 | 0.933 | 3134 |
| *IGF1R* | insulin-like growth factor 1 receptor | Plasma Membrane | 0.543 | 1.641 | 3480 |
| *IL1R2* | interleukin 1 receptor, type II | Plasma Membrane | 1.500 | 0.229 | 7850 |
| *IL23R* | interleukin 23 receptor | Plasma Membrane | 2.391 | 0.817 | 149233 |
| *IL2RA* | interleukin 2 receptor, alpha | Plasma Membrane | 1.828 | 0.302 | 3559 |
| *IL4R* | interleukin 4 receptor | Plasma Membrane | 0.865 | 2.755 | 3566 |
| *IL6R* | interleukin 6 receptor | Plasma Membrane | 0.500 | 1.847 | 3570 |
| *ITGB1* | integrin, beta 1 (fibronectin receptor, beta polypeptide, antigen CD29 includes MDF2, MSK12) | Plasma Membrane | -0.563 | 0.487 | 3688 |
| *ITGB3* | integrin, beta 3 (platelet glycoprotein IIIa, antigen CD61) | Plasma Membrane | 0.592 | 1.587 | 3690 |
| *ITGB7* | integrin, beta 7 | Plasma Membrane | 1.965 | 0.876 | 3695 |
| *LTBR* | lymphotoxin beta receptor (TNFR superfamily, member 3) | Plasma Membrane | 0.455 | 3.180 | 4055 |
| *NCR1* | natural cytotoxicity triggering receptor 1 | Plasma Membrane | 2.664 | -0.434 | 9437 |
| *OLR1* | oxidized low density lipoprotein (lectin-like) receptor 1 | Plasma Membrane | 0.958 | -0.618 | 4973 |
| *TLR2* | toll-like receptor 2 | Plasma Membrane | 3.517 | 0.653 | 7097 |
| *TLR4* | toll-like receptor 4 | Plasma Membrane | 1.376 | 0.067 | 7099 |
| **Transporters** | |  |  |  |  |
| *A2M* | alpha-2-macroglobulin | Extracellular Space | 1.169 | 0.575 | 2 |
| *KPNB1* | karyopherin (importin) beta 1 | Nucleus | 0.690 | -0.280 | 3837 |
| *RACGAP1* | Rac GTPase activating protein 1 | Cytoplasm | 1.219 | -0.122 | 29127 |
| *RAMP1* | receptor (G protein-coupled) activity modifying protein 1 | Plasma Membrane | -0.272 | 1.228 | 10267 |
| *SFTPA1* | surfactant protein A1 | Extracellular Space | 1.693 | 2.369 | 653509 |
| *SLC7A5* | solute carrier family 7 (amino acid transporter light chain, L system), member 5 | Plasma Membrane | 2.164 | -0.108 | 8140 |
| *TAP1* | transporter 1, ATP-binding cassette, sub-family B (MDR/TAP) | Cytoplasm | 3.175 | 0.371 | 6890 |
| *TAP2* | transporter 2, ATP-binding cassette, sub-family B (MDR/TAP) | Cytoplasm | 1.835 | 0.590 | 6891 |
| *TFRC* | transferrin receptor (p90, CD71) | Plasma Membrane | 1.241 | -1.046 | 7037 |
