# Supplementary figures and images for: Early innate immunity determines outcome of Mycobacterium tuberculosis pulmonary infection in rabbits
Source: Cell Commun Signal. 2013 Aug 19;11:60. doi: 10.1186/1478-811X-11-60 (PMC3765177; doi:10.1186/1478-811X-11-60)

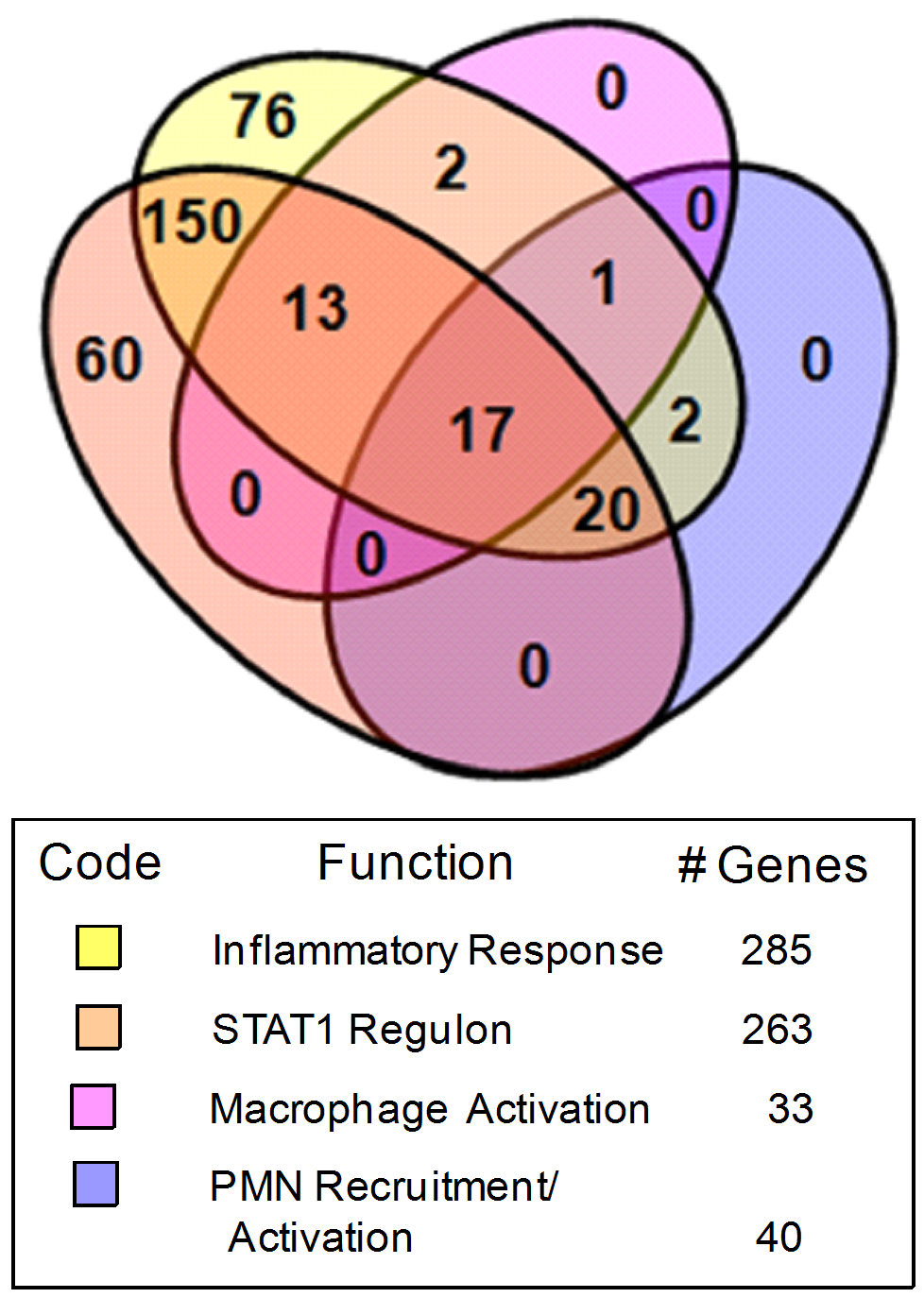

Supplement: Additional file 7: Figure S1 — Venn diagram showing distribution of the SDEG among the selected networks in the lungs of Mtb-infected rabbits at 3 hours. [file 1478-811X-11-60-S7.tiff]

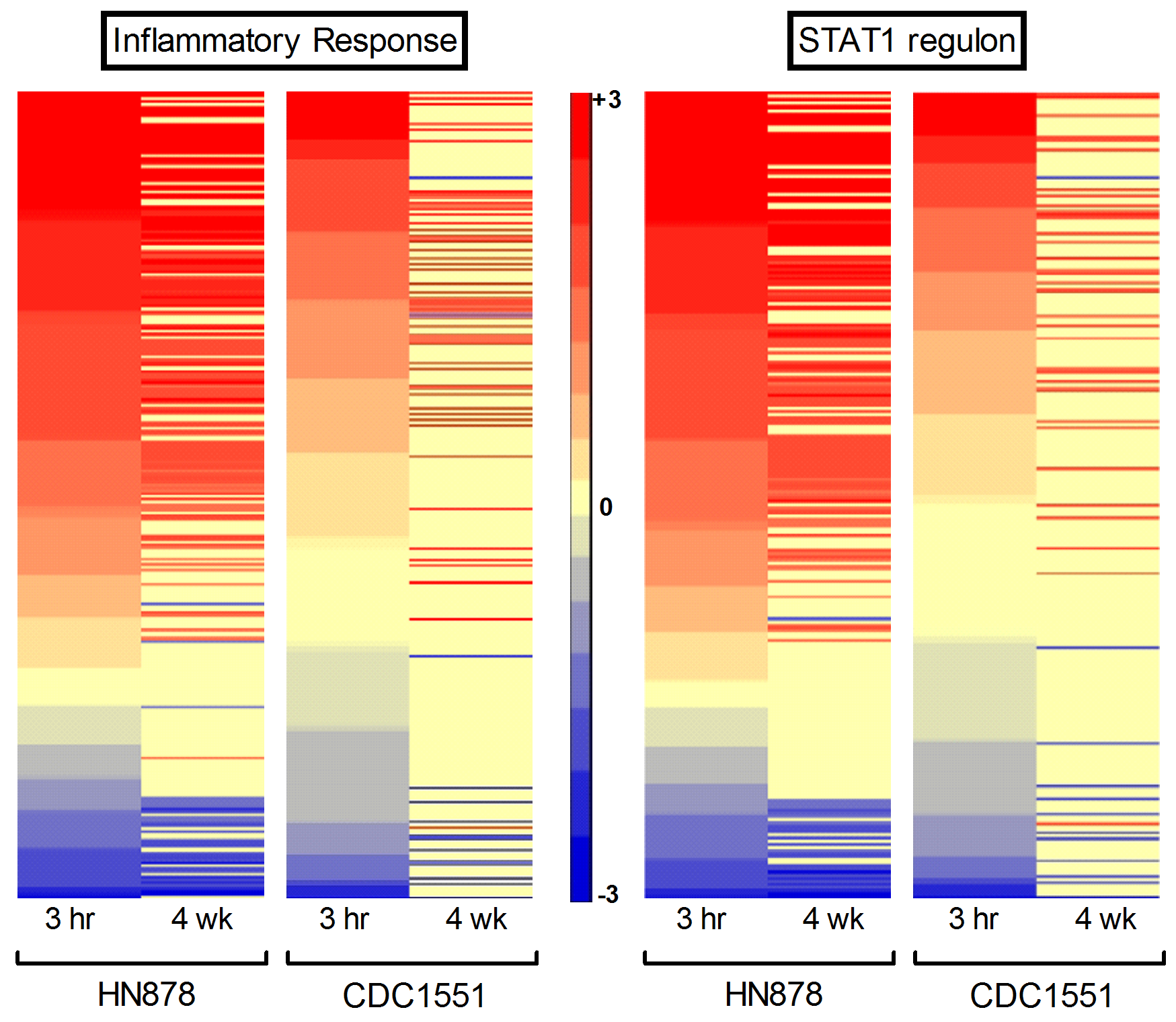

Supplement: Additional file 8: Figure S2 — Expression of inflammatory response and STAT1 activation network genes in Mtb-HN878- or CDC1551 infected-rabbit lungs at 3 hours and 4 weeks. [file 1478-811X-11-60-S8.tiff]
