# Supplementary material for: Early innate immunity determines outcome of Mycobacterium tuberculosis pulmonary infection in rabbits
Source: Cell Commun Signal. 2013 Aug 19;11:60. doi: 10.1186/1478-811X-11-60 (PMC3765177; doi:10.1186/1478-811X-11-60)
Supplement: Additional file 9 — Methods. Updated annotation of rabbit gene probes for microarray and Statistical analysis of microarray data. [file 1478-811X-11-60-S9.doc]

**Additional Material**

**Materials and Methods**

**Updated Annotation of Rabbit Gene Probes for Microarray**

**The 4X44K Agilent rabbit microarray (**[Platform GPL13288](http://0-www.ncbi.nlm.nih.gov.ilsprod.lib.neu.edu/geo/query/acc.cgi?acc=GPL13288)**) contains 43,803 probes, of which 43,603 are unique. The annotation provided by the array manufacturer (https://earray.chem.agilent.com/earray/) contains gene symbols for only 4918 unique probes. In this study, we have updated the rabbit microarray probe annotations to map 35,076 unique probes (80.4%) to 13,478 unique human gene symbols using Ensembl (human and rabbit) and Broad Institute (rabbit) transcript databases. We have updated the rabbit microarray annotation in four steps; with only probes not annotated in the previous step proceeding to the next step: (1) All 43,603 unique probes were aligned to 24,813 predicted rabbit transcripts from Ensembl (release 70) using megablast with a 95% sequence identity (SI) threshold**. **The Ensembl transcripts with the best bit score were identified and mapped through Ensembl to human gene symbols, providing symbols for 27,338 probes. (2) We aligned the remaining 16,265 probe sequences with the rabbit transcript database from Broad Institute using megablast with a 95% SI threshold, and the transcripts with the best bit score were retained. These rabbit transcripts were then realigned with the Ensembl rabbit transcripts and the matches with the best bit score and 95% SI were mapped through Ensembl to human orthologs with gene symbols, providing symbols for 6,159 (out of 16,265) probes. (3) The Broad Institute rabbit transcripts for the remaining 10,106 probes were aligned directly with the Ensembl human transcripts and those with the best bit score and 90% SI threshold were assigned human gene symbols, yielding symbols for 1,352 (out of 10,106) probes. (4) The 8,754 remaining probes were aligned directly with the human transcripts from Ensembl and the best bit scores at 90% SI were identified. The symbols from these human transcripts provided annotations for 227 probes. Of the remaining 8,527 unique probes not annotated by our procedure, 695 had a symbol in the Agilent database (**<https://earray.chem.agilent.com/earray/>**).**

**Statistical Analysis of Microarray Data**

**The microarray data were split into 3 classes, HN878 infected, CDC551- nfected, and uninfected and used in a one-way ANOVA test of the null hypothesis of equal mean of log-transformed intensities among the 3 classes. While the HN878 and CDC1551 data sets were treated as independent observations; the uninfected data sets were averaged and treated as a single observation since these samples were pooled to get the data. Uninfected data sets were averaged because they represent technical rather than biological replicates, and technical variance is often smaller than biological variance. The mean variance of probes across the 4 HN878 infected biological replicates was 0.133; the variance across the 4 CDC1551 infected biological replicates was 0.0787; and across the 8 uninfected technical replicates was 0.0715. Under a model in which the HN878 and CDC1551 variance includes both technical and biological components, while the uninfected variance is technical only, the ratio of technical to biological variance is 2.93. Thus, it may be appropriate to treat the 8 uninfected data sets equivalently with the biological replicates. Nevertheless, we assumed a conservative approach using the mean of the 8 uninfected data sets as a single observation. One-way ANOVA for the 3 groups and 9 samples was performed with variance stabilization to yield overall F-statistics and p-values, as well as F-statistics and p-values for the three pair-wise comparisons (lmFit, contrasts.fit, eBayes from Bioconductor limma package). A set of 50 probes was replicated 5 times on the array; these were assigned their median p-value. For symbols assigned to multiple probes, the probe with the smallest p-value was retained, with 13,478 unique genes and probes retained in all. Permutation tests established with a 0.05 family-wise error rate (0.05 FWER) was used to identify transcriptome-wide significantly differentially expressed genes (SDEG). Permutations were balanced by selecting either a true HN878 or CDC1551 sample as the single “Uninfected” sample, creating one “infected” group with 2 HN878 samples and 2 CDC1551 samples, and creating the second “infected” group with the true uninfected sample and the remaining 3 HN878 or CDC1551 infected samples. The total number of permutations is C (4,2)C(4,2)C(4,1) = 144. For each of these 144 permutations, the best p-value and F-statistic across all 13,478 genes was retained. The 5% threshold of the best p-value (calculated using R quantile function) was 2.9x10^–6^, close to the value of 0.05/13,478 = 3.7x10^–6^ from the number of tests. Although in principle the three pair-wise comparisons should have three-fold smaller thresholds, for simplicity the ANOVA thresholds were used for the pair-wise tests as well.**
