# Supplementary material for: Early innate immunity determines outcome of Mycobacterium tuberculosis pulmonary infection in rabbits
Source: Cell Commun Signal. 2013 Aug 19;11:60. doi: 10.1186/1478-811X-11-60 (PMC3765177; doi:10.1186/1478-811X-11-60)
Supplement: Additional file 10: Table S1 — List of oligonucleotide primers used for qRT-PCR experiments. [file 1478-811X-11-60-S10.doc]

**Supplementary Table S1.** List of oligonucleotide primers used for qRT-PCR experiments

| **Gene Symbol** | **Direction** | **Primers (5' -> 3')** | **Description** | **Gene ID** |
| --- | --- | --- | --- | --- |
| *TNF* | Forward | CTGAGTGACGAGCCTCTAGC | Tumor necrosis factor-alpha | NM_001082263 |
|  | Reverse | TTCATGCCGTTGGCCAGCAG |  |  |
| *GAPDH* | Forward | GGCGTGAACCACGAGAAGTA | Glyceraldehyde 3-phosphase dehydrogenase | 100009074 |
|  | Reverse | TCCACAATGCCGAAGTGGTC |  |  |
| *IL4R* | Forward | TTACCTGGCAGGACCAGTAT | Interleukin 4 receptor | 100337721 |
|  | Reverse | GTCACGTTGACGCTGAAGAT |  |  |
| *CAV1* | Forward | GCGACCCCAAGCATCTCA | Caveolin 1 | 100008837 |
|  | Reverse | GATGGTAGACAGTAGGCG |  |  |
| *CCL4* | Forward | TCCTCGTCACTTCGTGATAG | C-C- chemokine ligand-4 | 100008984 |
|  | Reverse | TCCAAGTCATCCACGTACTC |  |  |
| *CD36* | Forward | AGAGGTCCTTACACGTACAG | Cluster of difference 36 | 100008825 |
|  | Reverse | GTCATTCTCGGTTCCAACAG |  |  |
| *CCL2(MCP1)* | Forward | TTCTGTGCCTGCTGCTCATA | Macrophage cationic peptide 1 | 100009115 |
|  | Reverse | GGACACTTGGTGCTGTTGAT |  |  |
| *IL18* | Forward | ATGGCTGCTGAACCAGAAGA | Interleukin 18 | 100144338 |
|  | Reverse | TTGCTGTCCTTGGTCCATGA |  |  |
| *CXCL10* | Forward | CTGTACGCTGTACCTGTATC | Chemokine C-X-C- ligand 10 | 100353112 |
|  | Reverse | GCAGTGGTCCATTCTCATCA |  |  |
| *IL1A* | Forward | GGAATACAGCTCTGCCATTG | Interleukin 1 alpha | 100009250 |
|  | Reverse | GTTACTGCCACCACATTCTC |  |  |
| *TGFB2* | Forward | GAAAGACCCCACATCTCCTG | Transforming growth factor, beta 2 | 100009371 |
|  | Reverse | CATCCAATGCACGCTTCTTC |  |  |
| *SPP1* | Forward | TCTCCTAACACCGCAGAATG | Secreted phosphoprotein 1 | 100008982 |
|  | Reverse | TCTGTAAGCCACACTGTCAC |  |  |
| *IRF5* | Forward | AGCCAGGATGGAGACAACAC | Interferon regulatory factor 5 | 100350244 |
|  | Reverse | CATAGATGAGGCGGAAGTCG |  |  |
| *CD38* | Forward | CAACCTTGTGTGGTGTGGAG | Cluster of difference 38 | 100009409 |
|  | Reverse | GTTCTGGACCCATTGAGCAT |  |  |
| *STAT1* | Forward | CTGTGAAGCTGAGGCTGTTG | Signal transducer and activator of transcription | 100343893 |
|  | Reverse | GTTGGTGGACTCCTCCATGT |  |  |
